# Supplementary material for: Sepsis subphenotyping based on organ dysfunction trajectory
Source: Crit Care. 2022 Jul 3;26:197. doi: 10.1186/s13054-022-04071-4 (PMC9250715; doi:10.1186/s13054-022-04071-4)
Supplement: Supplementary file 1 — Additional file 1: Sepsis Subphenotyping Based on Organ Dysfunction Trajectory. [file 13054_2022_4071_MOESM1_ESM.pdf]

Supplementary information to:

Title Sepsis Subphenotyping Based on Organ Dysfunction Trajectory

Authors

Zhenxing Xu, PhD[1]
Chengsheng Mao, PhD[2]
Chang Su, PhD[3]
Hao Zhang, PhD[1]
Ilias Siempos, MD[4,5]
Lisa K Torres, MD[4,5]
Di Pan, DO[4,5]
Yuan Luo, PhD[2]
Edward J Schenck, MD[4,5]
Fei Wang, PhD[1]

Affiliations

[1]Division of Health Informatics, Department of Population Health Sciences, Weill Cornell Medicine, New York, NY, USA;
[2]Division of Health and Biomedical Informatics, Department of Preventive Medicine Center for Health Information Partnerships, Feinberg School of Medicine, Northwestern University, Chicago, IL, USA;
[3]Department of Health Service Administration and Policy (HSAP), College of Public Health, Temple University, Philadelphia, PA, USA;
[4]Division of Pulmonary and Critical Care Medicine, NewYork-Presbyterian Hospital/Weill Cornell Medical Center, New York, NY, USA;
[5]Weill Cornell Medical College, Weill Cornell Medicine, New York, NY, USA.

Corresponding Authors Information

Fei Wang: 425 E. 61st Street, 3rd Floor, Suite 301, New York, NY. few2001@med.cornell.edu;
Edward J Schenck: 425 E. 61st Street, 4th Floor, Suite 402, New York, NY. ejs9005@med.cornell.edu;
Yuan Luo: Rubloff Building 11th Floor, 750 N Lake Shore, Chicago Illinois. yuan.luo@northwestern.edu.

Supplementary Materials

Tables

Table S1. Patient characteristics comparisons between survivors and nonsurvivors in the development cohort

Table S2. Patient characteristics comparisons between survivors and nonsurvivors in the NMEDW validation cohort

Table S3. Patient characteristics comparisons between survivors and nonsurvivors in the eICU validation cohort

Table S4. Patient characteristics comparisons between survivors and nonsurvivors in the CEDAR validation cohort

Table S5. Multiple clustering metrics statistics and cluster number determination

Table S6. Patient physiological characteristics comparisons among subphenotypes in the development cohort

Table S7. Patient basic characteristics comparisons among subphenotypes in the NMEDW validation cohort

Table S8. Patient physiological characteristics comparisons among subphenotypes in the NMEDW validation cohort

Table S9. Patient basic characteristics comparisons among subphenotypes in the eICU validation cohort

Table S10. Patient physiological characteristics comparisons among subphenotypes in the eICU validation cohort

Table S11. Patient basic characteristics comparisons among subphenotypes in the CEDAR validation cohort

Table S12. Patient physiological characteristics comparisons among subphenotypes in the CEDAR validation cohort

Table S13. Patient physiological characteristics comparisons among subphenotypes in terms of sensitivity analysis (GBTM) in the development cohort

Table S14. Missing data (no., %) in cohorts for subphenotyping and predicting variables.

Table S15. International classification of diseases, ninth revision, clinical modification codes used to identify infectious syndromes.

Table S16. The associations between comorbidities and subphenotypes in the MIMIC-III development cohort

Table S17. The associations between comorbidities and subphenotypes in the NMEDW validation cohort

Table S18. The associations between comorbidities and subphenotypes in the eICU validation cohort

Table S19. The associations between comorbidities and subphenotypes in the CEDAR validation cohort

Figures

Figure S1. Patient exclusion criteria for building development and validation cohorts

Figure S2. Clustergrams of hierarchical clustering. Horizontal and vertical axes represent patients. Color intensity denotes normalized pairwise patient similarity derived using Dynamic Time Warping (DTW). All clustergrams suggest optimal cluster number 4.

Figure S3. The trajectories of the subphenotypes in terms of subscores in the development cohort. DI: Delayed Improving; RI: Rapidly Improving; DW: Delayed Worsening; RW: Rapidly Worsening.

Figure S4. The trajectories of the subphenotypes in terms of subscores in the NMEDW validation cohort. DI: Delayed Improving; RI: Rapidly Improving; DW: Delayed Worsening; RW: Rapidly Worsening.

Figure S5. Predictor contribution to the prediction of the predictive model in the NMEDW validation cohort. DI: Delayed Improving; RI: Rapidly Improving; DW: Delayed Worsening; RW: Rapidly Worsening.

Figure S6. Chord diagrams showing abnormal clinical variables by subphenotype in the NMEDW validation cohort. a: abnormal biomarkers vs. all subphenotypes; I: abnormal biomarkers vs. DI; II: abnormal biomarkers vs. RI; III: abnormal biomarkers vs. DW; IV: abnormal biomarkers vs. RW. DI: Delayed Improving; RI: Rapidly Improving; DW: Delayed Worsening; RW: Rapidly Worsening.

Figure S7. Chord diagrams showing abnormal subscores by subphenotype in the NMEDW validation cohort. a: abnormal subscores vs. all subphenotypes; I: abnormal subscores vs. DI; II: abnormal subscores vs. RI; III: abnormal subscores vs. DW; IV: abnormal subscores vs. RW. DI: Delayed Improving; RI: Rapidly Improving; DW: Delayed Worsening; RW: Rapidly Worsening.

Figure S8 Sequential Organ Failure Assessment (SOFA) trajectories of the subphenotypes and survival analysis in terms of the identified subphenotypes by GBTM. The (A) describes the SOFA trajectories of the subphenotypes re-derived by GBTM in development cohort. The (B) shows the survival analysis results in subphenotypes re-derived by GBTM in the development cohort. DI: Delayed Improving; RI: Rapidly Improving; DW: Delayed Worsening; RW: Rapidly Worsening.

Figure S9. Confusion matrices for comparing the subphenotypes obtained by DTW and HAC, and GBTM in development cohort. DI: Delayed Improving; RI: Rapidly Improving; DW: Delayed Worsening; RW: Rapidly Worsening. DTW: Dynamic Time Warping; HAC: Hierarchical Agglomerative Clustering. GBTM: Group-Based Trajectory Modeling.

Figure S10. The trajectories of the subphenotypes in terms of subscores in the eICU validation cohort. DI: Delayed Improving; RI: Rapidly Improving; DW: Delayed Worsening; RW: Rapidly Worsening.

Figure S11. Predictor contribution to the prediction of the predictive model in the eICU validation cohort. DI: Delayed Improving; RI: Rapidly Improving; DW: Delayed Worsening; RW: Rapidly Worsening.

Figure S12. Chord diagrams showing abnormal clinical variables by subphenotype in the eICU validation cohort. a: abnormal biomarkers vs. all subphenotypes; I: abnormal biomarkers vs. DI; II: abnormal biomarkers vs. RI; III: abnormal biomarkers vs. DW; IV: abnormal biomarkers vs. RW. DI: Delayed Improving; RI: Rapidly Improving; DW: Delayed Worsening; RW: Rapidly Worsening.

Figure S13. Chord diagrams showing abnormal subscores by subphenotype in the eICU validation cohort. a: abnormal subscores vs. all subphenotypes; I: abnormal subscores vs. DI; II: abnormal subscores vs. RI; III: abnormal subscores vs. DW; IV: abnormal subscores vs. RW. DI: Delayed Improving; RI: Rapidly Improving; DW: Delayed Worsening; RW: Rapidly Worsening.

Figure S14. The trajectories of the subphenotypes in terms of subscores in the CEDAR validation cohort. DI: Delayed Improving; RI: Rapidly Improving; DW: Delayed Worsening; RW: Rapidly Worsening.

Figure S15. Predictor contribution to the prediction of the predictive model in the CEDAR validation cohort. DI: Delayed Improving; RI: Rapidly Improving; DW: Delayed Worsening; RW: Rapidly Worsening.

Figure S16. Chord diagrams showing abnormal clinical variables by subphenotype in the CEDAR validation cohort. a: abnormal biomarkers vs. all subphenotypes; I: abnormal biomarkers vs. DI; II: abnormal biomarkers vs. RI; III: abnormal biomarkers vs. DW; IV: abnormal biomarkers vs. RW. DI: Delayed Improving; RI: Rapidly Improving; DW: Delayed Worsening; RW: Rapidly Worsening.

Figure S17. Chord diagrams showing abnormal subscores by subphenotype in the CEDAR validation cohort. a: abnormal subscores vs. all subphenotypes; I: abnormal subscores vs. DI; II: abnormal subscores vs. RI; III: abnormal subscores vs. DW; IV: abnormal subscores vs. RW. DI: Delayed Improving; RI: Rapidly Improving; DW: Delayed Worsening; RW: Rapidly Worsening.

Figure S18. The accuracy of predicting four subphenotypes at successive time points (hours 6, 24, 36, 48, 60) after ICU admission in development and validation cohorts.

Figure S19. The illustration of using DTW during obtaining matched sequences. (c) shows the warping path induced by the DTW matching, where the blue cells indicate the best matching pairs for the points in the two sequences.

Figure S20. An example: the illustration of patient's SOFA trajectory.

Appendix 1 The detailed description in terms of cohorts

Study data used in our study were from the Medical Information Mart for Intensive Care III (MIMIC-III) database. The collection of MIMIC-III dataset was passive and de-identified, which was in compliance with the Health Insurance Portability and Accountability Act (HIPAA) Privacy Rule and did not produce significant impacts on patient safety. This database consisted of patients admitted to the Beth Israel Deaconess Medical Center (BIDMC, Boston, MA). This database included information such as patient demographics, vital signs, laboratory test results. A total of 23,620 ICU admissions in the Meta Vision system of MIMIC-III were analyzed. We excluded some patients such as nonadults, secondary (or greater) admissions for patients to avoid repeated measures, admissions with missing data. The final cohort contained 4,678 patients for further analysis. In addition, in the first validation cohort that is built based on patient's records from Northwestern Medicine Enterprise Data Warehouse (NMEDW) from 2012 to 2019, a total of 67,850 ICU admissions were analyzed. We finally obtained 3,665 patients for further analysis. NMEDW contains 11 hospitals, including Northwestern Memorial Hospital, Northwestern Medicine Central DuPage Hospital, etc., more details can be found in [1]. For the second validation cohort, eICU Collaborative Research Database (eICU-CRD) [9], which is a deidentified and publicly available data set that meets the safe harbor provision of the US Health Insurance Portability and Accountability Act (HIPAA). The eICU-CRD is a multi-center database sourced from the Philips eICU program, a telemedicine initiative where healthcare workers remotely monitor acutely ill patients. It comprises 200,859 patient unit encounters for 139,367 unique patients admitted between 2014 and 2015 across the United States. The patient information includes demographics, vital sign measurements, care plan documentation, the severity of illness measures, diagnosis information, treatment information, and more. For the third validation cohort, the Critical carE Database for Advanced Research (CEDAR) [10], which was built on NewYork-Presbyterian/Weill Cornell Medical Center (NYP/WCMC) from admissions dating from 2001-2020. The NYP/WCMC was an academic medical center in New York City located on the Upper East Side of Manhattan, including 862 beds in total and 118 beds for ICU units across dedicated cardiac, cardiothoracic surgery, medical, surgical, pediatric, and neurosurgical units. Figure S1 descripted the inclusion and exclusion criteria for the selection process. In particular, for each database, we excluded non-adult (Age<18). We excluded secondary (or greater) admissions for patients to avoid repeated measures. We excluded admissions to the cardiothoracic surgical service and admissions with missing data. We excluded patients if there were not met Sepsis-3 criteria in the first 6 hours since ICU admission. Specifically, we included patients if they had evidence of a suspected infection defined as the combination of administration of antibiotics (oral or parenteral) and a body fluid culture specimen obtained (blood, urine, body fluid or cerebrospinal fluid), the first of which was required within the first 6 hours of ICU presentation. For SOFA score rule, we used an absolute SOFA score of >=2. Two sepsis studies based on Sepsis-3 definition [15][16] were referred to during selecting patients in each database. The SOFA score was computed from six organ-specific subscores including respiration, coagulation, liver, cardiovascular, CNS, renal. They were computed based on parameters including PaO2, FiO2, platelets, bilirubin, MAP, dopamine, dobutamine, epinephrine, norepinephrine, GCS, creatinine, urine output. The detailed scoring rule can be referred to Singer et.al study [18].

Appendix 2 The detailed description about statistical analysis variables and test techniques

(1) Demographics: age, gender, and race.
(2) Biomarkers: C-reactive protein (CRP), bands, temperature, white blood cell count (WBC), SO2, PaO2, respiratory rate, bicarbonate, heart rate, lactate, systolic blood pressure, troponin T or I, blood urea nitrogen (BUN), creatinine, alanine aminotransferase (ALT), aspartate aminotransferase (AST), bilirubin, Glasgow coma scale score (GCS), hemoglobin, INR, platelets; albumin, chloride, glucose, sodium, red blood cell distribution width (RDW).
(3) The type of ICU unit: TSICU (Thoracic Surgery ICU), MICU (Medical ICU), SICU (Surgical ICU), CCU (Coronary Care Unit), CSRU (Cardiac Surgery ICU).
(4) Infection sources: Central nervous system, Intra-Abdominal, Pneumonia, Septicemia bacteremia, Skin soft tissue, Urinary tract. These infection items are defined based on ICD-9 code (see Table S15). More details can be referred to [17].
(5) Admission location: Transfer from other hospital, Emergency room, Clinic referral, Transfer from ward, Physician referral, Transfer from skilled nursing facility.
(6) Multiple statistical test techniques were performed to discover association analyses in terms of those variables. Specifically, one-way analysis of variance (ANOVA, with Tukey HSD post hoc test), Kruskal–Wallis test (with Dunn post hoc test), student's t-test, Mann-Whitney test, Chi-square test, and Fisher's exact test were considered to use when appropriate. In addition, multiple testing utilizing false discovery rate (FDR) estimation was considered to correct the p-values. The techniques of analysis of covariance

(ANCOVA) were used for the between-subphenotype comparisons, which were based on general linear model.

Appendix 3 The detailed analysis in terms of subphenotypes in the validation cohorts
In the NMEDW validation cohort, four subphenotypes ("Delayed Improving", n=668, 18.2%), ("Rapidly Improving", n=2,019, 55.1%), ("Delayed Worsening", n=302, 8.2%), ("Rapidly Worsening" (n=676, 18.4%). In the eICU validation cohort, four subphenotypes ("Delayed Improving", n=2005, 16.3%), ("Rapidly Improving", n=5938, 48.4%), ("Delayed Worsening", n=1392, 11.3%), ("Rapidly Worsening" (n=2947, 23.9%). In the CEDAR cohort, four subphenotypes ("Delayed Improving", n=382, 7.9%), ("Rapidly Improving", n=2561, 53.3%), ("Delayed Worsening", n=564, 11.7%), ("Rapidly Worsening" (n=1297, 26.9%). We obtained the similar results with development cohort. Some specific similar and different characteristics in terms of trends derived on development and validation cohorts are summarized as follows: (1) four similar trend were obtained, including increasing continuously, decreasing firstly and keeping stable and slight increasing finally, decreasing sharply, increasing firstly and then decreasing gradually; (2) most of the patients kept decreasing and stable (e.g., "Delayed Improving", "Rapidly Improving", and "Delayed Worsening"). (3) The range of SOFA scores at baseline among these subphenotypes were different. For the development cohort, the range of average SOFA score at the baseline among the four subphenotypes was from 4.0 to 5.5. For the NMEDW validation cohort, the range was from 4.7 to 6.1. For the eICU validation cohort, the range was from 5.4 to 6.4. The fluctuation in these cohorts was smaller than the SOFA score in the development cohort. For the CEDAR validation cohort, the range was from 5.1 to 6.9. The fluctuation in this cohort was larger than the SOFA score in the development cohort and other validation cohorts (NMEDW and eICU).

Appendix 4 The detailed analysis in terms of physiological characteristics
Patients physiological characteristics mainly covered inflammation, pulmonary, cardiovascular/Hemodynamic, renal, hepatic, neurological, and hematologic. Specifically, vital signs including temperature, respiratory rate, heart rate, GCS, systolic ABP, and BMI. Lab tests including bands, C-reactive protein, WBC, SO2, PaO2, Bicarbonate, lactate, troponin, BUN, creatinine, ALT, AST, bilirubin, hemoglobin, INR, platelets, albumin, chloride, glucose, sodium, lymphocyte count, RDW, and lymphocyte percentage. We screened the vital signs and lab tests from their values in the first 6 hours to compare these subphenotypes. For the development cohort, the significant lab tests included ALT, AST, bicarbonate, bilirubin, BUN, chloride, creatinine, hemoglobin, INR, lactate, platelet, SO2, WBC, RDW. The significant vital signs included respiratory rate, heart rate, systolic ABP, temperature. Patients in Rapidly Worsening had the highest ALT, AST, bilirubin, heart rate, INR, lactate and the lowest bicarbonate and platelet. Patients in Rapidly Improving had the highest hemoglobin, platelet, WBC, temperature and the lowest heart rate, RDW, SO2, and systolic ABP. More information was shown in Table S6. For the NMEDW validation dataset, the significant lab tests and vital signs include systolic ABP, platelet, RDW, AST, GCS, respiratory rate, WBC. For the eICU validation dataset, the significant lab tests and vital signs include Albumin, ALT, AST, Bicarbonate, Bilirubin, BMI, BUN, Chloride, Creatinine, GCS, Glucose, Heart rate, INR, Lactate, Lymphocyte, Platelet, RDW, respiratory rate, SO2, systolic ABP, and WBC. For the CEDAR validation dataset, the significant lab tests and vital signs include Albumin, Bicarbonate, BUN, Creatinine, GCS, Glucose, Heart rate, Hemoglobin, INR, Lactate, Platelet, Respiratory rate, SO2, systolic ABP, Temperature, and WBC. More details are shown in Tables S8, S10 and S12.

Appendix 5 Chord plots
Chord plots were used to visualize the patterns of abnormal clinical variables and six sub-SOFA scores by subphenotype, which was obtained using the "Circlize" package in R. These clinical variables were divided into several groups. In particular,

- Inflammatory markers: temperature, WBC, bands, CRP, albumin, lymphocyte percent.
- Hepatic markers: bilirubin, AST, ALT.
- Cardiovascular markers: heart rate, systolic ABP, bicarbonate, troponin T or I, lactate.
- Renal markers: creatinine, BUN, chloride, sodium.
- Hematologic markers: hemoglobin, INR, platelet, glucose, RDW.
- Pulmonary markers: respiratory rate, SO2, Pao2.
- Neurologic marker: GCS.
- Comorbidity: Comorbidity score based on Elixhauser.

For each subphenotype, if a clinical variable median value (e.g., AST) is greater than the median of the development cohort, a ribbon is connected between the subphenotype and the group (e.g., Hepatic markers). Subphenotypes are demonstrated in different colors. If there are more clinical variables abnormal for that subphenotype, the ribbon would be more broader.

Appendix 6 The determination of the number of optimal clusters
Multiple clustering indexes were used to determine the optimal numbers of subphenotypes. We used 25 clustering index from Nbclust [2] to choose the number of clusters. The optimal number of clusters for each clustering index was shown in Table S5. Among all indices in development cohort:

- 6 indices proposed 2 as the best number of clusters;
- 3 indices proposed 3 as the best number of clusters;
- 11 indices proposed 4 as the best number of clusters;
- 3 indices proposed 5 as the best number of clusters;
- 2 indices proposed 6 as the best number of clusters.

Among all indices in NMEDW validation cohort:

- 9 indices proposed 2 as the best number of clusters;
- 3 indices proposed 3 as the best number of clusters;
- 10 indices proposed 4 as the best number of clusters;
- 1 indice proposed 5 as the best number of clusters;
- 2 indices proposed 6 as the best number of clusters.

Among all indices in eICU validation cohort:

- 3 indices proposed 2 as the best number of clusters;
- 4 indices proposed 3 as the best number of clusters;
- 12 indices proposed 4 as the best number of clusters;
- 3 indices proposed 5 as the best number of clusters;
- 3 indices proposed 6 as the best number of clusters.

Among all indices in CEDAR validation cohort:

- 0 indices proposed 2 as the best number of clusters;
- 7 indices proposed 3 as the best number of clusters;
- 14 indices proposed 4 as the best number of clusters;
- 2 indices proposed 5 as the best number of clusters;
- 2 indices proposed 6 as the best number of clusters.

According to the majority rule, the best number of clusters was set as 4 in experiment.

Appendix 7 Dynamic Time Warping (DTW) and Hierarchical Agglomerative Clustering (HAC)
Dynamic time warping (DTW) is one of most popular techniques to measure the similarity between two temporal sequences [3]. In DTW, the warping technique of one-to-many match is used to obtain the optimal match of two sequences so that the troughs and peaks with the same pattern are completely matched, and there is no left out for both temporal sequences. Once the matched sequences are obtained, the Euclidean Distance is used to compute the distance of two sequences. Due to its invariance against warping in the time axis, DTW has been widely utilized as a similarity measure in various domains such as speech recognition and provides more meaningful discrepancy measurements between two temporal sequences than other distance measures [4]. Previous clinical study in terms of COVID-19 has shown the DTW can be used to assess longitudinal changes in organ dysfunction and help clinicians or doctors to obtain important insights about pathophysiology [8].

Technically, DTW is a technique that compares sequences A and B through matching, where for every point in A it finds a best matching point in B. The distance between A and B is the sum of the distances between all matched point pairs. Mathematically, let $x^i = \{x_1^i, x_2^i, \ldots, x_{T_i}^i\}$ be the sequence of the *i-th* patient (1≤ i ≤N). T_i is the sequence length and x_k^i is its value at the *k-th* timestamp. In particular, given a sequence pair (x^i, x^j), (see Figure

S19) for every data point $x_k^i \in x^i$ $(1 \le k \le T_i)$, DTW identifies a matching point $x_{k'}^j \in x^j$ $(1 \le k' \le T_j)$ and according to some ground distance metric $G(x_k^i, x_{k'}^j)$, then the distance of the two sequence, $D(x^i, x^j) = \sum_{k,k'} G(x_k^i, x_{k'}^j)$. The matching process needs to satisfy 1) if $k = 1$, then $k' = 1$, which means the first points of the two sequences should match each other; 2) if $k = T_i$, then $k' = T_j$, which means the last points of the two sequences should match each other; 3) the matching should be monotonic, i.e., if $(x_{k_1}^i, x_{k'_1}^j)$and $(x_{k_2}^i, x_{k'_2}^j)$ are two matched pairs and $k_1 < k_2$, then $k'_1 \le k'_2$. DTW matches (x^i, x^j) by minimizing the sum of differences (measured by ground distance) between the all matched point pairs on the two sequences through dynamic programming [14]. After collecting all matched point pairs from x^i and x^j, we get the warping path $\mathcal{P}(x^i, x^j) = \{(x_k^i, x_{k'}^j)\}_{k,k'=1}^{T_i,T_j}$, and the number of entries in $\mathcal{P}(x^i, x^j)$ is its length. We can index the entries in $\mathcal{P}(x^i, x^j)$ according to the orders they appear in the path as $\mathcal{P}(x^i, x^j) = \{p_1^{ij}, p_2^{ij}, \dots, p_{L_{ij}}^{ij}\}$ with $p_1^{ij} = (x_1^i, x_1^j)$ and $p_{L_{ij}}^{ij} = (x_{T_i}^i, x_{T_j}^j)$, and L_{ij} is the path length satisfying $\max(T_i, T_j) \le L_{ij} \le T_i + T_j$.

In this study, after obtaining the SOFA trajectories of patients (see the Figure S20, the illustration of SOFA trajectory), each patient is represented as a vector of 12 SOFA scores from the first 6 hours to the last 6 hours across the 72 hours period after admission in ICU. DTW was used to evaluate the similarities between pairwise patient SOFA trajectories. Hierarchical Agglomerative Clustering (HAC) [13] is used to obtain subphenotypes based on the similarities of SOAF score trajectories derived by DTW. Compared to other clustering methods such as k-means, HAC has two advantages: (1) HAC is usually robust as it is not sensitive to data distribution and not necessary to have initialization procedure, which may cause uncertainty. (2) HAC typically generates a tree diagram that can visually demonstrate how the data points are agglomerated together in a hierarchical manner and provides a visible way to assistance the determination of optimal cluster number. In order to furtherly determine the number of optimal cluster number, one famous R package "NbClust" [2] was used in this study. During using the "NbClust", we considered 25 indices. The more details in terms of these indices were shown in Table S5.

Appendix 8 Group-Based Trajectory Modeling (GBTM)
In order to assess the sensitivity to different clustering methods, in this study, we used GBTM to re-derive subphenotypes. The GBTM is one of the most popular latent class analysis (LCA) that assigns each patient a probability of being in each subphenotype by considering maximum likelihood estimation [5]. The GBTM has been widely used for researchers to identify underlying subgroups in terms of disease courses such as coronary artery risk development in young adults [6] [7]. During using GBTM to derive subphenotypes, the optimal subphenotype number was determined by comprehensively taking Akaike information criterion (AIC) [11] and Bayesian information criterion (BIC) [12] into account.

Reference:

[1] https://www.nm.org/about-us/northwestern-medicine-newsroom/media-relations/about-our-health-system

[2] Charrad, Malika, Nadia Ghazzali, Véronique Boiteau, and Azam Niknafs. "NbClust: an R package for determining the relevant number of clusters in a data set." Journal of statistical software 61, no. 1 (2014): 1-36.

[3] Müller, Meinard. "Dynamic time warping." Information retrieval for music and motion (2007): 69-84.

[4] Berndt, Donald J., and James Clifford. "Using dynamic time warping to find patterns in time series." In KDD workshop, vol. 10, no. 16, pp. 359-370. 1994.

[5] Mori, Makoto, Harlan M. Krumholz, and Heather G. Allore. "Using latent class analysis to identify hidden clinical phenotypes." Jama 324, no. 7 (2020): 700-701.

[6] Patel, Ravi B., Laura A. Colangelo, Jared P. Reis, Joao AC Lima, Sanjiv J. Shah, and Donald M. Lloyd-Jones. "Association of longitudinal trajectory of albuminuria in young adulthood with myocardial structure and function in later life: Coronary Artery Risk Development in Young Adults (CARDIA) study." JAMA cardiology 5, no. 2 (2020): 184-192.

[7] Nagin, Daniel S., and Candice L. Odgers. "Group-based trajectory modeling in clinical research." Annual review of clinical psychology 6 (2010): 109-138.

[8] Su, Chang, Zhenxing Xu, Katherine Hoffman, Parag Goyal, Monika M. Safford, Jerry Lee, Sergio Alvarez-Mulett et al. "Identifying organ dysfunction trajectory-based subphenotypes in critically ill patients with COVID-19." Scientific Reports 11, no. 1 (2021): 1-13.

[9] Pollard, Tom J., Alistair EW Johnson, Jesse D. Raffa, Leo A. Celi, Roger G. Mark, and Omar Badawi. "The eICU Collaborative Research Database, a freely available multi-center database for critical care research." Scientific data 5, no. 1 (2018): 1-13.

[10] Schenck, Edward J., Katherine L. Hoffman, Marika Cusick, Joseph Kabariti, Evan T. Sholle, and Thomas R. Campion Jr. "Critical carE Database for Advanced Research (CEDAR): An automated method to support intensive care units with electronic health record data." Journal of Biomedical Informatics 118 (2021): 103789.

[11] Bozdogan H. Model selection and Akaike's information criterion (AIC): The general theory and its analytical extensions. Psychometrika 1987; 52: 345-370

[12] Vrieze SI. Model selection and psychological theory: a discussion of the differences between the Akaike information criterion (AIC) and the Bayesian information criterion (BIC). Psychological methods 2012; 17: 228.

[13] Jain AK, Murty MN, Flynn PJ. Data clustering: a review. ACM Computing Surveys 1999; 31: 264–323.

[14] Bellman, Richard. "Dynamic programming." Science 153, no. 3731 (1966): 34-37.

[15] Seymour, Christopher W., Jason N. Kennedy, Shu Wang, Chung-Chou H. Chang, Corrine F. Elliott, Zhongying Xu, Scott Berry et al. "Derivation, validation, and potential treatment implications of novel clinical phenotypes for sepsis." Jama 321, no. 20 (2019): 2003-2017.

[16] Johnson, Alistair EW, Jerome Aboab, Jesse D. Raffa, Tom J. Pollard, Rodrigo O. Deliberato, Leo Anthony Celi, and David J. Stone. "A comparative analysis of sepsis identification methods in an electronic database." Critical care medicine 46, no. 4 (2018): 494.

[17] Rhee, Chanu, Zilu Zhang, Sameer S. Kadri, David J. Murphy, Greg S. Martin, Elizabeth Overton, Christopher W. Seymour et al. "Sepsis surveillance using adult sepsis events simplified eSOFA criteria versus sepsis-3 SOFA criteria." Critical care medicine 47, no. 3 (2019): 307.

[18] Singer, Mervyn, Clifford S. Deutschman, Christopher Warren Seymour, Manu Shankar-Hari, Djillali Annane, Michael Bauer, Rinaldo Bellomo et al. "The third international consensus definitions for sepsis and septic shock (Sepsis-3)." Jama 315, no. 8 (2016): 801-810.

Table S1. Patient characteristics comparisons between survivors and nonsurvivors in the development cohort

Characteristics	Total N=4,678	Survivors N=4,170	Nonsurvivors N=508	p-value†
**Age, median (IQR)**	65.9 [53.7-77.9]	65.2 [53.2-77.4]	71.5 [59.9-80.9]	<0.001
**Sex, No. (%)**				0.418
Male	2625 (56.1%)	2349 (56.3%)	276 (54.3%)	
Female	2053 (43.9%)	1821 (43.7%)	232 (45.7%)	
**Race, No. (%)**				<0.001
WHITE	3367 (71.9%)	3022 (72.5%)	345 (67.9%)	
BLACK	424 (9.1%)	396 (9.5%)	28 (5.5%)	
OTHER	887 (18.9%)	752 (18.0%)	135 (26.6%)	
**Elixhauser index, median (IQR)**	4.0 [0.0-9.0]	4.0 [0.0-8.0]	7.0 [2.0-12.0]	<0.001
**Length stay, median (IQR)**	2.8 [1.6-5.6]	2.7 [1.6-5.2]	3.9 [1.9-7.7]	<0.001
**Mechanical ventilation at admission, No. (%)**	1893 (40.5%)	1589 (38.1%)	304 (59.8%)	<0.001
**Baseline SOFA, mean (SD)**	4.96 (2.8)	4.7 (2.6)	7.1 (3.7)	<0.001
**ICU unit at admission, No. (%)**				<0.001
CCU	443 (9.5%)	377 (9.0%)	66 (12.9%)	
CSRU	260 (5.6%)	253 (6.1%)	7 (1.4%)	
MICU	2611 (55.8%)	2293 (54.9%)	318 (62.6%)	
TSICU	593 (12.7%)	547 (13.1%)	46 (9.1%)	
SICU	771 (16.4%)	700 (16.7%)	71 (13.9%)	
**Admission location, No. (%)**				<0.001
Transfer from other hospital	4 (0.1%)	3 (0.1%)	1 (0.2%)	
Emergency room	1497 (32.0%)	1335 (32.0%)	162 (31.8%)	
Clinic referral	1985 (42.4%)	1779 (42.6%)	206 (40.5%)	
Transfer from ward	810 (17.3%)	686 (16.4%)	124 (24.4%)	
Physician referral	367 (7.8%)	355 (8.5%)	12 (2.3%)	
Transfer from skilled nursing facility	15 (0.3%)	12 (0.2%)	3 (0.5%)	
**Infection item**				
Central nervous system	56 (1.2%)	54 (1.2%)	2 (0.3%)	0.122
Intra-abdominal	880 (18.8%)	794 (19.0%)	86 (16.9%)	0.276
Pneumonia	1257 (26.8%)	1108 (26.5%)	149 (29.3%)	0.203
Septicemia bacteremia	1587 (33.9%)	1331 (31.9%)	256 (50.3%)	<0.001
Skin soft tissue	276 (5.9%)	263 (6.3%)	13 (2.5%)	<0.001
Urinary tract	1044 (22.3%)	974 (23.3%)	70 (13.7%)	<0.001

Definition of abbreviations: IQR--interquartile range; SD--standard deviation; SOFA--Sequential Organ Failure Assessment; SICU--Surgical ICU; CCU-- Coronary Care Unit; TSICU--Thoracic Surgery ICU; MICU--Medical ICU; CSRU--Cardiac Surgery ICU.
†p-value calculated by Chi-square test/Fisher's exact test, or student's t-test/Mann-Whitney test where appropriate.

Table S2. Patient characteristics comparisons between survivors and nonsurvivors in the NMEDW validation cohort

Characteristics	Total N=3,665	Survivors N=3,107	Nonsurvivors N=558	p-value†
**Age, median (IQR)**	65.0(54.0-76.0)	65.0(54.0-75.0)	69.0(59.0-78.0)	<0.001
**Sex, No. (%)**				0.877
Male	2,011(54.8%)	1,707(54.9%)	304(54.4%)	
Female	1,654(45.1%)	1,400(45.0%)	254(45.5%)	
**Race, No. (%)**				0.254
WHITE	2,596(70.8%)	2,202(70.8%)	394(70.6%)	
BLACK	526(14.3%)	455(14.6%)	71(12.7%)	
OTHER	543(14.8%)	450(14.4%)	93(16.6%)	
**Length stay, median (IQR)**	3.8(1.9-7.9)	3.7(1.9-7.6)	5.0(2.6-9.6)	<0.001
**Mechanical ventilation, No. (%)**	1,524(41.5%)	1,197(38.5%)	327(58.6%)	<0.001
**Elixhauser index, median (IQR)**	6.0(0.0-14.0)	6.0(0.0-14.0)	9.0(0.0-18.0)	<0.001
**Baseline SOFA, mean (SD)**	5.6(2.8)	5.4(2.6)	7.7(4.0)	<0.001
**Infection item**				
Central nervous system	47(1.2%)	41(1.3%)	6(1.0%)	0.789
Intra-Abdominal	383(10.4%)	321(10.3%)	62(11.1%)	0.632
Pneumonia	980(26.7%)	832(26.7%)	148(26.5%)	0.942
Septicemia bacteremia	1293(35.2%)	1034(33.2%)	259(46.4%)	<0.001
Skin soft tissue	157(4.2%)	146(4.7%)	11(1.9%)	0.005
Urinary tract	410(11.1%)	367(11.8%)	43(7.7%)	0.006

Definition of abbreviations: IQR--interquartile range; SD--standard deviation; SOFA--Sequential Organ Failure Assessment.
†p-value calculated by Chi-square test/Fisher's exact test, or student's t-test/Mann-Whitney test where appropriate.

Table S3. Patient characteristics comparisons between survivors and nonsurvivors in the eICU validation cohort

Characteristics	Total N=12,282	Survivors N=10,995	Nonsurvivors N=1,287	p-value†
**Age, median (IQR)**	67.0 [56.0-78.0]	67.0 [55.0-78.0]	71.0 [60.5-81.0]	<0.001
**Sex, No. (%)**				0.517
Male	6438 (52.4%)	5782 (52.5%)	656 (50.9%)	
Female	5844 (47.5%)	5213 (47.4%)	631 (49.0%)	
**Ethnicity, No. (%)**				0.571
Caucasian	9434 (76.8%)	8428 (76.6%)	1006 (78.1%)	
African American	1317 (10.7%)	1198 (10.9%)	119 (9.2%)	
Hispanic	593 (4.8%)	528 (4.8%)	65 (5.0%)	
Native American	111 (0.9%)	99 (0.9%)	12 (0.9%)	
Asian	201 (1.6%)	178 (1.6%)	23 (1.7%)	
Other/Unknown	626 (5.1%)	564 (5.1%)	62 (4.8%)	
**Elixhauser index, median (IQR)**	5.0 [0.0-12.0]	3.0 [0.0-12.0]	7.0 [0.0-18.0]	<0.001
**Length stay, median (IQR)**	2.8 [1.7-5.1]	2.8 [1.7-4.9]	3.18 [1.6-6.8]	0.002
**Mechanical ventilation at admission, No. (%)**	5329 (43.3%)	4587 (41.7%)	742 (57.6%)	<0.001
**Baseline SOFA, mean (SD)**	5.9 (3.0)	5.66 (2.8)	8.72 (3.7)	<0.001
**ICU unit at admission, No. (%)**				0.042
SICU	627 (5.1%)	558 (5.0%)	69 (5.3%)	
MICU	1575 (12.8%)	1402 (12.7%)	173 (13.4%)	
CTICU	87 (0.7%)	82 (0.7%)	5 (0.3%)	
CSICU	160 (1.3%)	138 (1.2%)	22 (1.7%)	
MSICU	7797 (63.4%)	6986 (63.5%)	811 (63.0%)	
CCU-CTICU	772 (6.2%)	684 (6.2%)	88 (6.8%)	
CICU	907 (7.3%)	808 (7.3%)	99 (7.6%)	
Neuro-ICU	357 (2.9%)	337 (3.0%)	20 (1.5%)	
**Admission location, No. (%)**				<0.001
Floor	1986 (16.1%)	1738 (15.8%)	248 (19.2%)	
SDU	218 (1.7%)	176 (1.6%)	42 (3.2%)	
Direct Admit	852 (6.9%)	760 (6.9%)	92 (7.1%)	
Other Hospital	404 (3.2%)	345 (3.1) %	59 (4.5) %	
ICU	4 (0.03%)	4 (0.04%)	0 (0.0%)	
Other ICU	63 (0.5%)	51 (0.4%)	12 (0.9%)	
ICU to SDU	1 (0.01%)	1 (0.01%)	0 (0.0%)	
PACU	17 (0.14%)	16 (0.15%)	1 (0.08%)	
Recovery Room	146 (1.1%)	138 (1.2%)	8 (0.6%)	
Chest Pain Center	5 (0.04%)	5 (0.05%)	0 (0.00%)	
Emergency Department	7825 (63.7%)	7073 (64.3%)	752 (58.4%)	
Acute Care/Floor	284 (2.3%)	247 (2.2%)	37 (2.8%)	
Operating Room	477 (3.8%)	441 (4.0%)	36 (2.8%)	
**Infection item, No. (%)**				
Central nervous system	63 (0.5%)	58 (0.5%)	5 (0.3%)	0.649
Intra-abdominal	790 (6.4%)	689 (6.2%)	101 (7.8%)	0.033

Pneumonia	4743 (38.6%)	4176 (37.9%)	567 (44.0%)	<0.001
Septicemia bacteremia	7350 (59.8%)	6460 (58.7%)	890 (69.1%)	<0.001
Skin soft tissue	632 (5.1%)	590 (5.3%)	42 (3.2%)	<0.001
Urinary tract	2478 (20.1%)	2279 (20.7%)	199 (15.4%)	<0.001

Definition of abbreviations: IQR--interquartile range; SD--standard deviation; SOFA--Sequential Organ Failure Assessment; SICU-- Surgical ICU; MICU--Medical ICU; CTICU--Cardiothoracic ICU; CSICU--Cardiac Surgery ICU; MSICU--Medical-Surgical ICU; CCU--Coronary Care Unit; CICU-- Cardiac ICU; Neuro-ICU-- Neurological ICU; SDU--Step-Down Unit.

† p-value calculated by Chi-square test/Fisher's exact test, or student's t-test/Mann-Whitney test where appropriate.

Table S4. Patient characteristics comparisons between survivors and nonsurvivors in the CEDAR validation cohort

Characteristics	Total<br>N=4,804	Survivors<br>N=3,850	Nonsurvivors<br>N=954	p-value†
**Age, median (IQR)**	77.0 [66.0-88.0]	77.0 [65.0-88.0]	78.0 [68.0-89.0]	0.004
**Sex, No. (%)**				0.133
Male	2320 (48.2%)	1838 (47.7%)	482 (50.5%)	
Female	2484 (51.7%)	2012 (52.2%)	472 (49.4%)	
**Race, No. (%)**				0.238
WHITE	1791 (37.2%)	1420 (36.8%)	371 (38.8%)	
BLACK	446 (9.2%)	350 (9.0%)	96 (10.0%)	
OTHER	2567 (53.4%)	2080 (54.0%)	487 (51.0%)	
**Length stay, median (IQR)**	4.42(2.6-7.9)	4.2(2.6-7.7)	5.0(2.5-9.4)	0.026
**Mechanical ventilation, No. (%)**	2263(47.1%)	1624(42.1%)	639(66.9%)	<0.001
**Elixhauser index, median (IQR)**	12.5 [0.0-27.0]	11.0 [0.0-25.0]	21.0 [0.0-33.0]	<0.001
**Baseline SOFA, mean (SD)**	6.38 (3.1)	5.8 (2.8)	8.43 (3.40)	<0.001
**ICU unit at admission, No. (%)**				<0.001
CTICU	656 (13.6%)	627 (16.2%)	29 (3.0%)	
SICU	752 (15.6%)	658 (17.0%)	94 (9.8%)	
MICU	2641 (54.9%)	1976 (51.3%)	665 (69.7%)	
CICU	755 (15.7%)	589 (15.3%)	166 (17.4%)	
**Infection item**				
Central nervous system	38 (0.7%)	30 (0.7%)	8 (0.8%)	0.985
Intra-Abdominal	499 (10.3%)	375 (9.7%)	124 (13.0%)	0.004
Pneumonia	597 (12.4%)	431 (11.1%)	166 (17.4%)	<0.001
Septicemia bacteremia	1376 (28.6%)	1001 (26.0%)	375 (39.3%)	<0.001
Skin soft tissue	164 (3.4%)	137 (3.5%)	27 (2.8%)	0.312
Urinary tract	1127 (23.4%)	977 (25.3%)	150 (15.7%)	<0.001

Definition of abbreviations: IQR--interquartile range; SD--standard deviation; SOFA--Sequential Organ Failure Assessment; CTICU--Cardiothoracic ICU; SICU-- Surgical ICU; MICU-- Medical ICU; CICU-- Cardiac ICU.

†p-value calculated by Chi-square test/Fisher's exact test, or student's t-test/Mann-Whitney test where appropriate.

Table S5. Multiple clustering metrics statistics and cluster number determination

Clustering Metrics	Optimal Cluster Number in Development Cohort	Optimal Cluster Number in the NMEDW Validation Cohort	Optimal Cluster Number in the eICU Validation Cohort	Optimal Cluster Number in the CEDAR Validation Cohort
**KL**	4	4	4	4
**CH**	2	2	4	4
**Hartigan**	6	4	6	4
**CCC**	2	2	2	3
**Scott**	4	4	4	4
**Marriot**	4	4	4	4
**TrCovW**	4	4	4	4
**TraceW**	4	4	4	4
**Friedman**	4	4	4	4
**Rubin**	4	4	4	4
**Cindex**	5	6	5	4
**DB**	5	3	3	3
**Silhouette**	2	2	2	3
**Duda**	2	2	5	5
**PseudoT2**	3	3	5	5
**Beale**	2	2	3	3
**Ratkowsky**	6	2	4	4
**Ball**	3	3	4	4
**PtBiserial**	4	4	3	3
**Gap**	2	2	4	4
**Frey**	4	2	4	4
**McClain**	4	6	3	3
**Dunn**	4	4	6	6
**SDindex**	3	2	2	3
**SDbw**	5	5	6	6
**Total**	# of 2: 6<br># of 3: 3<br>**# of 4: 11**<br># of 5: 3<br># of 6: 2	# of 2: 9<br># of 3: 3<br>**# of 4: 10**<br># of 5: 1<br># of 6: 2	# of 2: 3<br># of 3: 4<br>**# of 4: 12**<br># of 5: 3<br># of 6: 3	# of 2: 0<br># of 3: 7<br>**# of 4: 14**<br># of 5: 2<br># of 6: 2

Definition of abbreviations: KL-- Krzanowski-Lai index; CH-- Calinski-Harabasz index; CCC-- Cubic Clustering Criterion, DB-- Davies–Bouldin index; All those clustering metrics are from NbClust Package.

Table S6. Patient physiological characteristics comparisons among subphenotypes in the development cohort

Characteristics	Total (N=4,678)	DI (N=1,174)	RI (N=1,932)	DW (N=960)	RW (N=612)	p-value†	Post-hoc
Albumin, g/dL, median [IQR]	2.9 [2.5-3.3]	2.9 [2.5-3.32]	3.0 [2.5-3.3]	2.9 [2.5-3.3]	2.9 [2.4-3.3]	0.346	
ALT, IU/L, median [IQR]	35.0 [19.0-86.0]	33.0 [19.0-85.75]	36.5 [20.0-89.25]	30.0 [18.0-71.0]	40.0 [21.0-86.25]	0.016	DW vs RI, RW
AST, IU/L, median [IQR]	49.0 [27.0-120.0]	48.0 [28.0-125.0]	49.0 [27.0-115.2]	45.0 [24.0-106.0]	61.0 [32.5-143.5]	<0.001	DI vs DW, RW; RW vs RI, DW
Bands, median [IQR]	2.0 [0.0-8.0]	2.0 [0.0-9.0]	2.0 [0.0-9.0]	2.0 [0.0-8.0]	2.0 [0.0-8.0]	0.712	
Bicarbonate, mEq/L, median [IQR]	23.0 [20.0-26.0]	24.0 [21.0-26.0]	23.0 [20.0-25.0]	23.0 [19.0-26.0]	22.0 [19.0-26.0]	<0.001	DI vs RI, DW, RW
Bilirubin, mg/dL, median [IQR]	0.8 [0.4-2.1]	1.0 [0.5-2.4]	0.8 [0.4-2.0]	0.7 [0.4-1.6]	1.1 [0.5-2.5]	<0.001	DI vs RI, DW; RI vs DW; RW vs RI, DW
BMI, kg/m2, median [IQR]	27.5 [23.7-32.2]	27.4 [23.7-32.4]	27.6 [23.9-32.5]	27.9 [23.9-32.4]	26.8 [23.5-30.8]	0.227	
BUN, mg/dL, median [IQR]	22.0 [15.0-38.0]	20.0 [14.0-32.0]	22.0 [15.0-37.0]	27.0 [16.0-47.0]	23.0 [15.0-38.0]	<0.001	DI vs RI, DW, RW; RI vs DW; DW vs RW
Chloride, mEq/L, median [IQR]	106.0 [102.0-110.0]	106.0 [101.0-109.0]	106.0 [102.0-110.0]	106.0 [102.0-110.0]	106.0 [101.0-110.0]	0.014	DI vs RI; RI vs RW
Creatinine, mg/dL, median [IQR]	1.1 [0.8-1.7]	1.0 [0.7-1.4]	1.1 [0.8-1.7]	1.3 [0.9-2.4]	1.1 [0.8-1.7]	<0.001	DI vs RI, DW, RW; RI vs DW; DW vs RW
CRP, mg/dL, median [IQR]	92.5 [40.5-174.4]	107.0 [51.4-181.6]	81.5 [42.2-172.1]	82.6 [29.25-177.15]	72.5 [41.7-143.7]	0.825	
GCS, median [IQR]	13.0 [7.0-15.0]	13.0 [7.0-15.0]	13.0 [7.0-15.0]	14.0 [7.0-15.0]	12.0 [6.0-15.0]	0.203	
Glucose, mg/dL, median [IQR]	131.0 [106.0-172.0]	130.0 [106.0-167.75]	132.0 [107.0-173.0]	131.0 [106.0-174.0]	134.0 [105.0-176.0]	0.746	
Heart rate, beats/min, median [IQR]	95.0 [81.0-110.0]	96.0 [82.0-110.0]	93.42 [80.5-108.3]	94.12 [80.0-110.2]	96.83 [84.0-112.0]	0.002	DI vs RI, RI vs RW, DW vs RW
Hemoglobin, g/dL	10.6 [9.2-12.0]	10.57 [9.3-11.9]	10.7 [9.3-12.1]	10.3 [9.1-11.8]	10.6 [9.07-12.1]	0.041	RI vs DW
INR, median [IQR]	1.3 [1.2-1.6]	1.3 [1.2-1.6]	1.3 [1.1-1.5]	1.3 [1.2-1.6]	1.4 [1.2-1.7]	<0.001**	DI, RI, DW vs RW
Lactate, mmol/L, median [IQR]	1.8 [1.2-2.8]	1.8 [1.2-2.8]	1.7 [1.2-2.7]	1.7 [1.2-2.7]	2.0 [1.3-3.3]	<0.001§	DI, RI, DW vs RW
lymphocyte count, median [IQR]	940.0 [675.0-1570.0]	1278.0 [775.0-1854.5]	805.0 [539.2-1172.0]	1033.5 [866.2-1792.5]	780.0 [460.0-1690.0]	0.262	
lymphocyte percentage, %, median [IQR]	7.1 [3.5-12.5]	7.5 [4.0-12.6]	7.2 [3.5-12.4]	6.8 [3.3-12.0]	6.75 [3.5-13.5]	0.283	
Pao2, mm Hg	108.0 [81.5-168.5]	101.5 [86.5-109.5]	110.0 [70.0-157.0]	102.5 [83.7-162.7]	170.0 [90.0-213.5]	0.364	
Platelet, x10(9)/L, median [IQR]	190.0 [130.0-260.0]	186.0 [125.0-258.2]	199.0 [138.75-268.0]	190.0 [132.0-252.7]	168.0 [112.75-237.0]	<0.001	DI vs RI, DW; RI vs DW, RW; DW vs RW
RDW, %, median [IQR]	14.8 [13.8-16.3]	14.8 [13.8-16.2]	14.6 [13.6-16.0]	15.0 [13.9-16.6]	14.9 [13.8-16.8]	<0.001	DI vs RI; RI vs DW, RW
Respiratory rate, breaths/min, median [IQR]	22.0 [19.0-27.0]	23.0 [19.0-27.0]	22.0 [19.0-26.0]	22.0 [19.0-26.75]	22.33 [19.0-27.0]	0.023	DI vs RI; RI vs RW
SO2, %, median [IQR]	94.0 [80.0-97.0]	94.0 [83.0-97.0]	93.0 [78.0-97.0]	95.0 [78.38-97.25]	94.0 [82.0-98.0]	0.014	DI vs RI

**Sodium, mmol/L, median [IQR]**	139.0 [136.0-142.0]	139.0 [136.0-141.0]	139.0 [136.0-141.0]	139.0 [136.0-142.0]	139.0 [136.0-142.0]	0.973	
**Systolic ABP, mm Hg, median [IQR]**	101.0 [91.0-115.0]	102.0 [92.0-118.0]	100.0 [91.0-112.0]	102.0 [91.7-115.0]	102.0 [91.0-117.0]	<0.001	DI vs RI; RI vs DW, RW;
**Temperature, degrees C, median [IQR]**	36.8 [36.3-37.4]	36.8 [36.3-37.4]	36.8 [36.3-37.5]	36.8 [36.2-37.4]	36.78 [36.2-37.3]	0.030	RI vs DW, RW
**Troponin T, ng/mL, median [IQR]**	0.1 [0.0-0.3]	0.1 [0.0-0.3]	0.1 [0.0-0.3]	0.1 [0.0-0.2]	0.1 [0.0-0.3]	0.404	
**WBC, x10(9)/L, median [IQR]**	11.1 [7.5-15.8]	10.6 [7.1-14.9]	11.4 [7.9-16.4]	10.9 [7.4-15.3]	11.0 [7.3-16.2]	<0.001	DI vs RI, RI vs DW

Definition of abbreviations: IQR--interquartile range; SD--standard deviation; ALT-- alanine aminotransferase; AST-- alanine aminotransferase; BMI-- body mass index, BUN-- blood urea nitrogen; CRP-- C-reactive protein; GCS-- Glasgow coma scale score; INR-- international normalized ratio; RDW-- red blood cell distribution width; ABP-- Ambulatory Blood Pressure; WBC--white blood cell count; DI-- Delayed Improving; RI-- Rapidly Improving; DW-- Delayed Worsening; RW-- Rapidly Worsening.

† p-value calculated by analysis of variance (ANOVA)/Kruskal–Wallis test.

** False discovery rate corrected p-value < 0.001

§ Age adjusted (analysis of covariance [ANCOVA]) p-value < 0.001

Table S7. Patient basic characteristics comparisons among subphenotypes in the NMEDW validation cohort

Characteristics	Total<br>N=3665	DI<br>N=668	RI<br>N=2,019	DW<br>N=302	RW<br>N=676	p-value†
**Age, median (IQR)**	65.0(54.0-76.0)	66.0(55.0-77.0)	66.0(55.0-76.0)	66.0(54.2-76.7)	64.0(52.0-74.0)	0.027
**Sex, No. (%)**						0.463
Male	2011(54.8%)	366(54.7%)	1116(55.2%)	174(57.6%)	355(52.5%)	
Female	1654(45.1%)	302(45.2%)	903(44.7%)	128(42.3%)	321(47.4%)	
**Race, No. (%)**						<0.001
WHITE	2596(70.8%)	472(70.6%)	1481(73.3%)	205(67.8%)	438(64.7%)	
BLACK	526(14.3%)	93(13.9%)	281(13.9%)	42(13.9%)	110(16.2%)	
OTHER	543(14.8%)	103(15.4%)	257(12.7%)	55(18.2%)	128(18.9%)	
**Length stay, median (IQR)**	3.8(1.9-7.9)	4.4(2.0-9.1)	3.2(1.8-6.1)	4.2(2.1-9.2)	5.44(2.3-11.5)	<0.001
**Mechanical ventilation at admission, No. (%)**	1524(41.5%)	252(37.7%)	882(43.6%)	111(36.7%)	279(41.2%)	0.014
**Elixhauser index, median (IQR)**	6.0(0.0-14.0)	7.00(0.0-15.0)	5.0(0.0-12.0)	8.0(0.0-17.0)	9.00(0.0-17.0)	<0.001
**Baseline SOFA, mean (SD)**	5.6(2.8)	4.71(2.3)	6.1(2.9)	6.0(2.8)	5.22(2.5)	<0.001
**Infection item, No. (%)**						
Central nervous system	47(1.2%)	11(1.6%)	18(0.8%)	7(2.3%)	11(1.6%)	0.097
Intra-Abdominal	383(10.4%)	68(10.1%)	198(9.8%)	36(11.9%)	81(11.9%)	0.342
Pneumonia	980(26.7%)	207(30.9%)	478(23.6%)	81(26.8%)	214(31.6%)	<0.001
Septicemia bacteremia	1293(35.2%)	227(33.9%)	667(33.0%)	124(41.0%)	275(40.6%)	<0.001
Skin soft tissue	157(4.2%)	24(3.5%)	93(4.6%)	16(5.3%)	24(3.5%)	0.402
Urinary tract	410(11.1%)	91(13.6%)	224(11.0%)	37(12.2%)	58(8.5%)	0.030
**Septic shock, No. (%)**	366(9.9%)	54(8.0%)	229(11.3%)	36(11.9%)	47(6.9%)	0.002

Definition of abbreviations: IQR--interquartile range; SD--standard deviation; SOFA--Sequential Organ Failure Assessment; DI-- Delayed Improving; RI--Rapidly Improving; DW-- Delayed Worsening; RW-- Rapidly Worsening.

†p-value calculated by Chi-square test/Fisher's exact test, or student's t-test/Mann-Whitney test where appropriate.

Table S8. Patient physiological characteristics comparisons among subphenotypes in the NMEDW validation cohort

Characteristics	Total N=3665	DI N=668	RI N=2,019	DW N=302	RW N=676	p-value†	Post-hoc
**Albumin, g/dL, median [IQR]**	2.9(2.4-3.4)	3.0(2.4-3.4)	3.0(2.4-3.5)	2.8(2.4-3.4)	2.9(2.4-3.4)	0.174	
**ALT, IU/L, median [IQR]**	26.0(15.0-58.0)	27.0(16.0-55.0)	25.0(15.0-54.0)	24.5(16.0-51.2)	30.0(16.2-73.5)	0.061	
**AST, IU/L, median [IQR]**	36.0(21.0-81.0)	38.0(21.2-79.0)	34.0(21.0-72.0)	31.5(19.0-65.0)	42.0(24.0-100.0)	0.003	RW vs RI, DW
**Bands, median [IQR]**	6.0(2.0-12.0)	5.0(2.0-7.0)	8.0(3.0-13.2)	7.0(2.0-8.0)	6.0(2.0-18.0)	0.256	
**Bicarbonate, mEq/L, median [IQR]**	22.0(19.0-26.0)	23.0(19.0-26.0)	22.0(19.0-25.0)	22.0(17.0-25.0)	22.0(18.0-26.0)	0.336	
**Bilirubin, mg/dL, median [IQR]**	0.8(0.5-1.7)	0.9(0.5-1.9)	0.8(0.5-1.6)	0.8(0.5-1.4)	0.9(0.5-1.9)	0.056	
**BMI, kg/m2, median [IQR]**	27.6(23.2-33.2)	27.2(23.4-32.1)	27.8(23.3-33.5)	27.6(22.9-32.4)	27.6(23.1-33.2)	0.481	
**BUN, mg/dL, median [IQR]**	22.0(13.0-40.0)	22.0(13.0-41.0)	22.0(13.0-40.0)	22.5(13.7-37.2)	23.0(14.0-39.0)	0.935	
**Chloride, mEq/L, median [IQR]**	104.0(100.0-109.0)	104.0(100.0-108.0)	105.0(100.0-109.0)	105.0(100.5-109.0)	104.0(100.0-109.0)	0.218	
**Creatinine, mg/dL, median [IQR]**	1.2(0.8-2.1)	1.1(0.7-2.1)	1.25(0.8-2.1)	1.2(0.8-2.0)	1.2(0.8-1.9)	0.350	
**CRP, mg/dL, median [IQR]**	14.3(6.2-28.7)	14.3(6.1-24.9)	15.1(5.1-36.1)	13.6(8.0-22.9)	12.7(6.6-26.8)	0.974	
**GCS, median [IQR]**	13.0(8.0-15.0)	15.0(10.0-15.0)	10.0(7.0-15.0)	11.0(7.0-15.0)	14.0(10.0-15.0)	<0.001	DI vs RI, DW; RI vs DW, RW; DW vs RW
**Glucose, mg/dL, median [IQR]**	144.0(112.5-196.0)	138.0(111.5-185.5)	145.0(114.0-194.0)	149.0(117.0-215.0)	146.0(110.0-200.0)	0.364	
**Heart rate, beats/min, median [IQR]**	96.0(82.0-111.0)	96.0(80.0-113.0)	96.0(82.2-110.0)	97.5(78.0-111.7)	100.0(85.0-115.0)	0.179	
**Hemoglobin, g/dL**	10.0(8.3-11.9)	9.9(8.3-11.8)	10.2(8.5-12.0)	9.7(8.0-11.8)	9.6(8.0-11.9)	0.109	
**INR, median [IQR]**	1.3(1.2-1.7)	1.4(1.2-1.7)	1.3(1.2-1.6)	1.4(1.2-1.6)	1.4(1.1-1.8)	0.427	
**Lactate, mmol/L, median [IQR]**	15.6(9.4-37.1)	14.6(9.1-31.5)	19.1(9.6-34.9)	20.7(8.5-51.2)	19.3(11.1-62.1)	0.810	
**lymphocyte count, median [IQR]**	900.0(500.0-1500.0)	865.0(500.0-1600.0)	1000.0(500.0-1500.0)	800.0(400.0-1300.0)	870.0(400.0-1600.0)	0.177	
**lymphocyte percentage, %, median [IQR]**	10.0(5.0-18.0)	9.0(5.0-20.0)	10.0(5.0-17.0)	8.0(5.0-15.0)	10.0(5.0-18.0)	0.602	
**Pao2, mm Hg**	99.0(73.0-146.0)	95.0(71.1-148.5)	100.0(74.0-142.0)	93.5(71.8-136.5)	103.0(73.0-152.7)	0.697	
**Platelet, x10(9)/L, median [IQR]**	176.0(107.0-254.0)	163.0(100.5-241.0)	187.0(113.0-262.0)	186.0(121.0-270.5)	162.0(93.0-236.0)	<0.001**	DI vs RI DW; RW vs RI, DW
**RDW, %, median [IQR]**	15.5(14.1-17.6)	15.5(14.1-17.8)	15.3(14.0-17.4)	15.7(14.1-17.6)	15.9(14.5-17.9)	<0.001	RI vs RW

**Respiratory rate, breaths/min, median [IQR]**	25.0(20.0-32.0)	26.0(20.0-32.0)	24.00(20.0-31.0)	26.0(20.0-32.0)	26.0(20.0-34.0)	<0.001[§]	DI vs RI; RI vs RW
**SO2, %, median [IQR]**	93.0(90.0-96.0)	93.0(90.0-96.0)	94.0(90.0-96.0)	94.0(90.0-96.0)	93.0(90.0-96.0)	0.072	
**Sodium, mmol/L, median [IQR]**	137.0(134.0-140.0)	137.0(133.0-140.0)	137.0(134.0-141.0)	137.0(133.5-140.0)	137.0(134.0-140.0)	0.170	
**Systolic ABP, mm Hg, median [IQR]**	93.0(79.0-110.0)	98.0(82.0-117.0)	91.0(78.0-108.0)	92.0(77.0-106.0)	95.0(81.0-113.0)	<0.001	DI vs RI, DW, RW; RI vs RW; DW vs RW
**Temperature, degrees C, median [IQR]**	37.1(36.7-37.9)	37.1(36.7-37.9)	37.1(36.7-37.8)	37.1(36.6-37.8)	37.1(36.6-37.9)	0.822	
**Troponin I, ng/mL, median [IQR]**	0.04(0.02-0.14)	0.04(0.02-0.16)	0.04(0.02-0.11)	0.05(0.02-0.15)	0.04(0.02-0.15)	0.381	
**WBC, x10(9)/L, median [IQR]**	12.0(8.0-17.3)	11.8(7.8-16.6)	12.4(8.3-17.3)	11.4(8.0-18.1)	11.5(6.9-17.1)	<0.001	RI vs RW

Definition of abbreviations: IQR--interquartile range; SD--standard deviation; ALT-- alanine aminotransferase; AST-- alanine aminotransferase; BMI-- body mass index, BUN-- blood urea nitrogen; CRP-- C-reactive protein; GCS-- Glasgow coma scale score; RDW-- red blood cell distribution width; WBC--white blood cell count; DI-- Delayed Improving; RI--Rapidly Improving; DW-- Delayed Worsening; RW-- Rapidly Worsening.

† p-value calculated by analysis of variance (ANOVA)/Kruskal–Wallis test

** False discovery rate corrected p-value < 0.001

§ Age adjusted (analysis of covariance [ANCOVA]) p-value < 0.001

Table S9. Patient basic characteristics comparisons among subphenotypes in the eICU validation cohort

Characteristics	Total N=12,282	DI N=2,005	RI N=5,938	DW N=1,392	RW N=2,947	p-value†
**Age, median (IQR)**	67.0 [56.0-78.0]	68.0 [55.0-79.0]	67.0 [55.0-78.0]	68.0 [57.0-79.0]	68.0 [56.0-80.0]	0.049
**Sex, No. (%)**						0.979
Male	6438 (52.4%)	1054 (52.5%)	3116 (52.4%)	729 (52.3%)	1539 (52.2%)	
Female	5844 (47.5%)	951 (47.4%)	2822 (47.5%)	663 (47.6%)	1408 (47.7%)	
**Ethnicity, No. (%)**						0.052
Caucasian	9434 (76.8%)	1554 (77.5%)	4584 (77.2%)	1076 (77.3%)	2220 (75.3%)	
African American	1317 (10.7%)	212 (10.5%)	623 (10.4%)	165 (11.8%)	317 (10.7%)	
Hispanic	593 (4.8%)	87 (4.3%)	264 (4.4%)	63 (4.5%)	179 (6.0%)	
Native American	111 (0.9%)	13 (0.6%)	55 (0.9%)	16 (1.1%)	27 (0.9%)	
Asian	201 (1.6%)	32 (1.6%)	104 (1.7%)	14 (1.0%)	51 (1.7%)	
Other/Unknown	626 (5.1%)	107 (5.3%)	308 (5.1%)	58 (4.1%)	153 (5.1%)	
**Elixhauser index, median (IQR)**	5.0 [0.0-12.0]	5.0 [0.0-14.0]	3.0 [0.0-11.0]	7.0 [0.0-15.0]	6.0 [0.0-14.0]	<0.001
**Length stay, median (IQR)**	2.8 [1.7-5.1]	3.3 [2.1-5.7]	2.4 [1.5-4.0]	3.4 [1.9-6.0]	3.0 [1.6-6.6]	<0.001
**Baseline SOFA, mean (SD)**	5.9 (3.08)	5.4 (2.7)	6.1 (3.1)	6.3 (3.1)	5.5 (2.9)	<0.001
**Mechanical ventilation at admission, No. (%)**	5329 (43.3%)	877 (43.7%)	2590 (43.6%)	609 (43.7%)	1253 (42.5%)	0.751
**ICU unit at admission, No. (%)**						0.381
SICU	627 (5.1%)	104 (5.1%)	295 (4.9%)	90 (6.4%)	138 (4.6%)	
MICU	1575 (12.8%)	261 (13.0%)	770 (12.9%)	168 (12.0%)	376 (12.7%)	
CTICU	87 (0.7%)	15 (0.7%)	33 (0.5%)	14 (1.0%)	25 (0.8%)	
CSICU	160 (1.3%)	27 (1.3%)	72 (1.2%)	15 (1.1%)	46 (1.5%)	
MSICU	7797 (63.4%)	1267 (63.1%)	3809 (64.1%)	858 (61.6%)	1863 (63.2%)	
CCU-CTICU	772 (6.2%)	131 (6.5%)	373 (6.2%)	85 (6.1%)	183 (6.2%)	
CICU	907 (7.3%)	143 (7.1%)	410 (6.9%)	122 (8.7%)	232 (7.8%)	
Neuro ICU	357 (2.9%)	57 (2.8%)	176 (2.9%)	40 (2.8%)	84 (2.8%)	
**Admission location, No. (%)**						<0.001
ICU	4 (0.03%)	2 (0.1%)	1 (0.02%)	0 (0.0%)	1 (0.03%)	
ICU to SDU	1 (0.01%)	0 (0.0%)	0 (0.0%)	0 (0.0%)	1 (0.03%)	
Floor	1986 (16.1%)	360 (17.9%)	824 (13.8%)	225 (16.1%)	577 (19.5%)	
Other Hospital	404 (3.2%)	76 (3.7%)	180 (3.0%)	61 (4.3%)	87 (2.9%)	
Operating Room	477 (3.8%)	84 (4.1%)	264 (4.4%)	60 (4.3%)	69 (2.3%)	
PACU	17 (0.14%)	2 (0.1%)	11 (0.1%)	2 (0.1%)	2 (0.07%)	
Acute Care/Floor	284 (2.3%)	42 (2.0%)	128 (2.1%)	28 (2.0%)	86 (2.9%)	
Direct Admit	852 (6.9%)	156 (7.7%)	397 (6.6%)	106 (7.6%)	193 (6.5%)	
Other ICU	63 (0.5%)	13 (0.6%)	34 (0.5%)	5 (0.3%)	11 (0.3%)	
SDU	218 (1.7%)	35 (1.7%)	99 (1.6%)	31 (2.2%)	53 (1.8%)	
Recovery Room	146 (1.1%)	24 (1.2%)	86 (1.4%)	16 (1.1%)	20 (0.6%)	
Emergency Department	7825 (63.7%)	1210 (60.3%)	3911 (65.8%)	858 (61.6%)	1846 (62.6%)	
**Infection item, No. (%)**						
Central nervous system	63 (0.5%)	17 (0.8%)	20 (0.3%)	8 (0.5%)	18 (0.6%)	0.033
Intra-Abdominal	790 (6.4%)	139 (6.9%)	341 (5.7%)	112 (8.0%)	198 (6.7%)	0.007
Pneumonia	4743 (38.6%)	834 (41.6%)	2137 (35.9%)	545 (39.1%)	1227 (41.6%)	<0.001
Septicemia bacteremia	7350 (59.8%)	1118 (55.7%)	3582 (60.3%)	827 (59.4%)	1823 (61.8%)	<0.001
Skin soft tissue	632 (5.1%)	112 (5.5%)	304 (5.1%)	68 (4.8%)	148 (5.0%)	0.779
Urinary tract	2478 (20.1%)	388 (19.3%)	1231 (20.7%)	314 (22.5%)	545 (18.4%)	0.007
**Septic shock, No. (%)**	1649 (13.4%)	250 (12.4%)	719 (12.1%)	166 (11.9%)	514 (17.4%)	<0.001

Definition of abbreviations: IQR--interquartile range; SD--standard deviation; SOFA--Sequential Organ Failure Assessment; SICU-- Surgical ICU; MICU--Medical ICU; CTICU--Cardiothoracic ICU; CSICU--Cardiac Surgery ICU; MSICU--Medical-Surgical ICU; CCU--Coronary Care Unit; CICU-- Cardiac ICU; Neuro-ICU--Neurological ICU; SDU--Step-Down Unit; DI-- Delayed Improving; RI--Rapidly Improving; DW--Delayed Worsening; RW-- Rapidly Worsening.

†p-value calculated by Chi-square test/Fisher's exact test, or student's t-test/Mann-Whitney test where appropriate.

Table S10. Patient physiological characteristics comparisons among subphenotypes in the eICU validation cohort

Characteristics	Total N=12,282	DI N=2,005	RI N=5,938	DW N=1,392	RW N=2,947	p-value†	Post-hoc
**Albumin, g/dL, median [IQR]**	2.7 [2.1-3.3]	2.7 [2.0-3.3]	2.8 [2.2-3.4]	2.7 [2.0-3.3]	2.6 [2.0-3.2]	<0.001	RI vs RW
**ALT, IU/L, median [IQR]**	23.0 [13.0-44.7]	22.0 [11.0-42.0]	23.0 [12.0-42.0]	24.0 [13.0-47.0]	25.0 [13.0-50.0]	<0.001	RI vs DW, RW
**AST, IU/L, median [IQR]**	29.0 [17.0-59.0]	28.0 [16.0-54.0]	28.0 [16.0-54.0]	31.0 [17.0-66.0]	34.0 [18.0-74.5]	<0.001	RW vs DI, RI
**Bands, median [IQR]**	11.0 [4.0-20.0]	10.0 [5.0-19.0]	10.0 [4.0-20.0]	11.0 [5.0-19.0]	11.0 [4.0-21.0]	0.126	
**Bicarbonate, mEq/L, median [IQR]**	21.4 [17.4-25.0]	22.0 [18.0-26.0]	21.6 [18.0-25.0]	21.8 [17.0-25.05]	21.0 [16.0-24.9]	<0.001	RI vs RW
**Bilirubin, mg/dL, median [IQR]**	0.7 [0.4-1.2]	0.7 [0.4-1.2]	0.7 [0.4-1.2]	0.7 [0.4-1.3]	0.8 [0.5-1.4]	<0.001	RW vs DI, RI, DW
**BMI, kg/m2, median [IQR]**	27.1 [22.8-33.1]	26.9 [22.6-33.3]	27.5 [23.2-33.4]	26.8 [22.6-32.4]	26.6 [22.3-32.6]	<0.001	RI vs RW
**BUN, mg/dL, median [IQR]**	30.0 [19.0-49.0]	28.0 [17.0-45.0]	31.0 [19.0-50.0]	32.0 [20.0-52.0]	30.0 [19.0-48.0]	<0.001	DI vs DW
**Chloride, mEq/L, median [IQR]**	103.0 [99.0-108.0]	103.0 [99.0-108.0]	103.0 [99.0-108.0]	104.0 [99.0-109.0]	103.0 [99.0-108.0]	0.035	DI vs DW
**Creatinine, mg/dL, median [IQR]**	1.5 [0.9-2.5]	1.3 [0.8-2.3]	1.5 [1.0-2.6]	1.6 [1.0-2.5]	1.5 [0.9-2.6]	<0.001	DI vs RI, DW, RW
**CRP, mg/dL, median [IQR]**	16.6 [6.2-31.6]	13.0 [6.1-26.5]	14.9 [5.9-29.1]	15.9 [6.6-30.7]	21.9 [9.6-46.5]	0.160	
**GCS, median [IQR]**	14.0 [9.0-15.0]	14.0 [10.0-15.0]	13.0 [8.0-15.0]	12.0 [8.0-15.0]	14.0 [11.0-15.0]	<0.001	DI vs DW
**Glucose, mg/dL, median [IQR]**	141.0 [112.0-194.0]	138.0 [109.0-188.0]	143.0 [113.0-198.0]	143.0 [112.7-198.0]	138.0 [109.0-190.0]	<0.001	DI vs DW
**Heart rate, beats/min, median [IQR]**	106.0 [89.0-123.0]	107.0 [90.0-124.0]	103.0 [87.0-121.0]	106.0 [89.0-122.0]	109.0 [91.0-126.0]	<0.001	RI vs DI, DW, RW
**Hemoglobin, g/dL**	10.8 [9.1-12.6]	10.8 [9.0-12.6]	11.0 [9.3-12.6]	10.6 [9.0-12.5]	10.7 [9.0-12.6]	0.075	
**INR, median [IQR]**	1.3 [1.1-1.7]	1.3 [1.1-1.7]	1.2 [1.1-1.6]	1.3 [1.1-1.7]	1.3 [1.1-1.8]	<0.001	RI vs RW
**Lactate, mmol/L, median [IQR]**	1.7 [0.6-3.2]	1.5 [0.0-2.9]	1.6 [0.5-2.9]	1.75 [0.8-3.4]	2.0 [0.9-4.0]	<0.001[§]	DI vs RW
**lymphocyte percentage, %, median [IQR]**	6.8 [2.7-12.0]	6.7 [3.0-12.3]	7.0 [3.0-12.0]	7.0 [3.0-12.1]	6.0 [2.0-12.0]	<0.001	RW vs DI, RI, DW
**Pao2, mm Hg**	77.9 [61.0-108.0]	77.0 [60.0-107.0]	79.0 [61.4-108.0]	78.9 [61.0-112.0]	75.5 [60.4-105.0]	0.194	
**Platelet, x10(9)/L, median [IQR]**	191.0 [129.0-266.0]	187.0 [133.0-266.0]	200.0 [136.0-278.0]	184.0 [125.0-252.0]	176.0 [117.0-248.0]	<0.001[**]	RI vs DI, DW, RW
**RDW, %, median [IQR]**	15.3 [14.1-17.1]	15.5 [14.3-17.4]	15.2 [14.0-16.7]	15.6 [14.2-17.4]	15.6 [14.3-17.5]	<0.001	RI vs DI, DW, RW
**Respiratory rate, breaths/min, median [IQR]**	26.0 [21.0-33.0]	27.0 [22.0-33.0]	26.0 [20.0-32.0]	26.0 [21.0-32.0]	28.0 [22.0-35.0]	<0.001	RI vs DW
**SO2, %, median [IQR]**	93.0 [89.0-96.0]	92.0 [88.0-96.0]	93.0 [89.0-96.0]	93.0 [88.0-96.0]	92.0 [88.0-96.0]	<0.001	RI vs DI, RW
**Sodium, mmol/L, median [IQR]**	138.0 [135.0-141.0]	138.0 [135.0-141.0]	138.0 [135.0-141.0]	138.0 [135.0-142.0]	138.0 [134.0-141.0]	0.170	
**Systolic ABP, mm Hg, median [IQR]**	89.0 [76.0-104.0]	90.0 [77.0-106.0]	90.0 [76.0-104.0]	88.0 [76.0-102.0]	88.0 [74.0-104.0]	0.031	DI vs DW
**Temperature, degrees C, median [IQR]**	37.2 [36.8-38.1]	37.22 [36.8-38.1]	37.2 [36.8-38.1]	37.2 [36.7-38.0]	37.2 [36.7-38.0]	0.072	
**Troponin I, ng/mL, median [IQR]**	0.1 [0.04-0.38]	0.1 [0.03-0.4]	0.1 [0.03-0.33]	0.1 [0.04-0.58]	0.1 [0.04-0.4]	0.104	

**WBC, x10(9)/L, median [IQR]**	13.8 [9.0-19.5]	13.4 [8.8-19.3]	14.0 [9.5-19.4]	13.3 [8.8-19.4]	13.6 [8.3-19.9]	0.003	RI vs DW

Definition of abbreviations: IQR--interquartile range; SD--standard deviation; ALT-- alanine aminotransferase; AST-- alanine aminotransferase; BMI-- body mass index, BUN-- blood urea nitrogen; CRP-- C-reactive protein; GCS-- Glasgow coma scale score; RDW-- red blood cell distribution width; WBC--white blood cell count; DI-- Delayed Improving; RI--Rapidly Improving; DW-- Delayed Worsening; RW-- Rapidly Worsening.

† p-value calculated by analysis of variance (ANOVA)/Kruskal–Wallis test

** False discovery rate corrected p-value < 0.001

§ Age adjusted (analysis of covariance [ANCOVA]) p-value < 0.001

Table S11. Patient basic characteristics comparisons among subphenotypes in the CEDAR validation cohort

Characteristics	Total N=4,804	DI N=382	RI N=2,561	DW N=564	RW N=1,297	p-value†
**Age, median (IQR)**	77.0 [66.0-88.0]	77.0 [65.0-89.0]	78.0 [65.0-88.0]	79.0 [68.0-90.0]	76.0 [65.0-87.0]	0.010
**Sex, No. (%)**						0.257
Male	2320 (48.2%)	193 (50.5%)	1204 (47.0%)	274 (48.5%)	649 (50.0%)	
Female	2484 (51.7%)	189 (49.4%)	1357 (52.9%)	290 (51.4%)	648 (49.9%)	
**Race, No. (%)**						0.964
WHITE	1791 (37.2%)	140 (36.6%)	959 (37.4%)	210 (37.2%)	482 (37.1%)	
BLACK	446 (9.2%)	39 (10.2%)	228 (8.9%)	51 (9.0%)	128 (9.8%)	
OTHER	2567 (53.4%)	203 (53.1%)	1374 (53.6%)	303 (53.7%)	687 (52.9%)	
**Length stay, median (IQR)**	4.4 [2.6-7.9]	4.6 [2.8-7.8]	3.9 [2.4-6.7]	4.7 [2.8-7.9]	5.7 [2.8-10.8]	<0.001
**Mechanical ventilation at admission, No. (%)**	2263(47.1%)	151(39.5%)	1174(45.8%)	270(47.8%)	668(51.5%)	0.014
**Elixhauser index, median (IQR)**	12.5 [0.0-27.0]	14.0 [0.0-29.0]	11.0 [0.0-24.0]	15.0 [0.0-26.0]	16.0 [0.0-30.0]	<0.001
**Baseline SOFA, mean (SD)**	6.3 (3.1)	5.0 (2.8)	6.4 (3.0)	6.7 (3.2)	6.5 (3.2)	<0.001
**ICU unit at admission, No. (%)**						<0.001
CTICU	656 (13.6%)	31 (8.1%)	382 (14.9%)	126 (22.3%)	117 (9.0%)	
SICU	752 (15.6%)	60 (15.7%)	492 (19.2%)	56 (9.9%)	144 (11.1%)	
MICU	2641 (54.9%)	213 (55.7) %	1314 (51.3%)	295 (52.3%)	819 (63.1%)	
CICU	755 (15.7%)	78 (20.4%)	373 (14.5%)	87 (15.4%)	217 (16.7%)	
**Infection item, No. (%)**						
Central nervous system	38 (0.7%)	3 (0.7%)	23 (0.9%)	6 (1.0%)	6 (0.4%)	0.441
Intra-Abdominal	499 (10.3%)	39 (10.2%)	256 (10.0%)	54 (9.5%)	150 (11.5%)	0.428
Pneumonia	597 (12.4%)	52 (13.6%)	291 (11.3%)	63 (11.1%)	191 (14.7%)	0.016
Septicemia bacteremia	1376 (28.6%)	101 (26.4%)	684 (26.7%)	155 (27.4%)	436 (33.6%)	<0.001
Skin soft tissue	164 (3.4%)	17 (4.4%)	76 (2.9%)	24 (4.2%)	47 (3.6%)	0.243
Urinary tract	1127 (23.4%)	93 (24.3%)	657 (25.6%)	127 (22.5%)	250 (19.2%)	<0.001
**Septic shock, No. (%)**	564 (11.7%)	47 (12.3%)	247 (9.6%)	58 (10.2%)	212 (16.3%)	<0.001

Definition of abbreviations: IQR--interquartile range; SD--standard deviation; SOFA--Sequential Organ Failure Assessment; CTICU--Cardiothoracic ICU; SICU-- Surgical ICU; MICU-- Medical ICU; CICU-- Cardiac ICU; DI-- Delayed Improving; RI--Rapidly Improving; DW--Delayed Worsening; RW-- Rapidly Worsening.

†p-value calculated by Chi-square test/Fisher's exact test, or student's t-test/Mann-Whitney test where appropriate.

Table S12. Patient physiological characteristics comparisons among subphenotypes in the CEDAR validation cohort

Characteristics	Total N=4,804	DI N=382	RI N=2,561	DW N=564	RW N=1,297	p-value†	Post-hoc
Albumin, g/dL, median [IQR]	2.6 [2.1-3.2]	2.7 [2.2-3.2]	2.7 [2.3-3.2]	2.6 [2.1-3.1]	2.5 [1.9-3.0]	<0.001	DI vs RW
ALT, IU/L, median [IQR]	26.0 [17.0-53.0]	26.0 [17.0-46.75]	26.0 [17.0-49.75]	24.0 [16.0-56.0]	30.0 [18.0-64.0]	0.113	
AST, IU/L, median [IQR]	38.0 [25.0-75.0]	35.0 [24.0-62.0]	36.0 [24.0-67.0]	39.0 [26.0-93.0]	42.0 [26.0-93.0]	0.073	
Bands, median [IQR]	12.0 [5.0-21.0]	13.5 [7.0-22.0]	12.0 [5.0-21.0]	12.0 [7.0-23.75]	11.0 [6.0-21.0]	0.340	
Bicarbonate, mEq/L, median [IQR]	20.4 [16.4-23.8]	20.7 [16.7-23.9]	21.0 [17.6-24.4]	20.4 [16.7-23.4]	18.7 [14.0-22.8]	<0.001	RI vs RW
Bilirubin, mg/dL, median [IQR]	1.1 [0.7-1.7]	1.0 [0.7-1.8]	1.1 [0.7-1.7]	1.0 [0.7-1.6]	1.1 [0.7-1.8]	0.189	
BMI, kg/m2, median [IQR]	25.7 [22.0-30.4]	25.0 [21.7-28.7]	25.8 [22.2-30.6]	26.3 [22.0-30.6]	25.3 [21.7-30.1]	0.053	
BUN, mg/dL, median [IQR]	27.0 [16.0-48.0]	27.0 [16.0-48.2]	25.0 [15.0-45.0]	26.5 [16.0-46.7]	31.0 [18.0-53.0]	<0.001	RI vs DW
Chloride, mEq/L, median [IQR]	106.0 [101.0-110.0]	105.0 [100.0-109.0]	106.0 [101.0-109.0]	106.0 [102.0-110.0]	105.0 [101.0-110.0]	0.064	
Creatinine, mg/dL, median [IQR]	1.3 [0.9-2.3]	1.3 [0.9-2.6]	1.3 [0.8-2.1]	1.3 [0.9-2.1]	1.5 [0.9-2.4]	0.042	RW vs RI, DW
CRP, mg/dL, median [IQR]	15.8 [7.8-26.8]	19.1 [7.9-28.9]	13.9 [7.1-26.6]	18.7 [7.5-27.4]	13.54 [8.25-26.56]	0.926	
GCS, median [IQR]	12.0 [7.0-15.0]	15.0 [10.0-15.0]	10.0 [6.0-15.0]	10.0 [3.0-15.0]	14.0 [8.0-15.0]	<0.001	RW vs RI, DW; DI vs RI, DW
Glucose, mg/dL, median [IQR]	144.0 [113.0-189.0]	139.0 [109.2-187.0]	146.0 [114.0-189.0]	146.0 [114.0-198.0]	141.0 [111.0-187.0]	0.032	RW vs RI, DW
Heart rate, beats/min, median [IQR]	110.0 [92.0-130.0]	108.0 [93.0-129.0]	106.0 [90.0-126.0]	108.0 [91.0-128.0]	119.0 [99.0-139.0]	<0.001	RW vs DI, RI, DW
Hemoglobin, g/dL	9.9 [8.3-11.6]	9.8 [8.0-11.5]	10.1 [8.6-11.7]	9.7 [8.3-11.5]	9.6 [8.0-11.4]	<0.001	RI vs RW
INR, median [IQR]	1.3 [1.1-1.6]	1.2 [1.1-1.5]	1.2 [1.1-1.5]	1.3 [1.1-1.6]	1.4 [1.2-1.7]	<0.001	RW vs DI, RI
Lactate, mmol/L, median [IQR]	2.5 [1.3-4.8]	2.2 [1.1-4.4]	2.1 [1.2-4.0]	3.0 [1.6-5.4]	3.2 [1.5-6.4]	<0.001[§]	RW vs DI, RI; DW vs DI, RI
lymphocyte percentage, %, median [IQR]	8.4 [4.5-15.4]	8.3 [4.5-13.5]	8.7 [4.7-15.3]	8.5 [4.7-15.5]	8.0 [4.0-16.2]	0.563	
Pao2, mm Hg	92.0 [70.0-129.0]	89.0 [68.0-141.0]	96.0 [73.0-132.0]	92.0 [70.0-132.5]	84.0 [67.0-118.2]	0.283	
Platelet, x10(9)/L, median [IQR]	154.0 [93.0-229.0]	164.0 [104.0-225.0]	163.0 [101.7-242.0]	149.0 [87.5-223.0]	137.0 [66.5-204.0]	<0.001[**]	RW vs DI, RI, DW; RI vs DW
Respiratory rate, breaths/min, median [IQR]	27.0 [22.0-33.0]	28.0 [23.0-34.0]	26.0 [22.0-31.0]	26.0 [21.0-32.0]	30.0 [24.0-36.0]	<0.001	RW vs RI, DW
SO2, %, median [IQR]	94.0 [90.0-96.0]	94.0 [91.0-96.0]	94.0 [91.0-96.0]	94.0 [90.0-96.0]	92.0 [87.0-95.0]	<0.001	RW vs DI, RI, DW
Sodium, mmol/L, median [IQR]	138.0 [135.0-141.0]	138.0 [134.0-141.0]	138.0 [135.0-141.0]	139.0 [136.0-142.0]	138.0 [134.0-141.0]	0.172	
Systolic ABP, mm Hg, median [IQR]	88.0 [73.0-101.0]	89.0 [74.0-103.0]	91.0 [78.0-103.0]	87.0 [70.0-99.0]	79.0 [66.0-96.0]	<0.001	RW vs DI, RI, DW
Temperature, degrees C, median [IQR]	37.2 [36.8-38.1]	37.2 [36.8-38.4]	37.2 [36.8-38.0]	37.1 [36.8-37.9]	37.3 [36.8-38.4]	<0.001	DW vs RW
Troponin I, ng/mL, median [IQR]	0.1 [0.04-0.4]	0.1 [0.03-0.8]	0.08 [0.04-0.4]	0.1 [0.05-0.4]	0.1 [0.04-0.4]	0.050	

**WBC, x10(9)/L, median [IQR]**	11.5 [7.1-17.2]	11.0 [7.0-16.0]	12.0 [8.0-17.3]	12.1 [7.7-17.8]	10.1 [4.7-16.7]	<0.001	DW vs RW

Definition of abbreviations: IQR--interquartile range; SD--standard deviation; ALT-- alanine aminotransferase; AST-- alanine aminotransferase; BMI-- body mass index, BUN-- blood urea nitrogen; CRP-- C-reactive protein; GCS-- Glasgow coma scale score; WBC--white blood cell count; DI-- Delayed Improving; RI--Rapidly Improving; DW-- Delayed Worsening; RW-- Rapidly Worsening.

† p-value calculated by analysis of variance (ANOVA)/Kruskal–Wallis test

** False discovery rate corrected p-value < 0.001

§ Age adjusted (analysis of covariance [ANCOVA]) p-value < 0.001

Table S13. Patient physiological characteristics comparisons among subphenotypes in terms of sensitivity analysis (GBTM) in the development cohort

Characteristics	Total (N=4,678)	DI (N=817)	RI (N=2,402)	DW (N=735)	RW (N=724)	p-value†	Post-hoc
Albumin, g/dL, median [IQR]	2.9 [2.5-3.3]	2.9 [2.5-3.4]	2.9 [2.5-3.3]	3.0 [2.5-3.3]	2.9 [2.5-3.3]	0.581	
ALT, IU/L, median [IQR]	35.0 [19.0-86.0]	33.0 [20.0-79.5]	36.0 [19.0-89.0]	32.0 [18.0-78.7]	38.5 [20.0-82.2]	0.36	
AST, IU/L, median [IQR]	49.0 [27.0-120.0]	47.0 [29.0-128.0]	47.0 [26.0-111.0]	47.0 [24.0-127.0]	58.0 [30.5-142.0]	0.002	DI, RI, DW vs RW
Bands, median [IQR]	2.0 [0.0-8.0]	2.0 [0.0-8.2]	2.0 [0.0-9.0]	3.0 [0.0-8.0]	2.0 [0.0-8.0]	0.838	
Bicarbonate, mEq/L, median [IQR]	23.0 [20.0-26.0]	24.0 [21.0-26.0]	23.0 [20.0-25.0]	23.0 [19.0-25.0]	22.0 [19.0-26.0]	<0.001	DI vs RI, DW, RW
Bilirubin, mg/dL, median [IQR]	0.8 [0.4-2.1]	1.0 [0.5-2.3]	0.8 [0.4-2.0]	0.7 [0.4-1.7]	1.0 [0.5-2.6]	<0.001	DI vs RI, DW; RW vs RI, DW
BMI, kg/m2, median [IQR]	27.5 [23.7-32.2]	27.1 [23.5-32.0]	27.7 [23.8-32.6]	27.8 [24.2-32.2]	26.8 [23.4-31.4]	0.318	
BUN, mg/dL, median [IQR]	22.0 [15.0-38.0]	19.0 [13.0-30.0]	22.0 [15.0-37.0]	28.0 [16.0-49.0]	24.0 [15.0-41.0]	<0.001	DI vs RI, DW, RW; RI vs DW; DW vs RW
Chloride, mEq/L, median [IQR]	106.0 [102.0-110.0]	106.0 [101.0-109.0]	106.0 [102.0-110.0]	106.0 [102.0-110.0]	106.0 [101.0-110.0]	0.027	DI vs RI;
Creatinine, mg/dL, median [IQR]	1.1 [0.8-1.7]	0.9 [0.7-1.3]	1.1 [0.8-1.7]	1.3 [0.9-2.5]	1.1 [0.8-1.8]	<0.001	DI vs RI, DW, RW; RI vs DW; DW vs RW
CRP, mg/dL, median [IQR]	92.5 [40.5-174.4]	125.4 [57.3-186.6]	80.9 [29.2-171.3]	101.0 [49.7-165.0]	83.0 [42.6-164.8]	0.524	
GCS, median [IQR]	13.0 [7.0-15.0]	14.0 [7.0-15.0]	13.0 [7.0-15.0]	13.0 [7.0-15.0]	13.0 [6.0-15.0]	0.051	
Glucose, mg/dL, median [IQR]	131.0 [106.0-172.0]	129.0 [105.0-167.0]	132.0 [107.0-173.0]	131.0 [105.0-172.0]	135.0 [106.0-175.0]	0.321	
Heart rate, beats/min, median [IQR]	95.0 [81.0-110.0]	96.0 [82.0-110.0]	94.0 [80.6-109.0]	95.0 [80.63-110.0]	96.0 [83.8-112.0]	0.043	RI, DW vs RW
Hemoglobin, g/dL	10.6 [9.2-12.0]	10.6 [9.3-11.9]	10.6 [9.3-12.0]	10.4 [9.1-11.9]	10.5 [9.1-12.01]	0.559	
INR, median [IQR]	1.3 [1.2-1.6]	1.3 [1.1-1.6]	1.3 [1.1-1.5]	1.3 [1.2-1.7]	1.4 [1.2-1.7]	<0.001**	DI vs DW, RW; RI vs DW, RW
Lactate, mmol/L, median [IQR]	1.8 [1.2-2.8]	1.7 [1.2-2.8]	1.7 [1.1-2.7]	1.8 [1.2-3.0]	2.1 [1.3-3.2]	<0.001§	DI, RI, DW vs RW
lymphocyte count, median [IQR]	940.0 [675.0-1570.0]	1161.0 [1020.0-2016.0]	840.0 [584.5-1144.0]	1095.0 [431.2-1823.2]	782.0 [537.5-1502.5]	0.422	
lymphocyte percentage, %, median [IQR]	7.1 [3.5-12.5]	7.6 [4.0-12.4]	7.1 [3.5-12.6]	7.0 [3.5-12.2]	6.7 [3.4-13.1]	0.608	
Pao2, mm Hg	108.0 [81.5-168.5]	101.0 [81.5-128.5]	106.0 [71.0-146.5]	138.0 [91.2-181.5]	135.0 [89.5-202.7]	0.189	
Platelet, x10(9)/L, median [IQR]	190.0 [130.0-260.0]	184.0 [126.0-259.0]	199.0 [137.0-265.0]	189.5 [131.0-257.2]	168.0 [115.0-238.0]	<0.001	DI vs RI, RW; RI, DW vs RW
RDW, %, median [IQR]	14.8 [13.8-16.3]	14.8 [13.8-16.2]	14.7 [13.7-16.1]	14.9 [13.8-16.7]	14.9 [13.8-16.8]	<0.001	RI vs DW, RW
Respiratory rate, breaths/min, median [IQR]	22.0 [19.0-27.0]	22.6 [19.0-27.0]	22.0 [19.0-26.0]	22.0 [19.0-26.1]	23.0 [19.0-27.5]	0.085	
SO2, %, median [IQR]	94.0 [80.0-97.0]	94.6 [83.0-97.0]	93.0 [78.0-97.0]	95.0 [82.0-97.2]	94.0 [81.0-97.0]	0.064	

**Sodium, mmol/L, median [IQR]**	139.0 [136.0-142.0]	139.0 [136.0-141.0]	139.0 [136.0-141.0]	139.0 [136.0-142.0]	139.0 [136.0-142.0]	0.085	
**Systolic ABP, mm Hg, median [IQR]**	101.0 [91.0-115.0]	102.5 [92.0-118.0]	100.0 [91.0-113.0]	102.0 [91.5-115.0]	102.0 [91.3-117.0]	<0.001	DI vs RI; RI vs DW, RW;
**Temperature, degrees C, median [IQR]**	36.8 [36.3-37.4]	36.8 [36.3-37.3]	36.8 [36.3-37.5]	36.8 [36.2-37.4]	36.7 [36.2-37.3]	0.011	RI vs DW, RW
**Troponin T, ng/mL, median [IQR]**	0.1 [0.03-0.3]	0.1 [0.03-0.2]	0.1 [0.03-0.3]	0.1 [0.04-0.3]	0.1 [0.03-0.3]	0.129	
**WBC, x10(9)/L, median [IQR]**	11.1 [7.5-15.8]	10.4 [7.0-14.5]	11.4 [7.8-16.3]	11.0 [7.4-15.0]	10.9 [7.4-15.8]	<0.001	DI vs RI, RI vs DW

Definition of abbreviations: IQR--interquartile range; SD--standard deviation; ALT-- alanine aminotransferase; AST-- alanine aminotransferase; BMI-- body mass index, BUN-- blood urea nitrogen; CRP-- C-reactive protein; GCS-- Glasgow coma scale score; INR-- international normalized ratio; RDW-- red blood cell distribution width; ABP-- Ambulatory Blood Pressure; WBC--white blood cell count; DI-- Delayed Improving; RI-- Rapidly Improving; DW-- Delayed Worsening; RW-- Rapidly Worsening.

† p-value calculated by analysis of variance (ANOVA)/Kruskal–Wallis test

** False discovery rate corrected p-value < 0.001

§ Age adjusted (analysis of covariance [ANCOVA]) p-value < 0.001

Table S14. Missing data (no., %) in cohorts for subphenotyping and predicting variables at the first six hours

Variable	Development Cohort (MIMIC-III)	Validation Cohort (NMEDW)	Validation Cohort (eICU)	Validation Cohort (CEDAR)
No. of patients	4,678	3,665	12,282	4,804
Age	349 (7.4%)	0 (0.0%)	0 (0.0%)	0 (0.0%)
Albumin	2239 (47.8%)	917 (25.0%)	3055 (24.8%)	1103 (22.9%)
ALT	1746 (37.3%)	984 (26.8%)	3040 (24.7%)	1164 (24.2%)
AST	1747 (37.3%)	976 (26.6%)	2972 (24.1%)	1162 (24.1%)
Bands	3425 (73.2%)	3083 (84.1%)	9550 (81.0%)	3779 (78.6%)
Bicarbonate	24 (0.5%)	295 (8.0%)	533 (4.3%)	261 (5.4%)
Bilirubin	1729 (36.9%)	593 (16.1%)	3232 (26.3%)	1120 (23.3%)
PaO2	1532 (32.7%)	475 (12.9%)	475 (33.1%)	1398 (29.1%)
BMI	2088 (44.6%)	698 (19.0%)	115 (0.9%)	1786 (37.1%)
BUN	26 (0.5%)	12 (0.3%)	603 (4.9%)	247 (5.1%)
Chloride	23 (0.4%)	16 (0.4%)	601 (4.8%)	243 (5.0%)
Creatinine	24 (0.5%)	10 (0.2%)	605 (4.9%)	243 (5.0%)
CRP	4522 (96.6%)	3398 (92.7%)	11737 (95.5%)	4566 (95.0%)
GCS	2 (0.04%)	121 (3.3%)	2949 (24.0%)	238 (4.9%)
Gender	0 (0.0%)	0 (0.0%)	0 (0.0%)	0 (0.0%)
Glucose	22 (0.4%)	14 (0.3%)	165 (1.3%)	261 (5.4%)
Heart rate	0 (0.0%)	2 (0.05%)	626 (5.0%)	87 (1.8%)
Hemoglobin	31 (0.6%)	17 (0.4%)	357 (2.9%)	296 (2.1%)
INR	511 (10.9%)	977 (26.6%)	3555 (28.9%)	716 (14.9%)
Lactate	1393 (29.7%)	723 (19.7%)	3240 (26.3%)	1322 (27.5%)
Lymphocyte percent	2012 (43.0%)	2206 (60.1%)	3025 (24.6%)	1971 (41.0%)
Platelet	32 (0.6%)	10 (0.2%)	439 (3.5%)	35 (0.7%)
Race	0 (0.0%)	0 (0.0%)	0 (0.0%)	0 (0.0%)
RDW	34 (0.7%)	25 (0.6%)	808(6.5%)	-----
Respiratory rate	0 (0.0%)	120 (3.2%)	866 (7.0%)	88 (1.8%)
SO2	8 (0.17%)	1 (0.03%)	269 (2.1%)	87 (1.8%)
Sodium	23 (0.4%)	14 (0.3%)	269 (2.1%)	18 (0.3%)
Systolic ABP	1 (0.02%)	7 (0.19%)	748 (6.0%)	90 (1.8%)
Temperature	12 (0.2%)	2 (0.05%)	39 (3.0%)	90 (1.8%)
Troponin	3147 (67.2%)	1922 (52.4%)	7758 (63.1%)	2798 (58.2%)
WBC	32 (0.6%)	22 (0.6%)	407 (3.3%)	60 (1.2%)

Definition of abbreviations: ALT-- alanine aminotransferase; AST-- alanine aminotransferase; BMI-- body mass index, BUN-- blood urea nitrogen; CRP-- C-reactive protein; GCS-- Glasgow coma scale score; INR-- international normalized ratio; RDW-- red blood cell distribution width; ABP: Ambulatory Blood Pressure; WBC--white blood cell count.

Table S15. International classification of diseases, ninth revision, clinical modification codes used to identify infectious syndromes.

Infectious Syndrome	ICD-9-CM Codes
Pneumonia	480.0-480.9, 481, 482.0-482.9, 483.0-483.8, 484.1-484.8, 485, 486
Urinary Tract Infections	590.00, 590.01, 590.10, 590.11, 590.2, 590.3, 590.80, 590.81, 590.9, 595.0, 595.2, 595.3, 595.4, 595.89, 595.9, 597.0, 597.80, 597.89, 598.00, 598.01, 599.0
Intra-abdominal Infections	008.45, 009.0-009.3, 540.0-540.9, 541, 542, 543.9, 562.01, 562.03, 562.11, 562.13, 567.0-567.9, 569.5, 569.61, 569.71, 569.83, 572.0-572.8, 574.00-574.91, 575.0-575.9, 576.0-576.9, 614.0-614.9
Skin/Soft Tissue Infections	680-686, 035, 376.01, 728.86
Central Nervous System	006.5, 013.00-013.06, 013.10-013.16, 013.20-013.26, 013.30-013.36, 013.40-013.46, 013.50-013.56, 013.60-013.66, 013.80-013.86, 013.90-013.96, 036.0, 036.1, 049.8, 052.0, 052.2, 053.0, 053.14, 054.3, 054.72, 055.0, 056.01, 064, 072.1, 072.2, 091.81, 094.2, 098.82, 100.81, 112.83, 114.2, 130.0, 320.0-320.3, 320.7, 320.81, 323.01, 323.02, 323.1, 323.2, 323.41, 323.42, 324.0, 324.1, 324.9
Septicemia/Bacteremia	038.0-038.9, 790.7

Table S16. The associations between comorbidities and subphenotypes in the MIMIC-III development cohort

Comorbidity Name	Total (N=4,678)	DI (N=1,174)	RI (N=1,932)	DW (N=960)	RW (N=612)	p-value
Congestive heart failure, No. (%)	1327 (28.3%)	372 (31.6%)	502 (25.9%)	302 (31.4%)	151 (24.6%)	<0.001
Valvular disease, No. (%)	544 (11.6%)	173 (14.7%)	199 (10.3%)	107 (11.1%)	65 (10.6%)	0.002
Pulmonary circulation disorder, No. (%)	514 (10.9%)	161 (13.7%)	177 (9.1%)	109 (11.3%)	67 (10.9%)	0.001
Peripheral vascular disorder, No. (%)	469 (10.0%)	123 (10.4%)	177 (9.1%)	117 (12.1%)	52 (8.5%)	0.038
Hypertension, No. (%)	2894 (61.8%)	734 (62.5%)	1165 (60.3%)	619 (64.4%)	376 (61.4%)	0.168
Paralysis, No. (%)	173 (3.7%)	43 (3.6%)	68 (3.5%)	36 (3.7%)	26 (4.2%)	0.872
Other neurological, No. (%)	903 (19.3%)	232 (19.7%)	372 (19.2%)	191 (19.9%)	108 (17.6%)	0.693
Chronic pulmonary disease, No. (%)	1263 (27.0%)	345 (29.3%)	496 (25.6%)	261 (27.1%)	161 (26.3%)	0.152
Diabetes w/o chronic complications, No. (%)	1147 (24.5%)	300 (25.5%)	473 (24.4%)	248 (25.8%)	126 (20.5%)	0.083
Diabetes w/ chronic complications, No. (%)	380 (8.1%)	80 (6.8%)	149 (7.7%)	104 (10.8%)	47 (7.6%)	0.005
Hypothyroidism, No. (%)	659 (14.0%)	171 (14.5%)	273 (14.1%)	137 (14.2%)	78 (12.7%)	0.762
Renal failure, No. (%)	1075 (22.9%)	212 (18.0%)	408 (21.1%)	327 (34.0%)	128 (20.9%)	<0.001
Liver disease, No. (%)	852 (18.2%)	233 (19.8%)	289 (14.9%)	163 (16.9%)	167 (27.2%)	<0.001
Chronic Peptic ulcer disease, No. (%)	93 (1.9%)	28 (2.3%)	37 (1.9%)	16 (1.6%)	12 (1.9%)	0.679
HIV_AIDS, No. (%)	41 (0.8%)	8 (0.6%)	20 (1.0%)	10 (1.0%)	3 (0.4%)	0.489
Lymphoma, No. (%)	118 (2.5%)	31 (2.6%)	44 (2.2%)	28 (2.9%)	15 (2.4%)	0.763
Metastatic cancer, No. (%)	359 (7.6%)	86 (7.3%)	151 (7.8%)	75 (7.8%)	47 (7.6%)	0.963
Solid tumor without metastasis, No. (%)	536 (11.4%)	132 (11.2%)	226 (11.7%)	105 (10.9%)	73 (11.9%)	0.907
Rheumatoid arthritis/collagen vascular diseases, No. (%)	244 (5.2%)	55 (4.6%)	99 (5.1%)	56 (5.8%)	34 (5.5%)	0.663
Coagulation deficiency, No. (%)	1007 (21.5%)	249 (21.2%)	366 (18.9%)	205 (21.3%)	187 (30.5%)	<0.001
Obesity, No. (%)	383 (8.1%)	107 (9.1%)	151 (7.8%)	78 (8.1%)	47 (7.6%)	0.591
Weight loss, No. (%)	408 (8.7%)	111 (9.4%)	165 (8.5%)	78 (8.1%)	54 (8.8%)	0.727
Fluid and electrolyte disorders, No. (%)	2537 (54.2%)	615 (52.3%)	1031 (53.3%)	547 (56.9%)	344 (56.2%)	0.108
Blood loss anemia, No. (%)	103 (2.2%)	40 (3.4%)	28 (1.4%)	23 (2.4%)	12 (1.9%)	0.004
Deficiency anemias, No. (%)	214 (4.5%)	63 (5.3%)	83 (4.3%)	42 (4.3%)	26 (4.2%)	0.519
Alcohol abuse, No. (%)	562 (12.0%)	157 (13.3%)	217 (11.2%)	96 (10.0%)	92 (15.0%)	0.007
Drug abuse, No. (%)	285 (6.0%)	62 (5.2%)	130 (6.7%)	56 (5.8%)	37 (6.0%)	0.418
Psychoses, No. (%)	112 (2.3%)	26 (2.2%)	51 (2.6%)	28 (2.9%)	7 (1.1%)	0.118
Depression, No. (%)	788 (16.8%)	189 (16.1%)	336 (17.3%)	171 (17.8%)	92 (15.0%)	0.399

Table S17. The associations between comorbidities and subphenotypes in the NMEDW validation cohort

Comorbidity Name	Total N=3,665	DI N=668	RI N=2,019	DW N=302	RW N=676	p-value
Congestive heart failure, No. (%)	664 (18.1%)	127 (19.0%)	338 (16.7%)	67 (22.1%)	132 (19.5%)	0.065
Valvular disease, No. (%)	257 (7.0%)	49 (7.3%)	129 (6.3%)	23 (7.6%)	56 (8.2%)	0.368
Pulmonary circulation disorder, No. (%)	266 (7.2%)	46 (6.8%)	138 (6.8%)	16 (5.3%)	66 (9.7%)	0.034
Peripheral vascular disorder, No. (%)	251 (6.8%)	45 (6.7%)	125 (6.1%)	20 (6.6%)	61 (9.0%)	0.093
Hypertension, No. (%)	1005 (27.4%)	202 (30.2%)	543 (26.8%)	95 (31.4%)	165 (24.4%)	0.037
Paralysis, No. (%)	91 (2.4%)	12 (1.8%)	45 (2.2%)	11 (3.6%)	23 (3.4%)	0.118
Other neurological, No. (%)	841 (22.9%)	154 (23.0%)	418 (20.7%)	84 (27.8%)	185 (27.3%)	0.001
Chronic pulmonary disease, No. (%)	549 (14.9%)	98 (14.6%)	299 (14.8%)	52 (17.2%)	100 (14.7%)	0.728
Diabetes w/o chronic complications, No. (%)	423 (11.5%)	78 (11.6%)	224 (11.0%)	41 (13.5%)	80 (11.8%)	0.640
Diabetes w/ chronic complications, No. (%)	255 (6.9%)	49 (7.3%)	132 (6.5%)	28 (9.2%)	46 (6.8%)	0.359
Hypothyroidism, No. (%)	198 (5.4%)	43 (6.4%)	101 (5.0%)	14 (4.6%)	40 (5.9%)	0.435
Renal failure, No. (%)	519 (14.1%)	105 (15.7%)	237 (11.7%)	58 (19.2%)	119 (17.6%)	<0.001
Liver disease, No. (%)	371 (10.1%)	73 (10.9%)	165 (8.1%)	31 (10.2%)	102 (15.0%)	<0.001
Chronic Peptic ulcer disease, No. (%)	49 (1.3%)	11 (1.6%)	21 (1.0%)	5 (1.6%)	12 (1.7%)	0.384
HIV_AIDS, No. (%)	27 (0.7%)	3 (0.4%)	17 (0.8%)	3 (0.9%)	4 (0.5%)	0.676
Lymphoma, No. (%)	94 (2.5%)	19 (2.8%)	41 (2.0%)	7 (2.3%)	27 (3.9%)	0.044
Metastatic cancer, No. (%)	173 (4.7%)	40 (5.9%)	80 (3.9%)	15 (4.9%)	38 (5.6%)	0.101
Solid tumor without metastasis, No. (%)	305 (8.3%)	67 (10.0%)	153 (7.5%)	18 (5.9%)	67 (9.9%)	0.037
Rheumatoid arthritis/collagen vascular diseases, No. (%)	90 (2.4%)	23 (3.4%)	39 (1.9%)	9 (2.9%)	19 (2.8%)	0.125
Coagulation deficiency, No. (%)	470 (12.8%)	97 (14.5%)	206 (10.2%)	47 (15.5%)	120 (17.7%)	<0.001
Obesity, No. (%)	167 (4.5%)	29 (4.3%)	94 (4.6%)	14 (4.6%)	30 (4.4%)	0.986
Weight loss, No. (%)	434 (11.8%)	92 (13.7%)	193 (9.5%)	41 (13.5%)	108 (15.9%)	<0.001
Fluid and electrolyte disorders, No. (%)	1397 (38.1%)	273 (40.8%)	676 (33.4%)	137 (45.3%)	311 (46.0%)	<0.001
Blood loss anemia, No. (%)	108 (2.9%)	22 (3.2%)	45 (2.2%)	12 (3.9%)	29 (4.2%)	0.026
Deficiency anemias, No. (%)	126 (3.4%)	23 (3.4%)	60 (2.9%)	15 (4.9%)	28 (4.1%)	0.216
Alcohol abuse, No. (%)	221 (6.0%)	45 (6.7%)	111 (5.5%)	20 (6.6%)	45 (6.6%)	0.521
Drug abuse, No. (%)	96 (2.6%)	17 (2.5%)	55 (2.7%)	7 (2.3%)	17 (2.5%)	0.971
Psychoses, No. (%)	66 (1.8%)	10 (1.5%)	43 (2.1%)	7 (2.3%)	6 (0.8%)	0.156
Depression, No. (%)	210 (5.7%)	40 (5.9%)	114 (5.6%)	23 (7.6%)	33 (4.8%)	0.392

Table S18. The associations between comorbidities and subphenotypes in the eICU validation cohort

Comorbidity Name	Total N=12,282	DI N=2,005	RI N=5,938	DW N=1,392	RW N=2,947	p-value
Congestive heart failure, No. (%)	1518 (12.3%)	288 (14.3%)	628 (10.5%)	205 (14.7%)	397 (13.4%)	<0.001
Valvular disease, No. (%)	119 (0.9%)	33 (1.6%)	35 (0.5%)	20 (1.4%)	31 (1.1%)	<0.001
Pulmonary circulation disorder, No. (%)	181 (1.4%)	35 (1.7%)	78 (1.3%)	19 (1.3%)	49 (1.6%)	0.406
Peripheral vascular disorder, No. (%)	86 (0.7%)	16 (0.8%)	26 (0.4%)	11 (0.7%)	33 (1.1%)	0.003
Hypertension, No. (%)	1456 (11.8%)	270 (13.4%)	633 (10.6%)	193 (13.8%)	360 (12.2%)	<0.001
Paralysis, No. (%)	74 (0.6%)	17 (0.8%)	40 (0.6%)	6 (0.4%)	11 (0.3%)	0.122
Other neurological, No. (%)	1427 (11.6%)	261 (13.0%)	665 (11.2%)	184 (13.2%)	317 (10.7%)	0.015
Chronic pulmonary disease, No. (%)	1601 (13.0%)	311 (15.5%)	791 (13.3%)	189 (13.5%)	310 (10.5%)	<0.001
Diabetes w/o chronic complications, No. (%)	444 (3.6%)	65 (3.2%)	242 (4.0%)	46 (3.3%)	91 (3.0%)	0.067
Hypothyroidism, No. (%)	508 (4.1%)	89 (4.4%)	234 (3.9%)	66 (4.7%)	119 (4.0%)	0.495
Renal failure, No. (%)	1755 (14.2%)	297 (14.8%)	755 (12.7%)	247 (17.7%)	456 (15.4%)	<0.001
Liver disease, No. (%)	670 (5.4%)	103 (5.1%)	239 (4.0%)	85 (6.1%)	243 (8.2%)	<0.001
Chronic Peptic ulcer disease, No. (%)	76 (0.6%)	14 (0.7%)	30 (0.5%)	17 (1.2%)	15 (0.5%)	0.017
Lymphoma, No. (%)	127 (1.0%)	28 (1.4%)	41 (0.6%)	19 (1.3%)	39 (1.3%)	0.004
Metastatic cancer, No. (%)	174 (1.4%)	31 (1.5%)	68 (1.1%)	12 (0.8%)	63 (2.1%)	0.001
Solid tumor without metastasis, No. (%)	500 (4.0%)	91 (4.5%)	199 (3.3%)	52 (3.7%)	158 (5.3%)	<0.001
Rheumatoid arthritis/collagen vascular diseases, No. (%)	97 (0.7%)	15 (0.7%)	40 (0.6%)	12 (0.8%)	30 (1.0%)	0.374
Coagulation deficiency, No. (%)	1108 (9.0%)	200 (9.9%)	396 (6.6%)	159 (11.4%)	353 (11.9%)	<0.001
Obesity, No. (%)	211 (1.7%)	60 (2.9%)	85 (1.4%)	14 (1.0%)	52 (1.7%)	<0.001
Weight loss, No. (%)	777 (6.3%)	164 (8.1%)	313 (5.2%)	118 (8.4%)	182 (6.1%)	<0.001
Fluid and electrolyte disorders, No. (%)	2783 (22.6%)	473 (23.5%)	1273 (21.4%)	350 (25.1%)	687 (23.3%)	0.009
Blood loss anemia, No. (%)	21 (0.1%)	4 (0.2%)	11 (0.1%)	0 (0.0%)	6 (0.2%)	0.434
Deficiency anemias, No. (%)	6 (0.1%)	4 (0.2%)	1 (0.02%)	1 (0.0%)	0 (0.0%)	0.007
Alcohol abuse, No. (%)	318 (2.5%)	51 (2.5%)	133 (2.2%)	42 (3.0%)	92 (3.1%)	0.065
Drug abuse, No. (%)	23 (0.1%)	7 (0.3%)	12 (0.2%)	1 (0.1%)	3 (0.1%)	0.170
Psychoses, No. (%)	80 (0.6%)	14 (0.7%)	40 (0.6%)	10 (0.7%)	16 (0.5%)	0.862
Depression, No. (%)	50 (0.4%)	8 (0.4%)	16 (0.2%)	4 (0.2%)	22 (0.7%)	0.009

Table S19. The associations between comorbidities and subphenotypes in the CEDAR validation cohort

Comorbidity Name	Total N=4,804	DI N=382	RI N=2,561	DW N=564	RW N=1,297	p-value
Congestive heart failure, No. (%)	1100 (22.9%)	94 (24.6%)	575 (22.4%)	157 (27.8%)	274 (21.1%)	0.012
Valvular disease, No. (%)	732 (15.2%)	50 (13.0%)	408 (15.9%)	114 (20.2%)	160 (12.3%)	<0.001
Pulmonary circulation disorder, No. (%)	690 (14.3%)	61 (15.9%)	361 (14.1%)	103 (18.2%)	165 (12.7%)	0.013
Peripheral vascular disorder, No. (%)	398 (8.2%)	31 (8.1%)	203 (7.9%)	56 (9.9%)	108 (8.3%)	0.483
Hypertension, No. (%)	1776 (36.9%)	130 (34.0%)	986 (38.5%)	221 (39.1%)	439 (33.8%)	0.014
Paralysis, No. (%)	148 (3.0%)	7 (1.8%)	85 (3.3%)	23 (4.0%)	33 (2.5%)	0.132
Other neurological, No. (%)	786 (16.3%)	63 (16.4%)	386 (15.0%)	86 (15.2%)	251 (19.3%)	0.007
Chronic pulmonary disease, No. (%)	835 (17.3%)	66 (17.2%)	471 (18.3%)	118 (20.9%)	180 (13.8%)	<0.001
Diabetes w/o chronic complications, No. (%)	678 (14.1%)	47 (12.3%)	404 (15.7%)	85 (15.0%)	142 (10.9%)	<0.001
Diabetes w/ chronic complications, No. (%)	328 (6.8%)	18 (4.7%)	191 (7.4%)	40 (7.0%)	79 (6.0%)	0.141
Hypothyroidism, No. (%)	464 (9.6%)	36 (9.4%)	251 (9.8%)	58 (10.2%)	119 (9.1%)	0.877
Renal failure, No. (%)	932 (19.4%)	77 (20.1%)	464 (18.1%)	116 (20.5%)	275 (21.2%)	0.111
Liver disease, No. (%)	402 (8.3%)	36 (9.4%)	165 (6.4%)	48 (8.5%)	153 (11.8%)	<0.001
Chronic Peptic ulcer disease, No. (%)	76 (1.5%)	11 (2.8%)	41 (1.6%)	9 (1.6%)	15 (1.1%)	0.130
Lymphoma, No. (%)	217 (4.5%)	24 (6.2%)	95 (3.7%)	29 (5.1%)	69 (5.3%)	0.028
Metastatic cancer, No. (%)	289 (6.0%)	26 (6.8%)	127 (4.9%)	27 (4.7%)	109 (8.4%)	<0.001
Solid tumor without metastasis, No. (%)	381 (7.9%)	38 (9.9%)	188 (7.3%)	38 (6.7%)	117 (9.0%)	0.087
Rheumatoid arthritis/collagen vascular diseases, No. (%)	150 (3.1%)	12 (3.1%)	79 (3.0%)	10 (1.7%)	49 (3.7%)	0.154
Coagulation deficiency, No. (%)	931 (19.3%)	68 (17.8%)	396 (15.4%)	121 (21.4%)	346 (26.6%)	<0.001
Obesity, No. (%)	286 (5.95%)	17 (4.4%)	168 (6.5%)	38 (6.7%)	63 (4.8%)	0.085
Weight loss, No. (%)	529 (11.0%)	44 (11.5%)	290 (11.3%)	56 (9.9%)	139 (10.7%)	0.766
Fluid and electrolyte disorders, No. (%)	2099 (43.6%)	162 (42.4%)	1063 (41.5%)	259 (45.9%)	615 (47.4%)	0.003
Blood loss anemia, No. (%)	45 (0.9%)	4 (1.0%)	22 (0.8%)	7 (1.2%)	12 (0.9%)	0.854
Deficiency anemias, No. (%)	132 (2.7%)	11 (2.8%)	68 (2.6%)	26 (4.6%)	27 (2.0%)	0.023
Alcohol abuse, No. (%)	172 (3.5%)	10 (2.6%)	92 (3.5%)	21 (3.7%)	49 (3.7%)	0.751
Drug abuse, No. (%)	110 (2.2%)	6 (1.5%)	67 (2.6%)	11 (1.9%)	26 (2.0%)	0.413
Psychoses, No. (%)	55 (1.1%)	4 (1.0%)	38 (1.4%)	6 (1.0%)	7 (0.5%)	0.076
Depression, No. (%)	214 (4.4%)	15 (3.9%)	119 (4.6%)	23 (4.0%)	57 (4.3%)	0.880

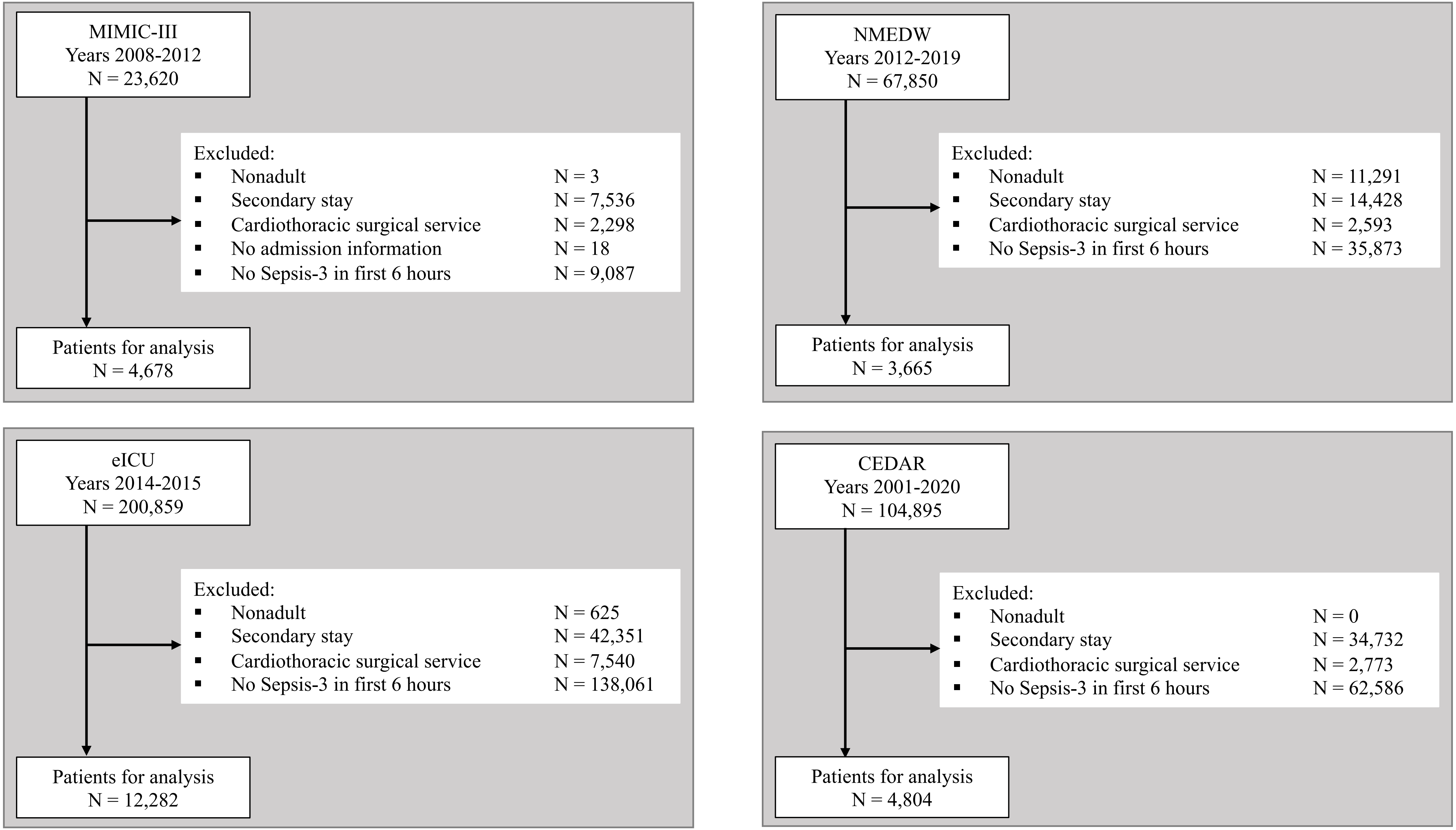
MIMIC-III
Years 2008-2012
N = 23,620
Excluded:
Nonadult N = 3
Secondary stay N = 7,536
Cardiothoracic surgical service N = 2,298
No admission information N = 18
No Sepsis-3 in first 6 hours N = 9,087
Patients for analysis
N = 4,678
NMEDW
Years 2012-2019
N = 67,850
Excluded:
Nonadult N = 11,291
Secondary stay N = 14,428
Cardiothoracic surgical service N = 2,593
No Sepsis-3 in first 6 hours N = 35,873
Patients for analysis
N = 3,665
eICU
Years 2014-2015
N = 200,859
Excluded:
Nonadult N = 625
Secondary stay N = 42,351
Cardiothoracic surgical service N = 7,540
No Sepsis-3 in first 6 hours N = 138,061
Patients for analysis
N = 12,282
CEDAR
Years 2001-2020
N = 104,895
Excluded:
Nonadult N = 0
Secondary stay N = 34,732
Cardiothoracic surgical service N = 2,773
No Sepsis-3 in first 6 hours N = 62,586
Patients for analysis
N = 4,804

Figure S1. Patient exclusion criteria for building development and validation cohorts

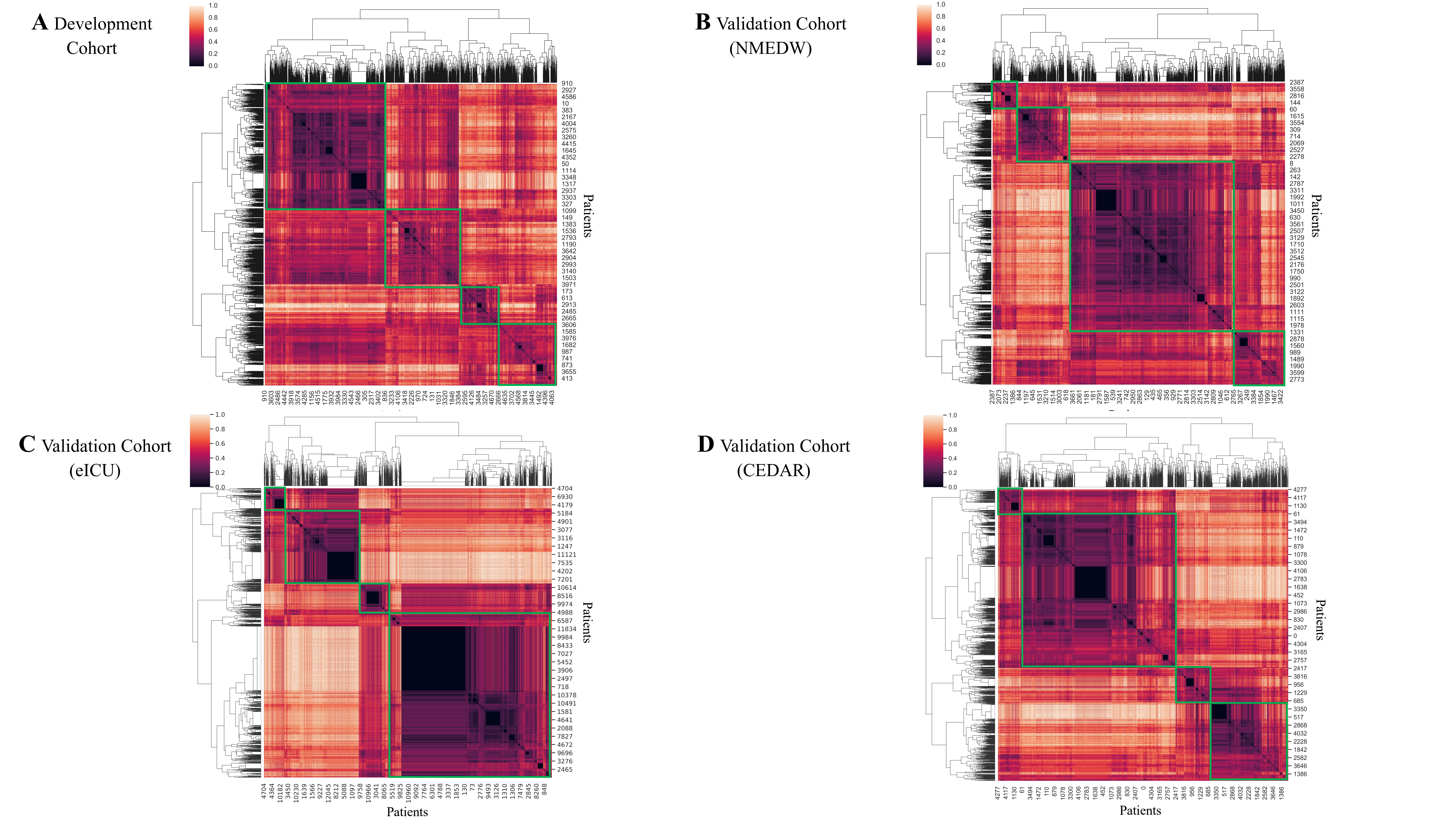
A Development Cohort
B Validation Cohort (NMEDW)
C Validation Cohort (eICU)
D Validation Cohort (CEDAR)
1.0
0.8
0.6
0.4
0.2
0.0
Patients

Figure S2. Clustergrams of hierarchical clustering. Horizontal and vertical axes represent patients. Color intensity denotes normalized pairwise patient similarity derived using Dynamic Time Warping (DTW). All clustergrams suggest optimal cluster number 4.

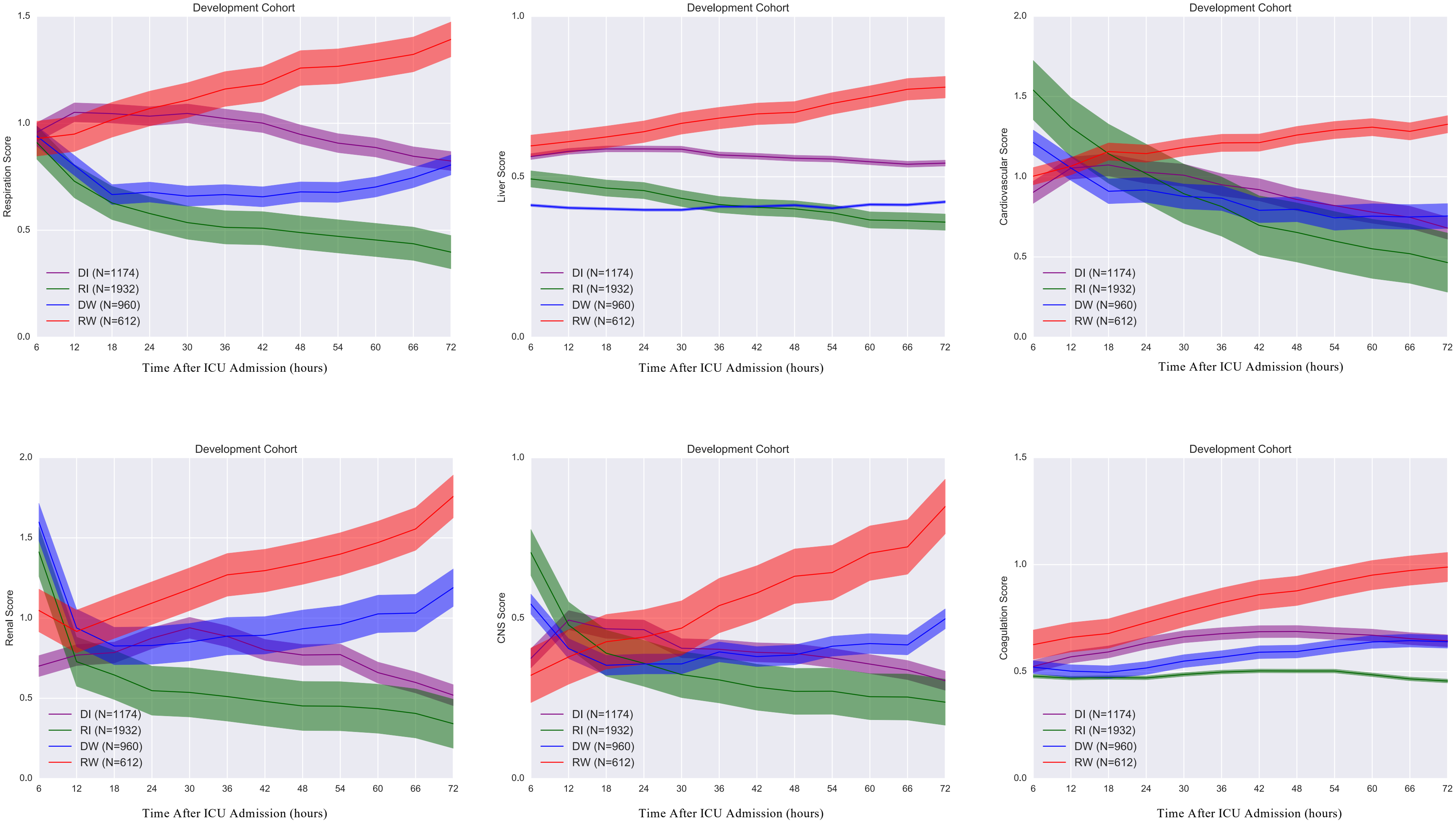

Development Cohort
Respiration Score
Liver Score
Cardiovascular Score
Renal Score
CNS Score
Coagulation Score
Time After ICU Admission (hours)
6
12
18
24
30
36
42
48
54
60
66
72
0.0
0.5
1.0
1.5
2.0
DI (N=1174)
RI (N=1932)
DW (N=960)
RW (N=612)

Figure S3. The trajectories of the subphenotypes in terms of subscores in the development cohort. DI: Delayed Improving; RI: Rapidly Improving; DW: Delayed Worsening; RW: Rapidly Worsening.

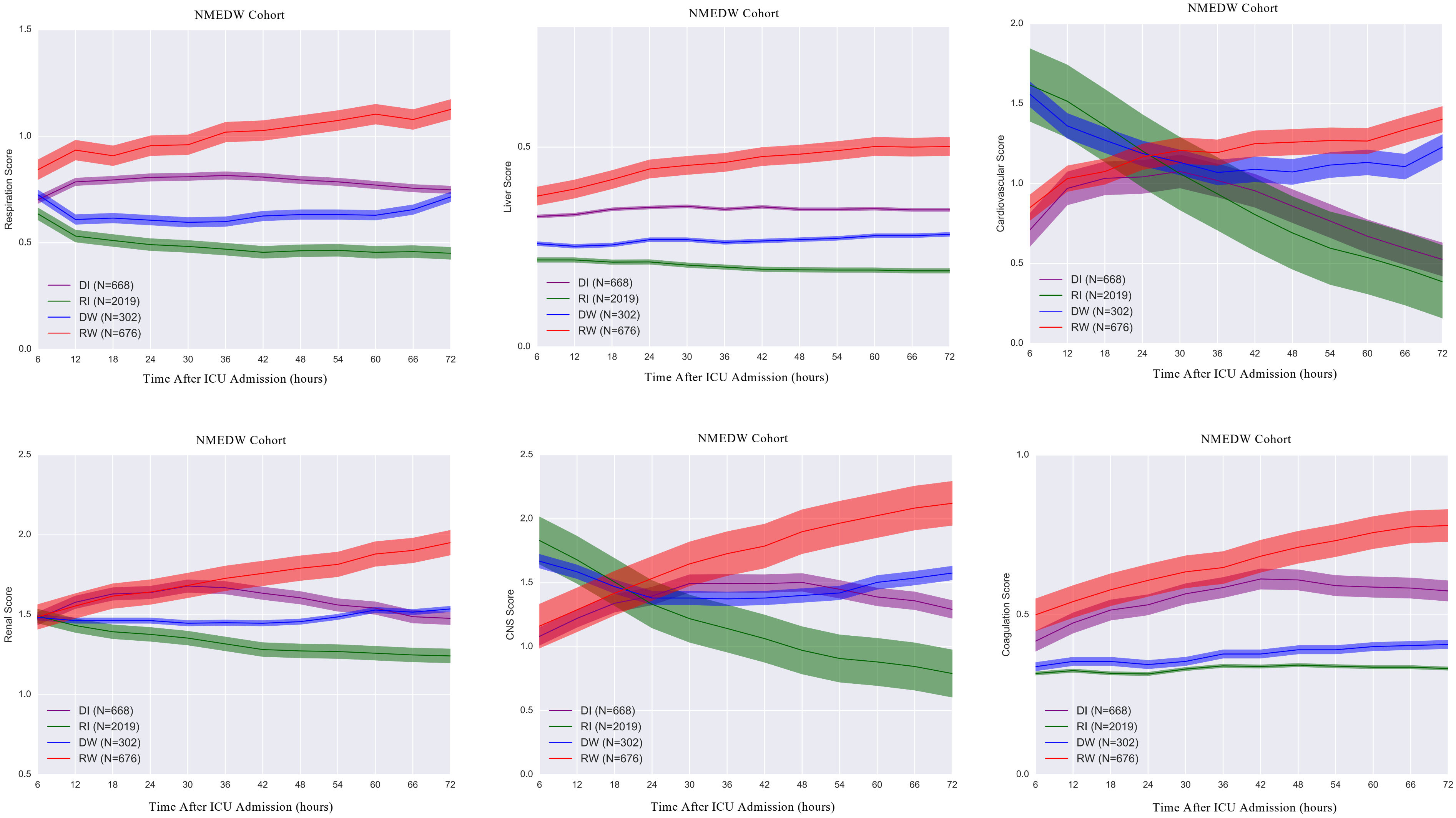

NMEDW Cohort
Respiration Score
DI (N=668)
RI (N=2019)
DW (N=302)
RW (N=676)
Time After ICU Admission (hours)
NMEDW Cohort
Liver Score
DI (N=668)
RI (N=2019)
DW (N=302)
RW (N=676)
Time After ICU Admission (hours)
NMEDW Cohort
Cardiovascular Score
DI (N=668)
RI (N=2019)
DW (N=302)
RW (N=676)
Time After ICU Admission (hours)
NMEDW Cohort
Renal Score
DI (N=668)
RI (N=2019)
DW (N=302)
RW (N=676)
Time After ICU Admission (hours)
NMEDW Cohort
CNS Score
DI (N=668)
RI (N=2019)
DW (N=302)
RW (N=676)
Time After ICU Admission (hours)
NMEDW Cohort
Coagulation Score
DI (N=668)
RI (N=2019)
DW (N=302)
RW (N=676)
Time After ICU Admission (hours)

Figure S4. The trajectories of the subphenotypes in terms of subscores in the NMEDW validation cohort. DI: Delayed Improving; RI: Rapidly Improving; DW: Delayed Worsening; RW: Rapidly Worsening.

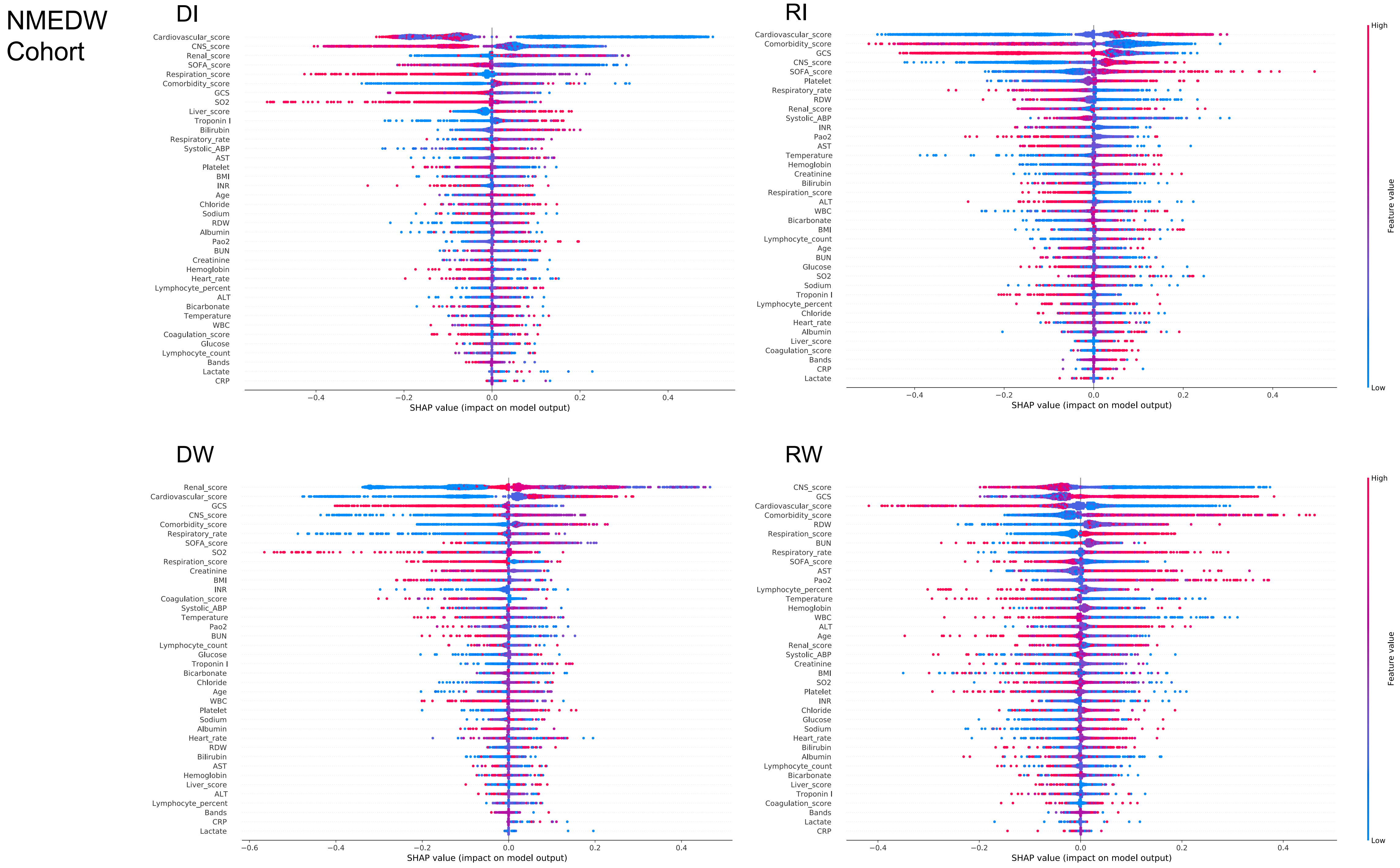
NMEDW
Cohort
DI
Cardiovascular_score
CNS_score
Renal_score
SOFA_score
Respiration_score
Comorbidity_score
GCS
SO2
Liver_score
Troponin I
Bilirubin
Respiratory_rate
Systolic_ABP
AST
Platelet
BMI
INR
Age
Chloride
Sodium
RDW
Albumin
Pao2
BUN
Creatinine
Hemoglobin
Heart_rate
Lymphocyte_percent
ALT
Bicarbonate
Temperature
WBC
Coagulation_score
Glucose
Lymphocyte_count
Bands
Lactate
CRP
−0.4
−0.2
0.0
0.2
0.4
SHAP value (impact on model output)
RI
Cardiovascular_score
Comorbidity_score
GCS
CNS_score
SOFA_score
Platelet
Respiratory_rate
RDW
Renal_score
Systolic_ABP
INR
Pao2
AST
Temperature
Hemoglobin
Creatinine
Bilirubin
Respiration_score
ALT
WBC
Bicarbonate
BMI
Lymphocyte_count
Age
BUN
Glucose
SO2
Sodium
Troponin I
Lymphocyte_percent
Chloride
Heart_rate
Albumin
Liver_score
Coagulation_score
Bands
CRP
Lactate
−0.4
−0.2
0.0
0.2
0.4
SHAP value (impact on model output)
High
Feature value
Low
DW
Renal_score
Cardiovascular_score
GCS
CNS_score
Comorbidity_score
Respiratory_rate
SOFA_score
SO2
Respiration_score
Creatinine
BMI
INR
Coagulation_score
Systolic_ABP
Temperature
Pao2
BUN
Lymphocyte_count
Glucose
Troponin I
Bicarbonate
Chloride
Age
WBC
Platelet
Sodium
Albumin
Heart_rate
RDW
Bilirubin
AST
Hemoglobin
Liver_score
ALT
Lymphocyte_percent
Bands
CRP
Lactate
−0.6
−0.4
−0.2
0.0
0.2
0.4
SHAP value (impact on model output)
RW
CNS_score
GCS
Cardiovascular_score
Comorbidity_score
RDW
Respiration_score
BUN
Respiratory_rate
SOFA_score
AST
Pao2
Lymphocyte_percent
Temperature
Hemoglobin
WBC
ALT
Age
Renal_score
Systolic_ABP
Creatinine
BMI
SO2
Platelet
INR
Chloride
Glucose
Sodium
Heart_rate
Bilirubin
Albumin
Lymphocyte_count
Bicarbonate
Liver_score
Troponin I
Coagulation_score
Bands
Lactate
CRP
−0.4
−0.2
0.0
0.2
0.4
SHAP value (impact on model output)
High
Feature value
Low

Figure S5. Predictor contribution to the prediction of the predictive model in the NMEDW validation cohort.
DI: Delayed Improving; RI: Rapidly Improving; DW: Delayed Worsening; RW: Rapidly Worsening.

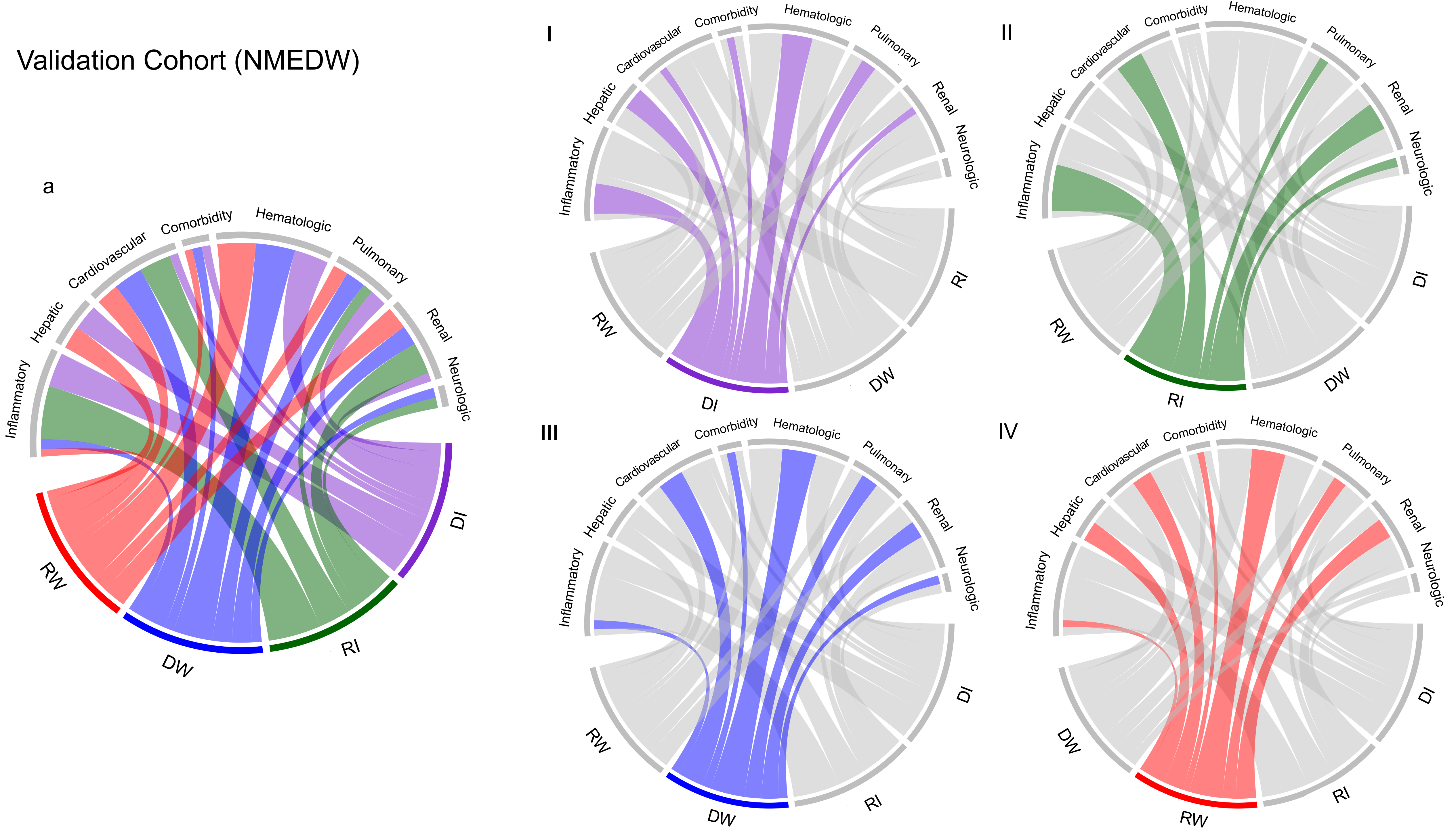
Validation Cohort (NMEDW)
a
Inflammatory
Hepatic
Cardiovascular
Comorbidity
Hematologic
Pulmonary
Renal
Neurologic
DI
RI
DW
RW
I
II
III
IV

Figure S6. Chord diagrams showing abnormal clinical variables by subphenotype in the NMEDW validation cohort. a: abnormal biomarkers vs. all subphenotypes; I: abnormal biomarkers vs. DI; II: abnormal biomarkers vs. RI; III: abnormal biomarkers vs. DW; IV: abnormal biomarkers vs. RW. DI: Delayed Improving; RI: Rapidly Improving; DW: Delayed Worsening; RW: Rapidly Worsening.

Validation Cohort (NMEDW)

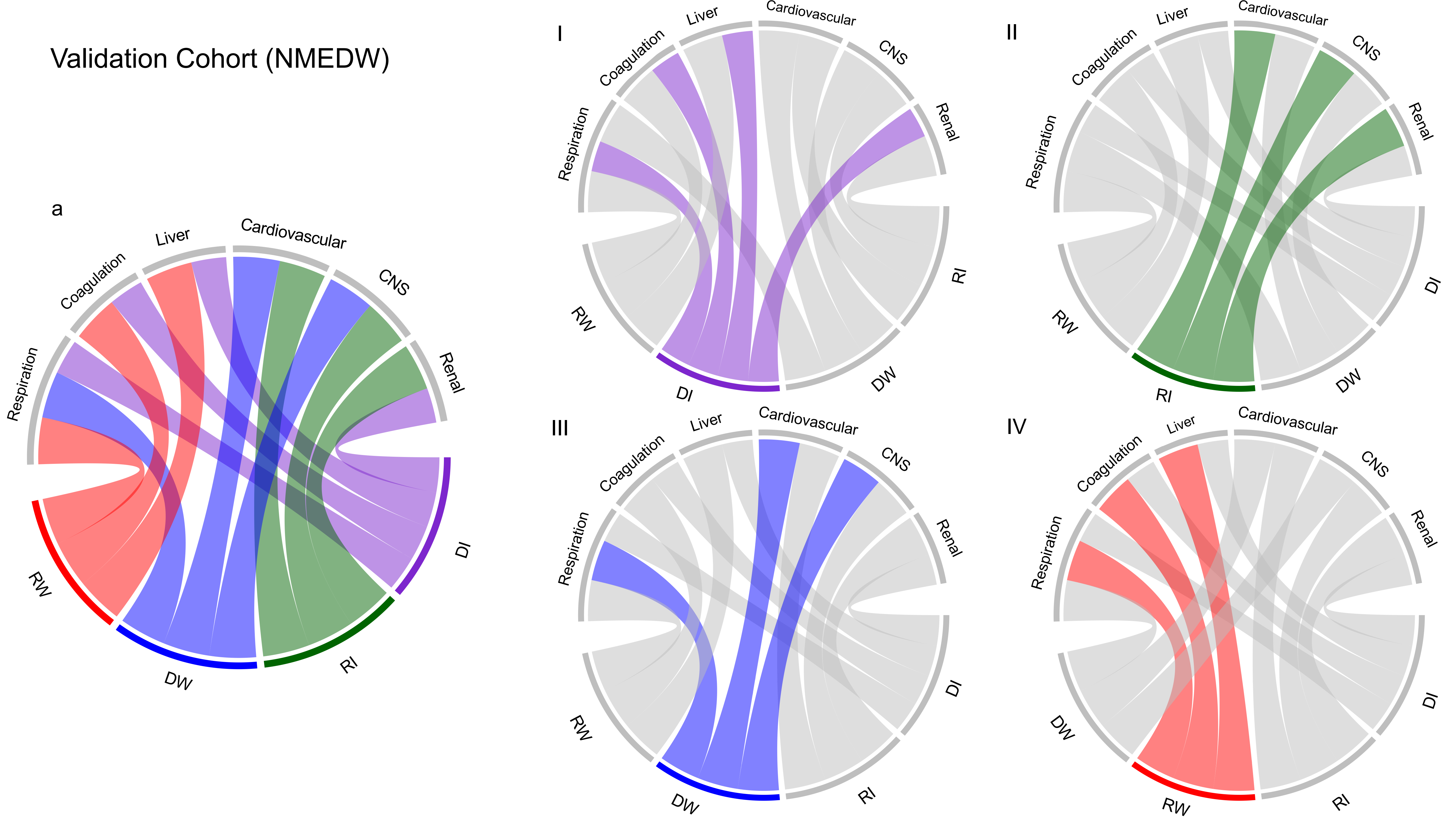

Figure S7. Chord diagrams showing abnormal subscores by subphenotype in the NMEDW validation cohort. a: abnormal subscores vs. all subphenotypes; I: abnormal subscores vs. DI; II: abnormal subscores vs. RI; III: abnormal subscores vs. DW; IV: abnormal subscores vs. RW. DI: Delayed Improving; RI: Rapidly Improving; DW: Delayed Worsening; RW: Rapidly Worsening.

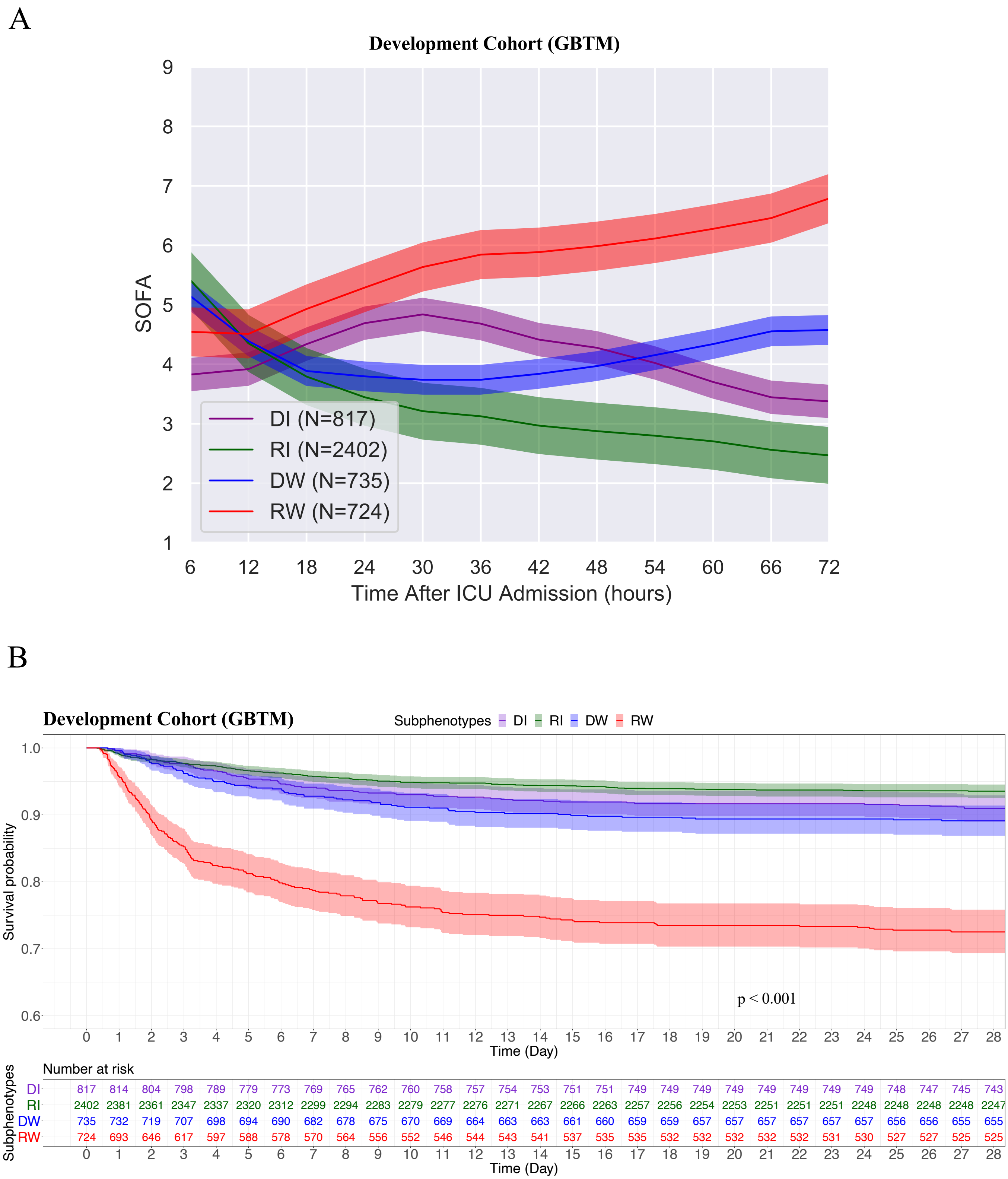
A
Development Cohort (GBTM)
SOFA
Time After ICU Admission (hours)
DI (N=817)
RI (N=2402)
DW (N=735)
RW (N=724)
B
Development Cohort (GBTM)
Subphenotypes DI RI DW RW
Survival probability
p < 0.001
Time (Day)
Number at risk
Subphenotypes
DI 817 814 804 798 789 779 773 769 765 762 760 758 757 754 753 751 751 749 749 749 749 749 749 749 749 748 747 745 743
RI 2402 2381 2361 2347 2337 2320 2312 2299 2294 2283 2279 2277 2276 2271 2267 2266 2263 2257 2256 2254 2253 2251 2251 2251 2248 2248 2248 2248 2247
DW 735 732 719 707 698 694 690 682 678 675 670 669 664 663 663 661 660 659 659 657 657 657 657 657 657 656 656 655 655
RW 724 693 646 617 597 588 578 570 564 556 552 546 544 543 541 537 535 535 532 532 532 532 532 531 530 527 527 525 525
Time (Day)

Figure S8. Sequential Organ Failure Assessment (SOFA) trajectories of the subphenotypes and survival analysis in terms of the identified subphenotypes by GBTM. The (A) describes the SOFA trajectories of the subphenotypes re-derived by GBTM in development cohort. The (B) shows the survival analysis results in subphenotypes re-derived by GBTM in the development cohort. DI: Delayed Improving; RI: Rapidly Improving; DW: Delayed Worsening; RW: Rapidly Worsening.

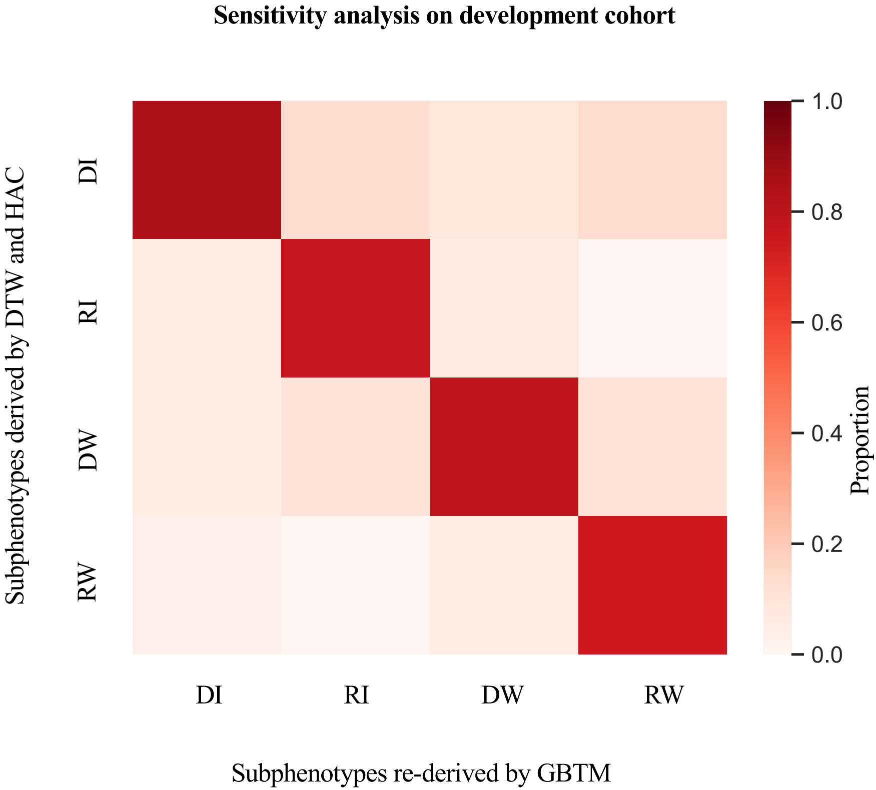

Sensitivity analysis on development cohort
Subphenotypes derived by DTW and HAC
DI
RI
DW
RW
Subphenotypes re-derived by GBTM
DI
RI
DW
RW
Proportion
1.0
0.8
0.6
0.4
0.2
0.0

Figure S9. Confusion matrices for comparing the subphenotypes obtained by DTW and HAC, and GBTM in development cohort. DI: Delayed Improving; RI: Rapidly Improving; DW: Delayed Worsening; RW: Rapidly Worsening. DTW: Dynamic Time Warping; HAC: Hierarchical Agglomerative Clustering. GBTM: Group-Based Trajectory Modeling.

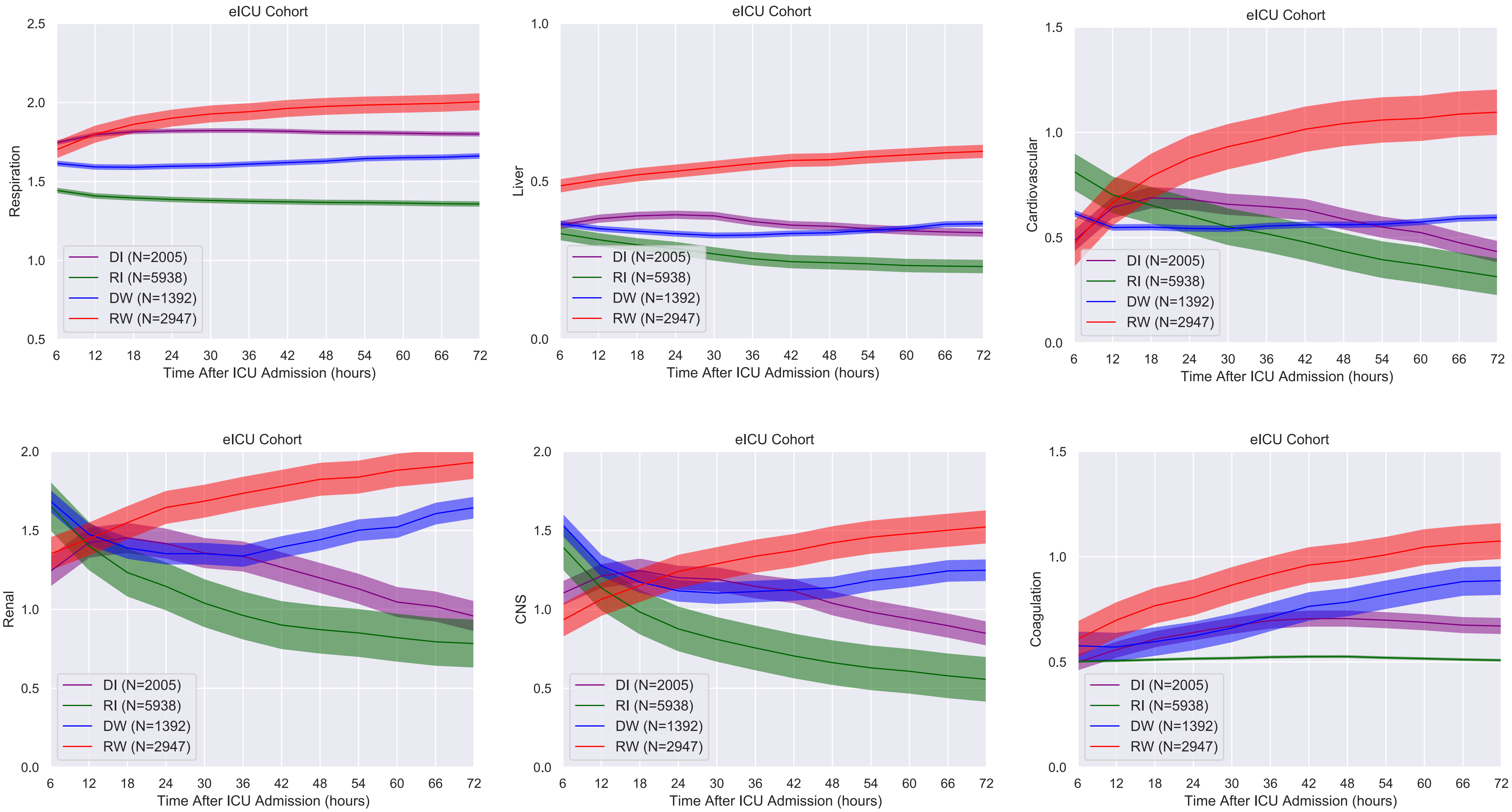
eICU Cohort
Respiration
Liver
Cardiovascular
Renal
CNS
Coagulation
Time After ICU Admission (hours)
DI (N=2005)
RI (N=5938)
DW (N=1392)
RW (N=2947)

Figure S10. The trajectories of the subphenotypes in terms of subscores in the eICU validation cohort. DI: Delayed Improving; RI: Rapidly Improving; DW: Delayed Worsening; RW: Rapidly Worsening.

eICU Cohort

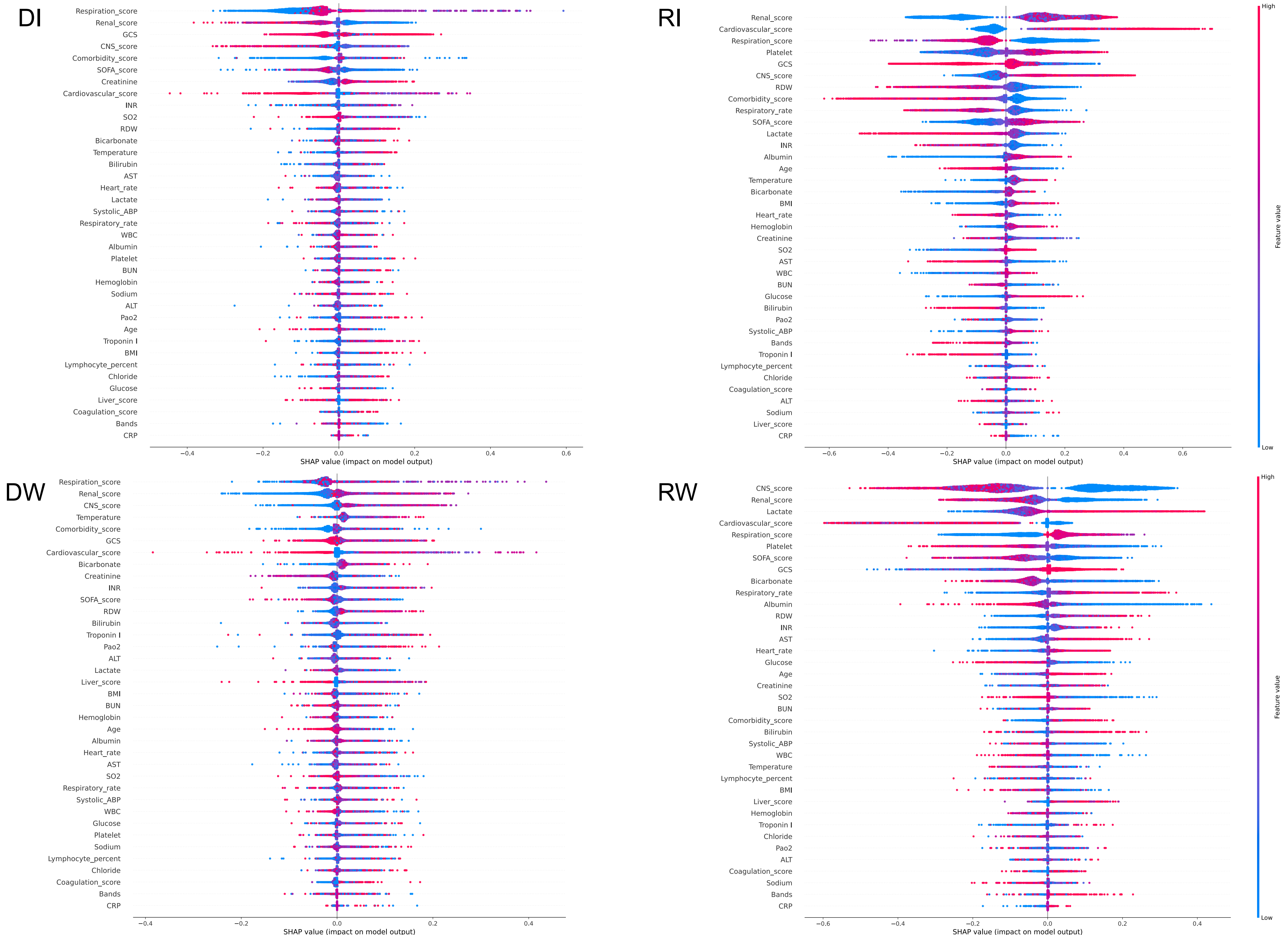
DI
Respiration_score
Renal_score
GCS
CNS_score
Comorbidity_score
SOFA_score
Creatinine
Cardiovascular_score
INR
SO2
RDW
Bicarbonate
Temperature
Bilirubin
AST
Heart_rate
Lactate
Systolic_ABP
Respiratory_rate
WBC
Albumin
Platelet
BUN
Hemoglobin
Sodium
ALT
Pao2
Age
Troponin I
BMI
Lymphocyte_percent
Chloride
Glucose
Liver_score
Coagulation_score
Bands
CRP
−0.4
−0.2
0.0
0.2
0.4
0.6
SHAP value (impact on model output)
RI
Renal_score
Cardiovascular_score
Respiration_score
Platelet
GCS
CNS_score
RDW
Comorbidity_score
Respiratory_rate
SOFA_score
Lactate
INR
Albumin
Age
Temperature
Bicarbonate
BMI
Heart_rate
Hemoglobin
Creatinine
SO2
AST
WBC
BUN
Glucose
Bilirubin
Pao2
Systolic_ABP
Bands
Troponin I
Lymphocyte_percent
Chloride
Coagulation_score
ALT
Sodium
Liver_score
CRP
−0.6
−0.4
−0.2
0.0
0.2
0.4
0.6
SHAP value (impact on model output)
High
Feature value
Low
DW
Respiration_score
Renal_score
CNS_score
Temperature
Comorbidity_score
GCS
Cardiovascular_score
Bicarbonate
Creatinine
INR
SOFA_score
RDW
Bilirubin
Troponin I
Pao2
ALT
Lactate
Liver_score
BMI
BUN
Hemoglobin
Age
Albumin
Heart_rate
AST
SO2
Respiratory_rate
Systolic_ABP
WBC
Glucose
Platelet
Sodium
Lymphocyte_percent
Chloride
Coagulation_score
Bands
CRP
−0.4
−0.2
0.0
0.2
0.4
SHAP value (impact on model output)
RW
CNS_score
Renal_score
Lactate
Cardiovascular_score
Respiration_score
Platelet
SOFA_score
GCS
Bicarbonate
Respiratory_rate
Albumin
RDW
INR
AST
Heart_rate
Glucose
Age
Creatinine
SO2
BUN
Comorbidity_score
Bilirubin
Systolic_ABP
WBC
Temperature
Lymphocyte_percent
BMI
Liver_score
Hemoglobin
Troponin I
Chloride
Pao2
ALT
Coagulation_score
Sodium
Bands
CRP
−0.6
−0.4
−0.2
0.0
0.2
0.4
SHAP value (impact on model output)
High
Feature value
Low

Figure S11. Predictor contribution to the prediction of the predictive model in the eICU validation cohort. DI: Delayed Improving; RI: Rapidly Improving; DW: Delayed Worsening; RW: Rapidly Worsening.

Validation Cohort (eICU)

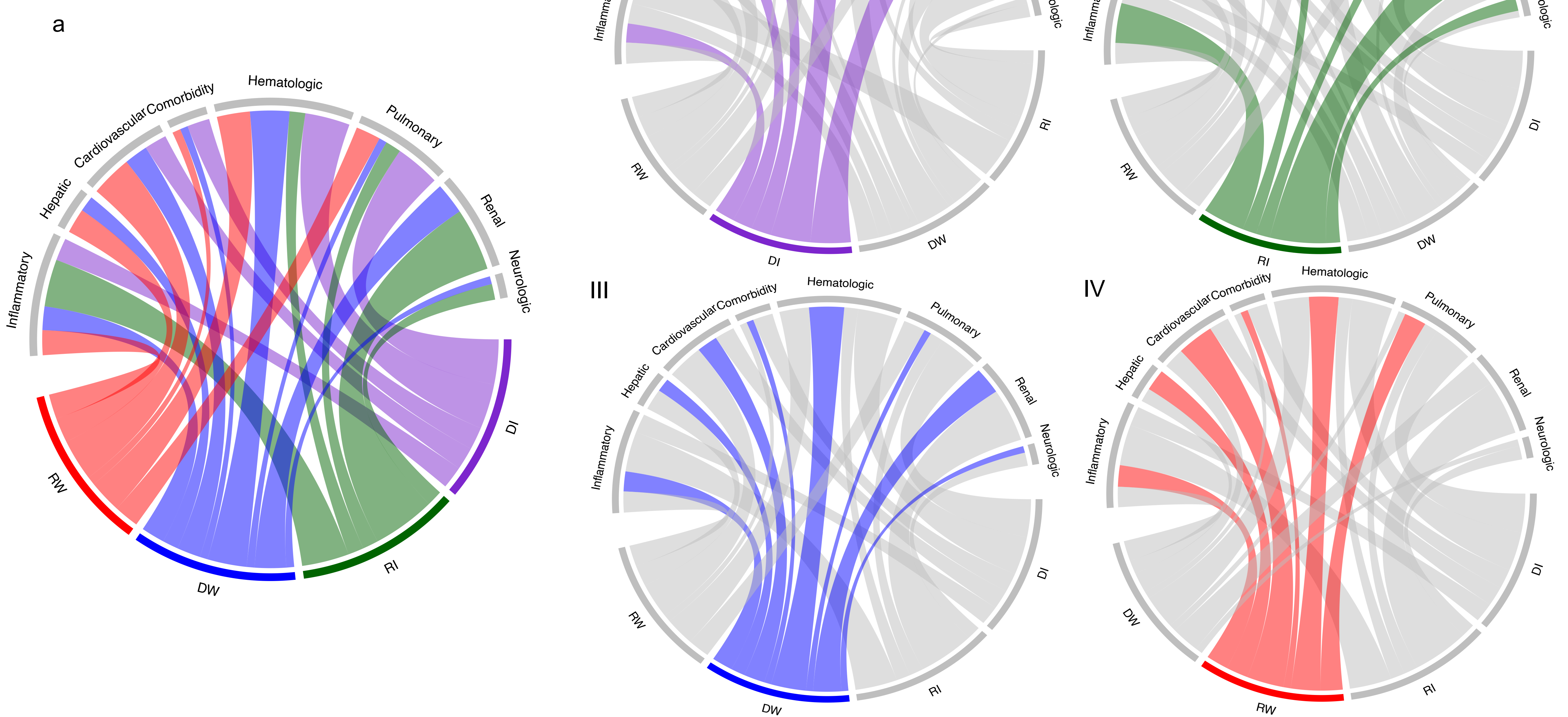

Figure S12. Chord diagrams showing abnormal clinical variables by subphenotype in the eICU validation cohort. a: abnormal biomarkers vs. all subphenotypes; I: abnormal biomarkers vs. DI; II: abnormal biomarkers vs. RI; III: abnormal biomarkers vs. DW; IV: abnormal biomarkers vs. RW. DI: Delayed Improving; RI: Rapidly Improving; DW: Delayed Worsening; RW: Rapidly Worsening.

Validation Cohort (eICU)

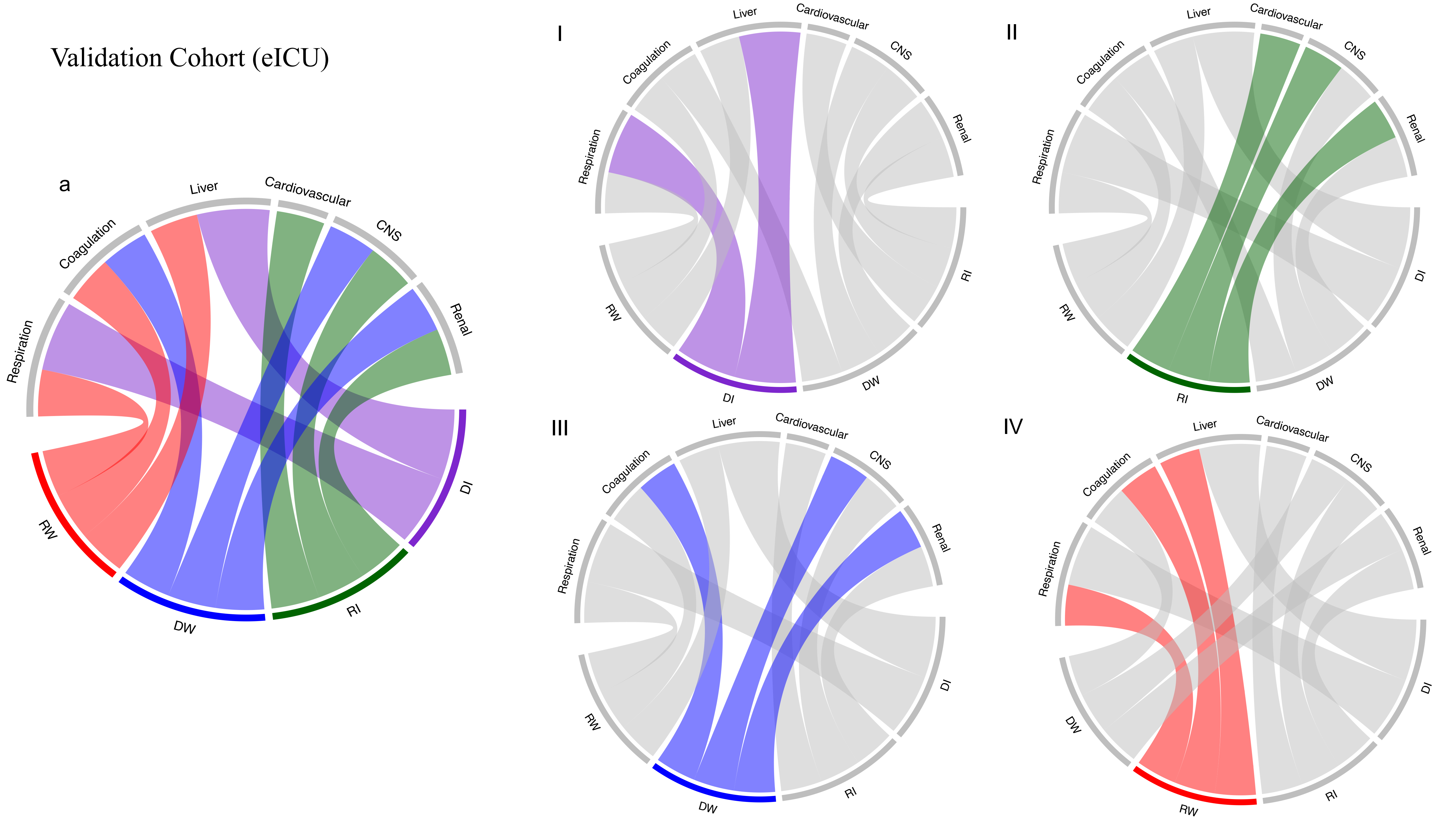

Figure S13. Chord diagrams showing abnormal subscores by subphenotype in the eICU validation cohort. a: abnormal subscores vs. all subphenotypes; I: abnormal subscores vs. DI; II: abnormal subscores vs. RI; III: abnormal subscores vs. DW; IV: abnormal subscores vs. RW. DI: Delayed Improving; RI: Rapidly Improving; DW: Delayed Worsening; RW: Rapidly Worsening.

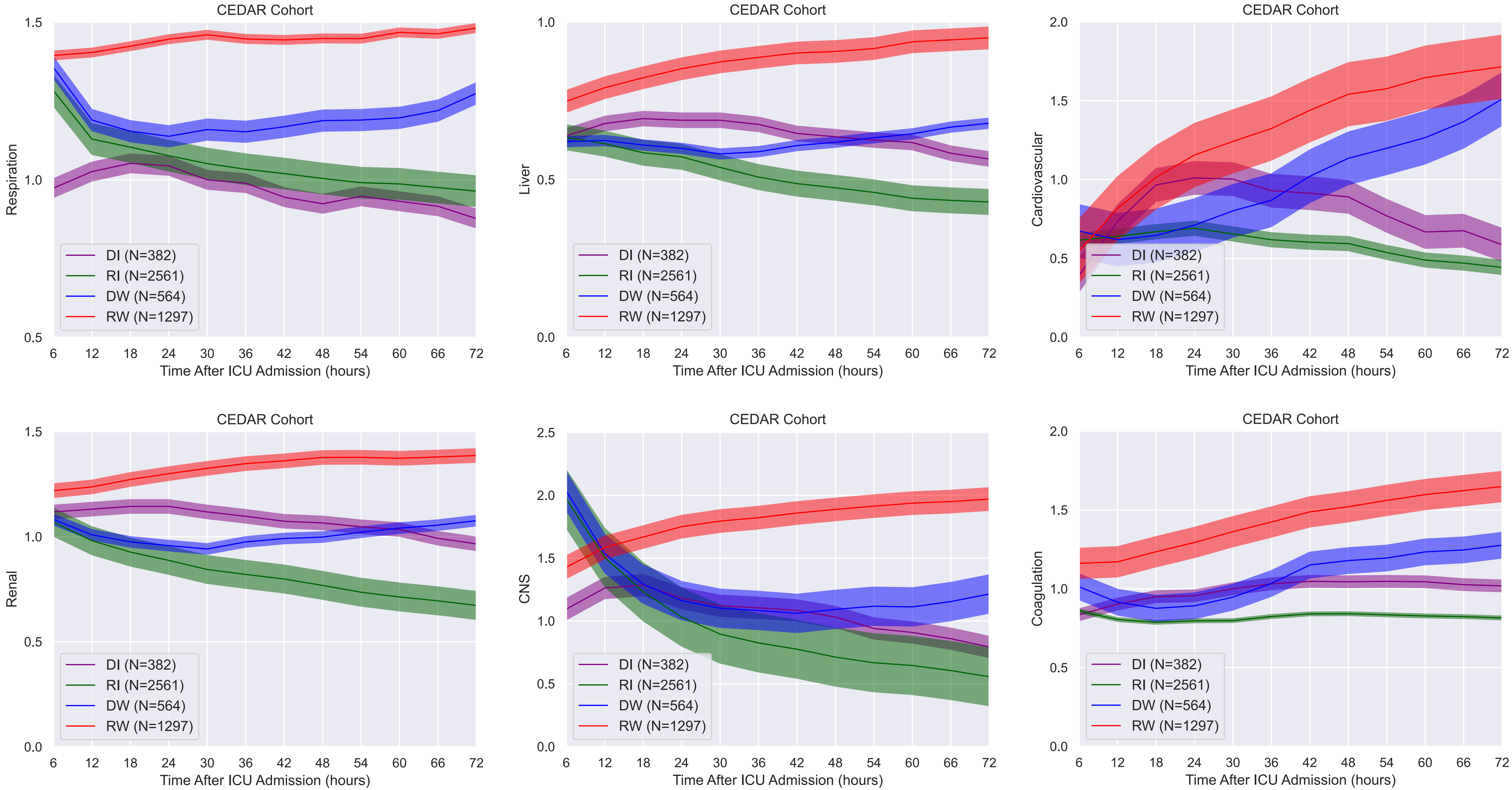
CEDAR Cohort
Respiration
Liver
Cardiovascular
Renal
CNS
Coagulation
Time After ICU Admission (hours)
DI (N=382)
RI (N=2561)
DW (N=564)
RW (N=1297)

Figure S14. The trajectories of the subphenotypes in terms of subscores in the CEDAR validation cohort. DI: Delayed Improving; RI: Rapidly Improving; DW: Delayed Worsening; RW: Rapidly Worsening.

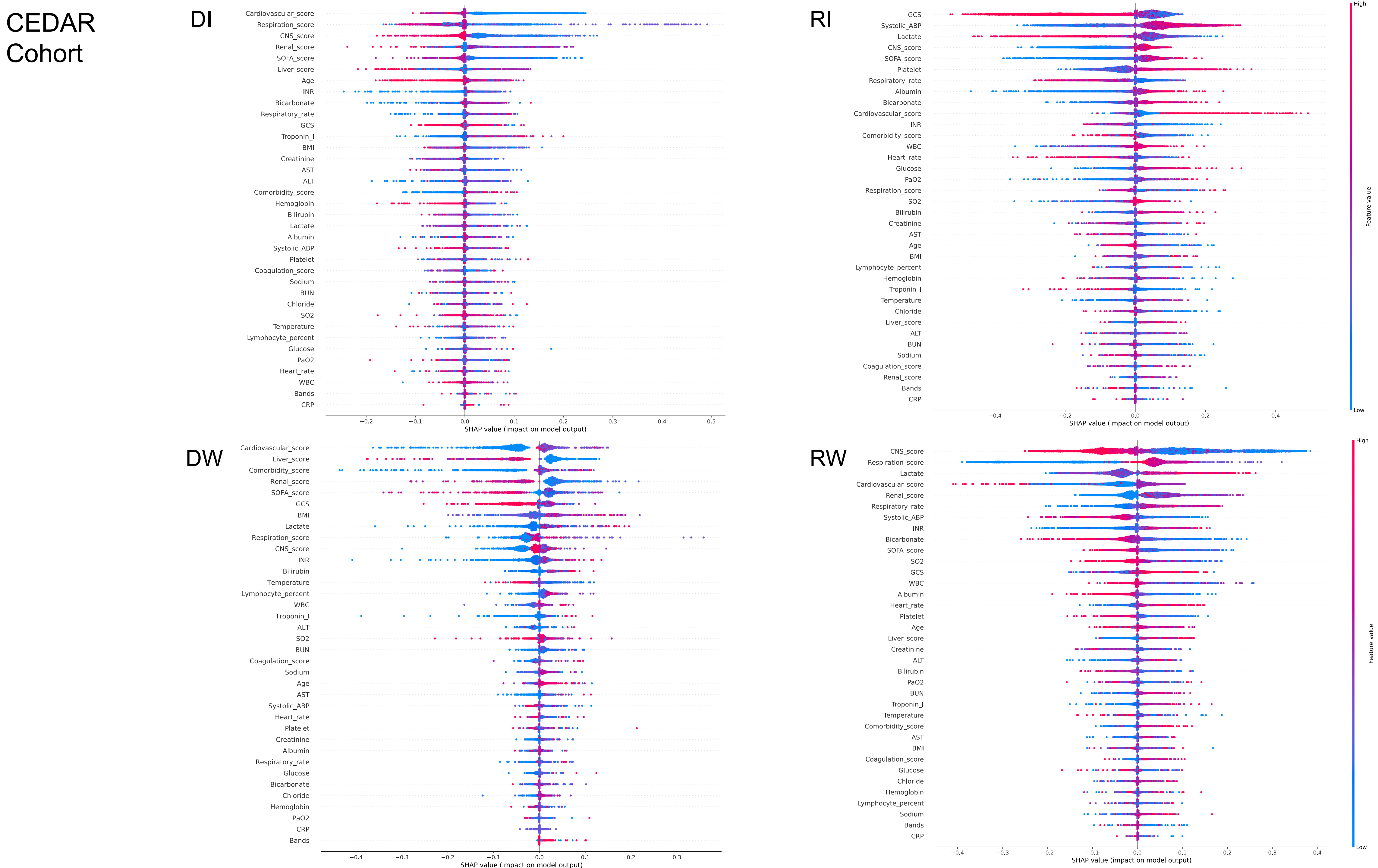
CEDAR Cohort
DI
Cardiovascular_score
Respiration_score
CNS_score
Renal_score
SOFA_score
Liver_score
Age
INR
Bicarbonate
Respiratory_rate
GCS
Troponin_I
BMI
Creatinine
AST
ALT
Comorbidity_score
Hemoglobin
Bilirubin
Lactate
Albumin
Systolic_ABP
Platelet
Coagulation_score
Sodium
BUN
Chloride
SO2
Temperature
Lymphocyte_percent
Glucose
PaO2
Heart_rate
WBC
Bands
CRP
−0.2 −0.1 0.0 0.1 0.2 0.3 0.4 0.5
SHAP value (impact on model output)
RI
GCS
Systolic_ABP
Lactate
CNS_score
SOFA_score
Platelet
Respiratory_rate
Albumin
Bicarbonate
Cardiovascular_score
INR
Comorbidity_score
WBC
Heart_rate
Glucose
PaO2
Respiration_score
SO2
Bilirubin
Creatinine
AST
Age
BMI
Lymphocyte_percent
Hemoglobin
Troponin_I
Temperature
Chloride
Liver_score
ALT
BUN
Sodium
Coagulation_score
Renal_score
Bands
CRP
−0.4 −0.2 0.0 0.2 0.4
SHAP value (impact on model output)
High
Feature value
Low
DW
Cardiovascular_score
Liver_score
Comorbidity_score
Renal_score
SOFA_score
GCS
BMI
Lactate
Respiration_score
CNS_score
INR
Bilirubin
Temperature
Lymphocyte_percent
WBC
Troponin_I
ALT
SO2
BUN
Coagulation_score
Sodium
Age
AST
Systolic_ABP
Heart_rate
Platelet
Creatinine
Albumin
Respiratory_rate
Glucose
Bicarbonate
Chloride
Hemoglobin
PaO2
CRP
Bands
−0.4 −0.3 −0.2 −0.1 0.0 0.1 0.2 0.3
SHAP value (impact on model output)
RW
CNS_score
Respiration_score
Lactate
Cardiovascular_score
Renal_score
Respiratory_rate
Systolic_ABP
INR
Bicarbonate
SOFA_score
SO2
GCS
WBC
Albumin
Heart_rate
Platelet
Age
Liver_score
Creatinine
ALT
Bilirubin
PaO2
BUN
Troponin_I
Temperature
Comorbidity_score
AST
BMI
Coagulation_score
Glucose
Chloride
Hemoglobin
Lymphocyte_percent
Sodium
Bands
CRP
−0.4 −0.3 −0.2 −0.1 0.0 0.1 0.2 0.3 0.4
SHAP value (impact on model output)
High
Feature value
Low

Figure S15. Predictor contribution to the prediction of the predictive model in the CEDAR validation cohort.
DI: Delayed Improving; RI: Rapidly Improving; DW: Delayed Worsening; RW: Rapidly Worsening.

Validation Cohort (CEDAR)

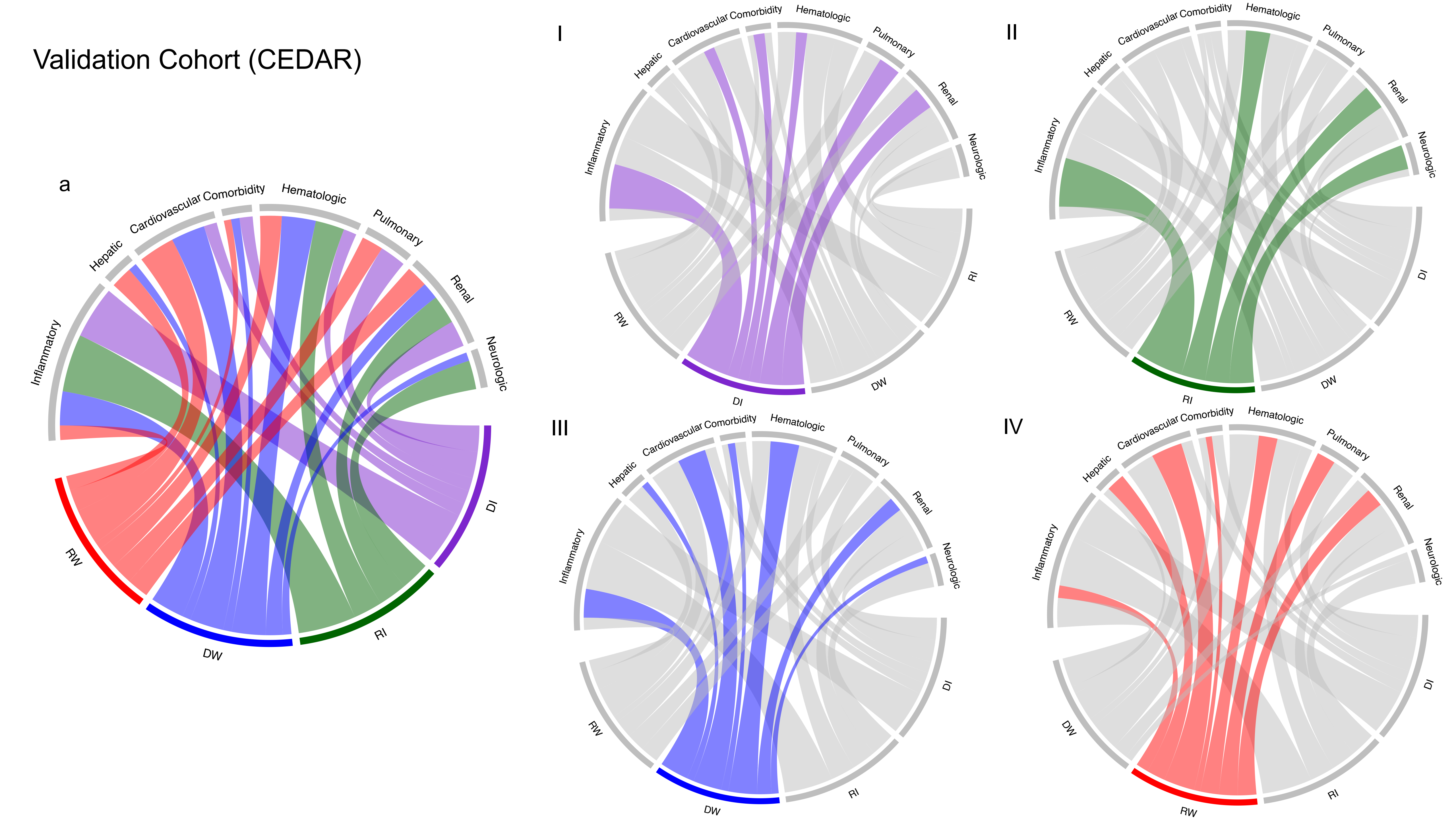

Figure S16. Chord diagrams showing abnormal clinical variables by subphenotype in the CEDAR validation cohort. a: abnormal biomarkers vs. all subphenotypes; I: abnormal biomarkers vs. DI; II: abnormal biomarkers vs. RI; III: abnormal biomarkers vs. DW; IV: abnormal biomarkers vs. RW. DI: Delayed Improving; RI: Rapidly Improving; DW: Delayed Worsening; RW: Rapidly Worsening.

Validation Cohort (CEDAR)

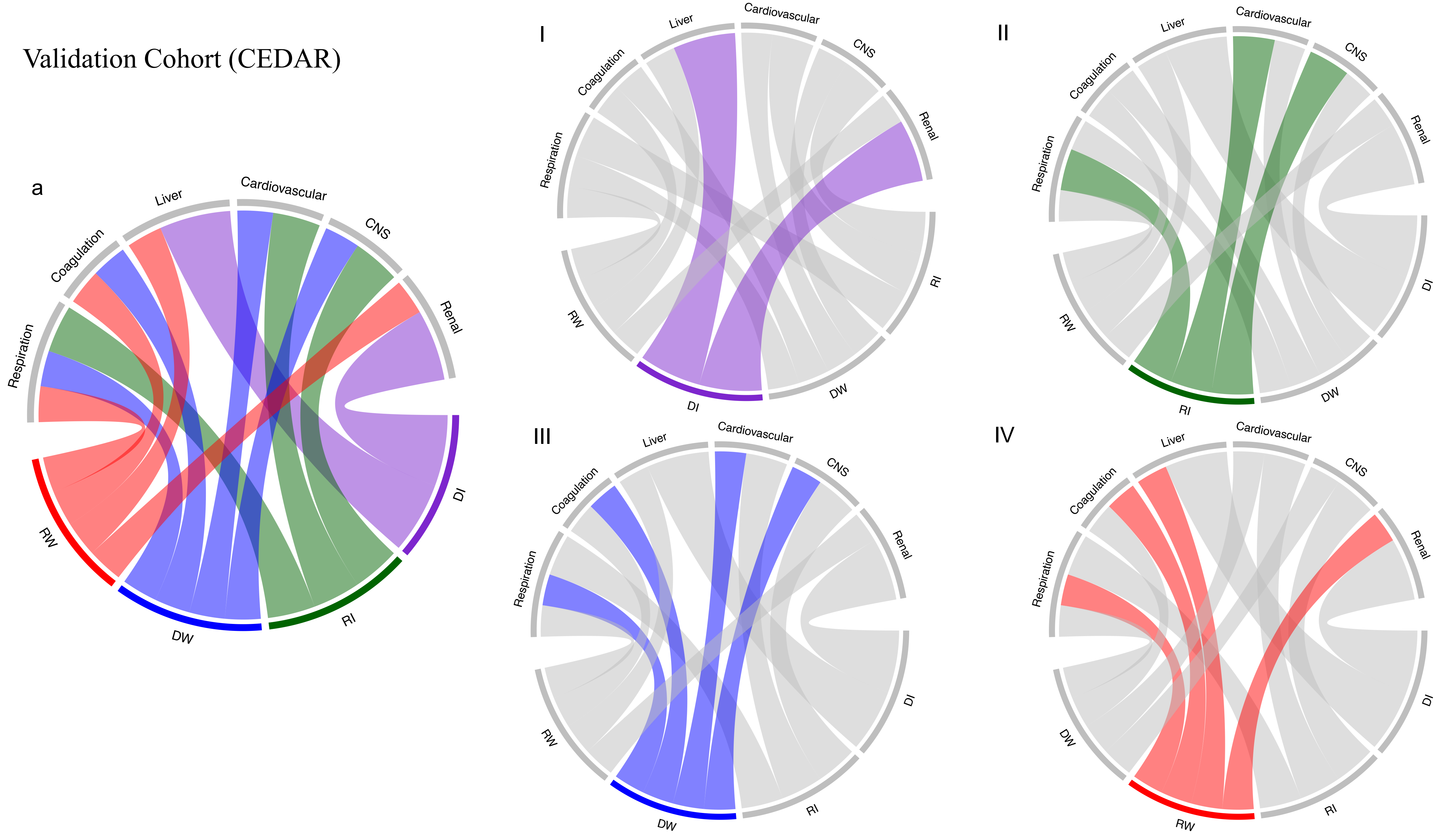

Figure S17. Chord diagrams showing abnormal subscores by subphenotype in the CEDAR validation cohort. a: abnormal subscores vs. all subphenotypes; I: abnormal subscores vs. DI; II: abnormal subscores vs. RI; III: abnormal subscores vs. DW; IV: abnormal subscores vs. RW. DI: Delayed Improving; RI: Rapidly Improving; DW: Delayed Worsening; RW: Rapidly Worsening.

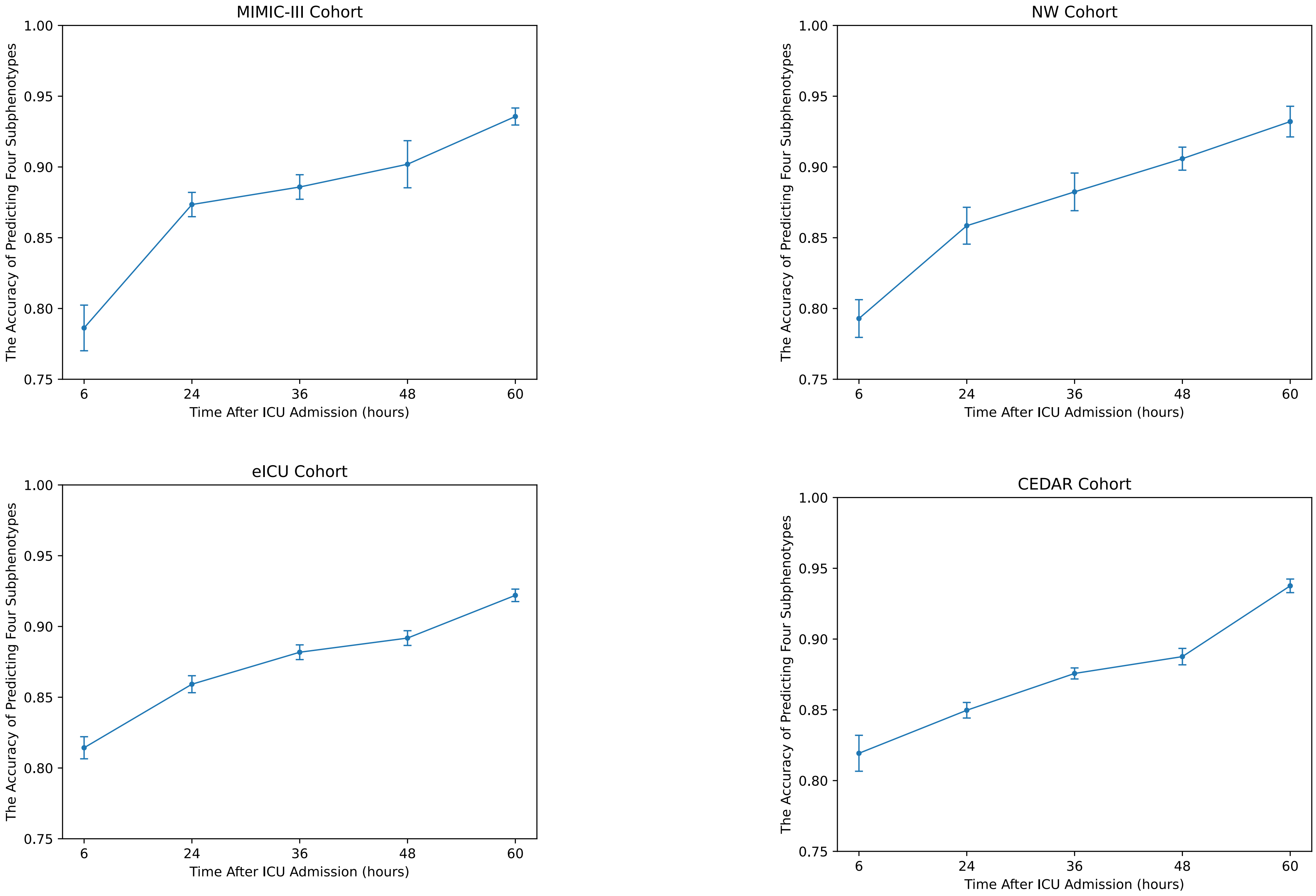
MIMIC-III Cohort
NW Cohort
eICU Cohort
CEDAR Cohort
The Accuracy of Predicting Four Subphenotypes
Time After ICU Admission (hours)
1.00
0.95
0.90
0.85
0.80
0.75
6
24
36
48
60

Figure S18. The accuracy of predicting four subphenotypes at successive time points (hours 6, 24, 36, 48, 60) after ICU admission in development and validation cohorts.

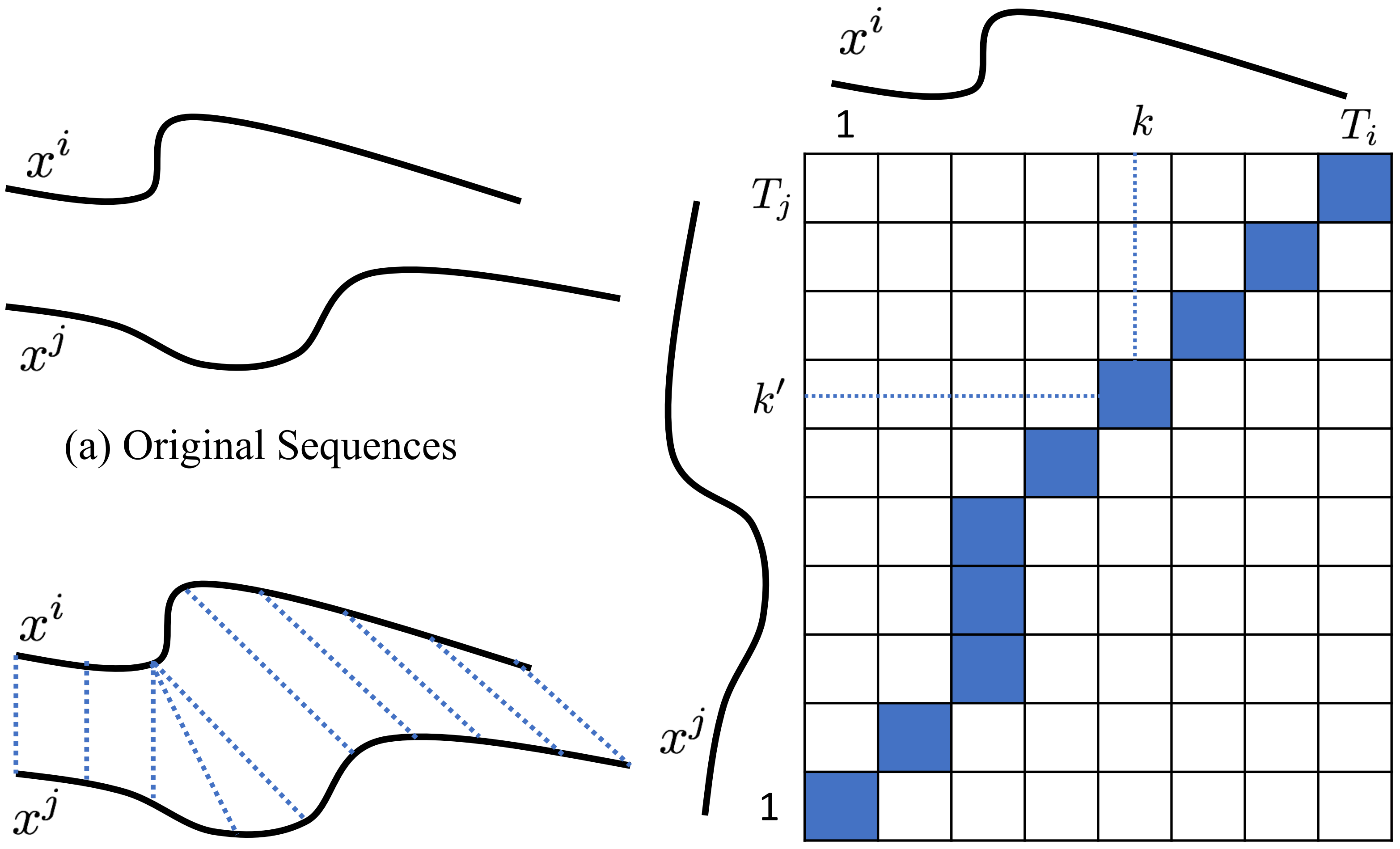

(a) Original Sequences

(c) DTW Matching

(b) Warping Path

Figure S19. The illustration of using DTW during obtaining matched sequences. (c) shows the warping path induced by the DTW matching, where the blue cells indicate the best matching pairs for the points in the two sequences.

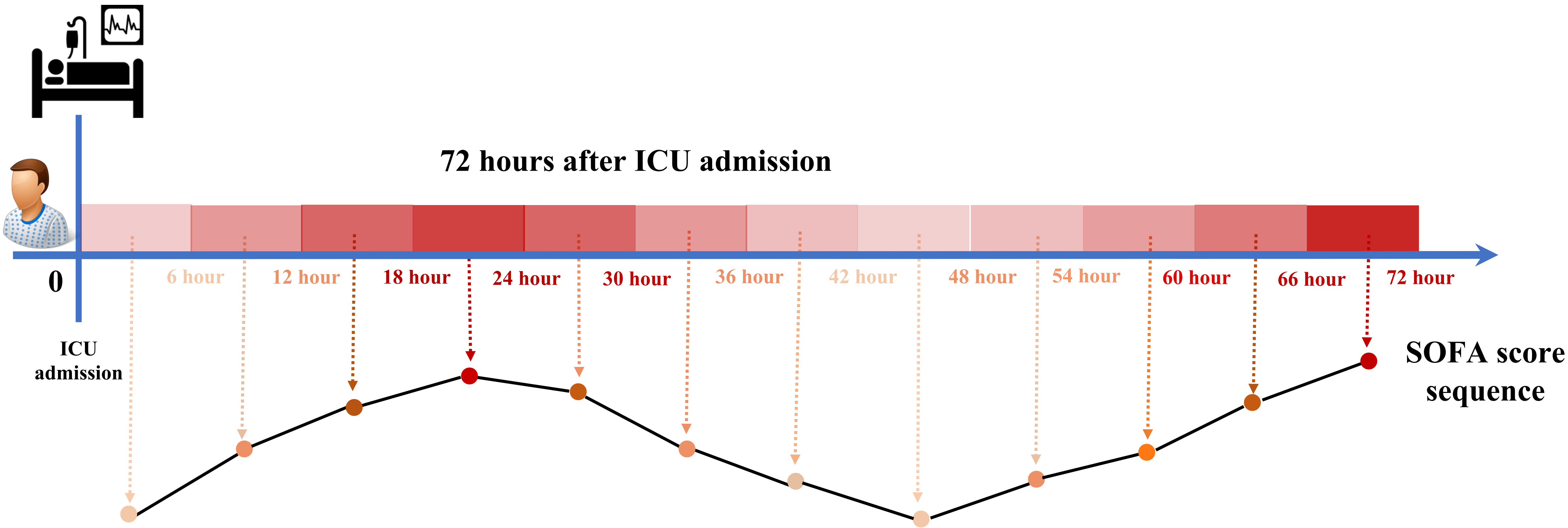
72 hours after ICU admission
0
6 hour
12 hour
18 hour
24 hour
30 hour
36 hour
42 hour
48 hour
54 hour
60 hour
66 hour
72 hour
ICU admission
SOFA score sequence

Figure S20. An example: the illustration of patient's SOFA trajectory.
